# An oxidative amidation and heterocyclization approach for the synthesis of β-carbolines and dihydroeudistomin Y

**DOI:** 10.3762/bjoc.10.45

**Published:** 2014-02-25

**Authors:** Suresh Babu Meruva, Akula Raghunadh, Raghavendra Rao Kamaraju, U K Syam Kumar, P K Dubey

**Affiliations:** 1 Technology Development Centre, Custom Pharmaceutical Services, Dr. Reddy’s Laboratories Ltd., Miyapur, Hyderabad – 500049, India; 2 Department of Chemistry, College of Engineering, JNTUH, Kukatpally, Hyderabad – 500085, India

**Keywords:** Bischler–Napieralski reaction, dihydro-β-carboline derivatives, dihydro-eudistomin, eudistomin, keto amide, oxidative amidation

## Abstract

A novel synthetic methodology has been developed for the synthesis of dihydro-β-carboline derivatives employing oxidative amidation–Bischler–Napieralski reaction conditions using tryptamine and 2,2-dibromo-1-phenylethanone as key starting materials. A number of dihydro-β-carboline derivatives have been synthesized in moderate to good yields using this methodology. Attempts were made towards the conversion of these dihydro-β-carbolines to naturally occurring eudistomin alkaloids.

## Introduction

**Figure 1:**
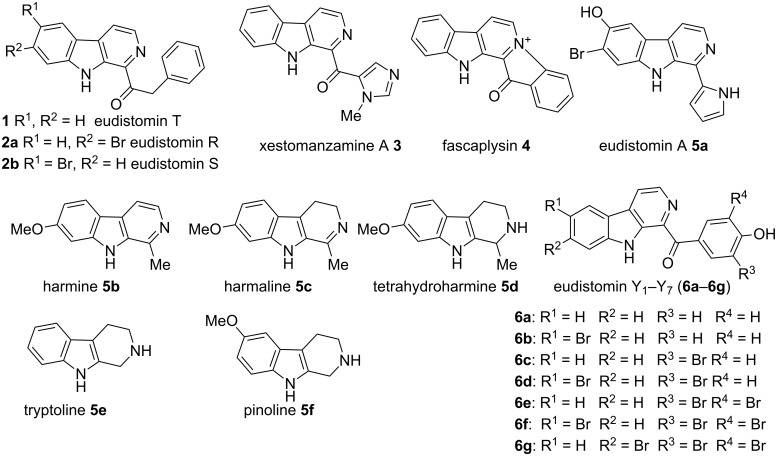
Natural products containing the β-carboline skeletal.

β-Carboline alkaloids [1] are widespread in plants, animals and some are formed naturally in the biological system. Rinehart et al. [2] reported the isolation of β-carboline alkaloids such as eudistomins [3] and several of its analogues from the active Caribbean colonial tunicate *Eudistoma olivaceum.* β-Carboline alkaloids bearing a substituted phenylacetyl group at C-1 position such as eudistomin T (**1**) [4], eudistomin R (**2a**) and eudistomin S (**2b**) [5] were isolated by Cardellina et al. [6], and these compounds exhibit antimicrobial activity. Xestomanzamine A (**3**) [5] is a β-carboline alkaloid with an 1-methyl-1*H*-imidazole-5-acyl group at the C-1 position and was isolated from the Okinawan marine sponge *Xestopongia sp.* The other β-carboline alkaloids reported in the literature are fascaplysin (**4**) [7], eudistomin A (**5a**) [3], harmine (**5b**) [8–10], harmaline (**5c**) [11], and tetrahydroharmine (**5d**). Among these, methoxy substituted β-carboline alkaloids such as **5b**, **5c**, and **5d** are indigenously used as hallucinogenic drugs. Some β-carbolines, notably tryptoline (**5e**) and pinoline (**5f**), are produced naturally in the human body. Recently Heonjoong et al. isolated new β-carboline-based metabolites, designated as eudistomins Y_1_−Y_7_ (**6a**–**g**) [12–14] (Figure 1), from the tunicate of the genus *Eudistoma* and posses a benzoyl group at the C-1 position of the β-carboline structural framework. Eudistomins Y_1_−Y_7_ (**6a**–**6g**) were evaluated for their antibacterial activity. Eudistomin Y_6_ (**6f**) exhibits moderate antibacterial activity against the Gram-positive bacteria *Staphylococcus epidermis* and *Bacillus subtilis*.

## Results and Discussion

There are several approaches known in literature for the synthesis of β-carbolines. Most of the syntheses of eudistomin T (**1**) are generally carried out either from indole or its suitably substituted derivatives. The acylation of 2-(3-indolyl)ethyl isocyanide with phenylacetyl chloride followed by cyclization and aromatization is well documented in the literature for the synthesis of 1-benzoyldihydro-β-carbolines. The cyclization of the adduct, formed by the reaction of tryptamine with appropriately substituted 1,2,3-tricarbonyl compounds or with glyoxylic acid derivatives under Pictet–Spengler conditions is also reported in literature for the syntheses of eudistomin T (**1**) and eudistomin S (**2b**). Jenkins and co-workers reported the synthesis of fascaplysin (**4**) by the reaction of tryptamine with phenylacetyl chloride and carried out the aromatization under photo-oxidation conditions [15–16]. Lindsley and co-workers [17] reported the synthesis of eudistomins Y_1_−Y_7_ (**6a**–**6g**) under microwave conditions [18]. Considering the complexity involved in the synthesis of several of the starting materials used in the preparation of carbolines, especially tricarbonyl compounds, an alternate approach for the synthesis of 3*H*-β-carboline is sought after. Herein we described our successful efforts towards the synthesis of 1-benzoyldihydro-β-carbolines or dihydroeudistomin. The previously developed synthetic methodologies in our laboratory [19–24] were utilized for the synthesis of eudistomin Y (**6**) and its analogues

**Scheme 1:**
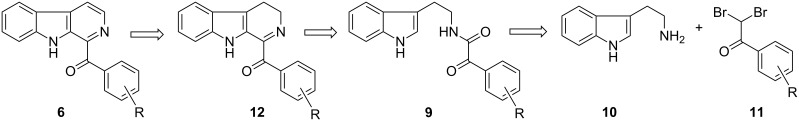
Retrosynthetic analysis of **6**.

The disconnection approach employed in the synthesis of 1-benzoyl*-*β-carboline is described in Scheme 1. Accordingly eudistomin Y (**6**) could be obtained by the aromatization of dihydro-β-carboline **12**. The dihydro-β-carboline **12** in turn could be synthesized from ketoamide **9** under Lewis acid mediated Bischler–Napieralski reaction. The key intermediate, ketoamide **9** required for the synthesis could easily be accessed from tryptamine (**10**) and appropriately substituted 2,2-dibromo-1-phenylethanone (**11**) [25–26] under oxidative amidation conditions.

**Scheme 2:**
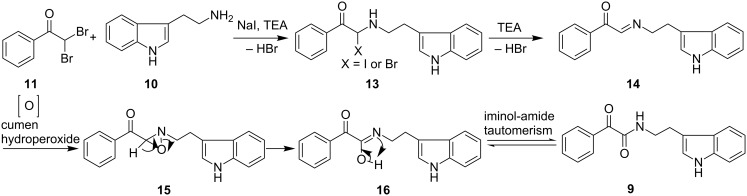
Plausible mechanism of the oxidative amidation for **9**.

**Scheme 3:**
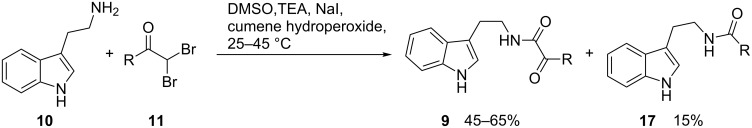
Synthesis of *α-*ketoamide **9**.

The oxidative amidation strategy employed in the current synthesis is previously reported from our group by the reaction of a secondary amine with aryl-2,2-dibromo-1-ethanone under aerial oxidation conditions [24]. Later this methodology was successfully employed in the synthesis of isoquinoline alkaloids [27]. The synthesis of eudistomin Y (**6**) was initiated with the conversion of the respective 2,2-dibromo-1-phenylethanone to the corresponding Schiff base **14** by reaction with tryptamine in presence of NaI. The Schiff base on in situ oxidation with cumene hydroperoxide afforded an unstable oxaziridine derivative **15**. Ring opening of the oxaziridine derivative **15** in presence of base afforded ketoiminol **16**, which on iminol–amide tautomerism provided the required *α*-ketoamide **9** (Scheme 2). It was possible to limit the formation of benzamide impurity **17** in the reaction to <15% (Table 1); however, we were unable to avoid the formation of **17** under any of the attempted reaction conditions (Scheme 3).

**Table 1:** Effect of solvent and additive on the ratio of **9** and **17**.

	Base	Solvent /Additives	Reaction time (h)	*α-*Ketoamide **9** (%)	Amide **17** (%)

1	TEA	DMSO/NaI	6	60	15
2	TEA	Sulfolane/NaI	16	52	15
3	TEA	DMSO	25	42	25

**Scheme 4:**
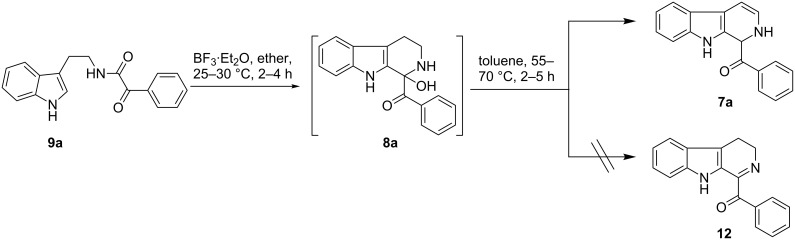
Synthesis of dihydroeudistomin Y analogues.

The *α*-ketoamide **9** thus obtained by the oxidative amidation methodology was subjected to a Bischler–Napieralski cyclodehydration reaction in presence of POCl_3_ [27], however under these conditions 2,9-dihydro-β-carboline derivative **7** was obtained as the major product (less than 10% yield) instead of 4-9-dihydro-β-carboline derivative **12** (Scheme 4). Our attempts to improve the yield of **7** under Bischler–Napieralski cyclodehydration reaction conditions using POCl_3_ as the Lewis acid were proved to be futile. This prompted us to test the efficiency of other Lewis acids in this cyclodehydration reaction. The Bischler–Napieralski cyclodehydration reaction was then carried out with different Lewis acids such as BF_3_·Et_2_O, SnCl_4_, TiCl_4_ etc., in mutiple solvents under various reaction conditions. The best conversion was obtained when the reaction was conducted in BF_3_·Et_2_O in ether at 25–30 °C and the product dihydro-β-carboline **7** was isolated in 55% of yield.

**Scheme 5:**
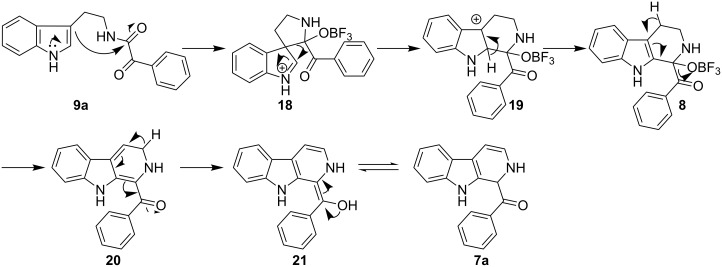
Plausible mechanism for the formation of **7**.

The formation of isomeric 2,9-dihydro-β-carboline derivative **7** as the major product in Bischler–Naperalski reaction conditions is explained in Scheme 5. The initially formed spirocyclic compound **18** undergoes intramolecular rearrangement and afforded the dihydrocarboline framework **19**, which on aromatization yielded **20**. Based on the reaction conditions **8** can be obtained from **20** after aqueous work-up or at higher temperature heating, **20** undergoes a sequential prototropic migration leading to the formation of 2,9-dihydro-β-carboline derivative **7.** To the best of our knowledge, this is a novel method for the synthesis of 1-benzoyldihydrocarboline from *α*-ketoamide **9** [28].

**Scheme 6:**
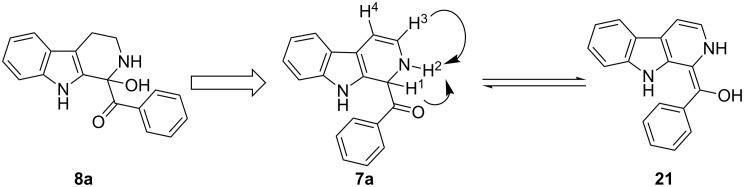
Rearrangement of **8a** into **7a** and coupling interactions of **7a**.

**Figure 2:**
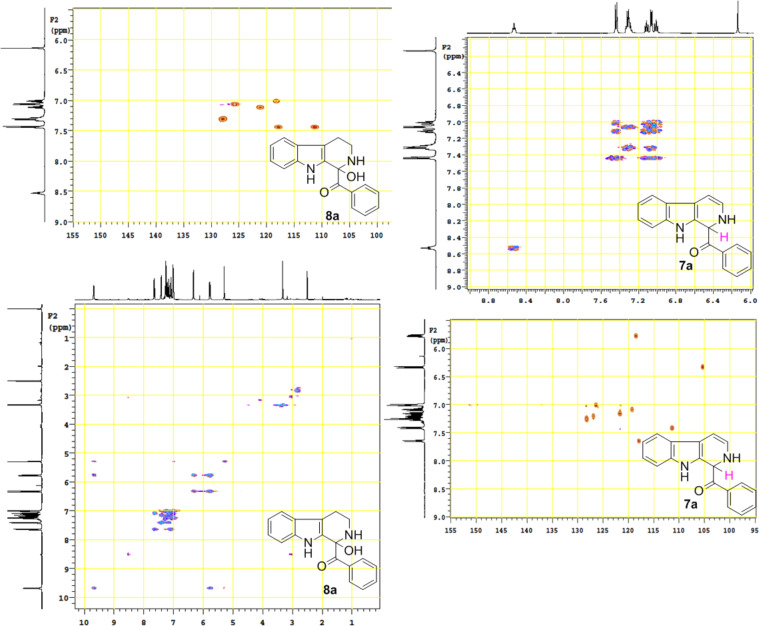
COSY and HSQC of **8a** and **7a**.

The structure of 2,9-dihydro-β-carboline **7** and 1-hydroxytetrahydro-β-carboline **8** was confirmed by various NMR techniques such as 2D NMR, COSY as well as HSQC (Figure 2). The coupling of the H^2^ proton with both H^1^ and H^3^ protons is clearly evident in the COSY for **7a**, whereas the OH proton in **8a** shows a high nOe (Scheme 6).

Dihydro-β-carboline **7** was then subjected to an aromatization reaction to obtain eudistomin Y (**6**). The aromatization reaction was attempted with oxidizing agents such as DDQ and MnO_2_ as per the reported conditions in literature. The formation of product **6** was confirmed by LCMS analysis of the crude reaction mixture. However under these conditions product **6** was observed in less than 5% and our attempt to isolate the eudistomin Y (**6**) in pure form was not successful. The oxidation of compound **7** with oxygen, KMnO_4_, MnO_2_ and TBHP as well as dehydrogenation with DBU in different solvents at various temperatures also failed and did not yield the required product. Attempted oxidation of compound **7** under microwave irradiation conditions was also not successful. Probably under all aforementioned conditions, formation of the stable enol **21** might have retarded the aromatization during the course of dehydrogenation reaction of dihydro-β-carboline **7**.

Utilizing the oxidative amidation Bischler–Napieralski reaction conditions we have synthesized a number of dihydro-β-carbolines (Table 2) in moderate to good yields. The structures of these compounds were confirmed by spectral and analytical methods.

**Table 2:** Synthesis of dihydro-*β*-carbolines^a^.

	Dibromo- ethanone **11**	*α*-Ketoamide **9**	Yield of **9** (%)	2,9-Dihydro- eudistomin **7**	Yield of **7** and **8** (%)	1-Hydroxy-4,9-tetra- hydroeudistomin **8**

1	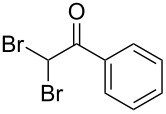 **11a**	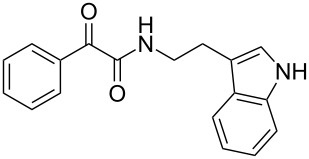 **9a**	60	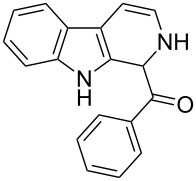 **7a**	40 (**7a**) 15 (**8a**)	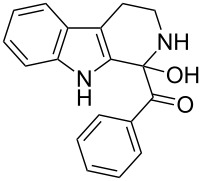 **8a**
2	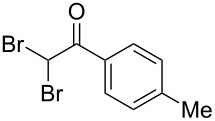 **11b**	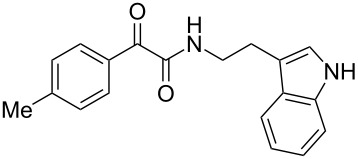 **9b**	57	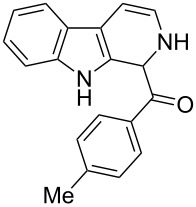 **7b**	48 (**7b**)	**8b** not isolated
3	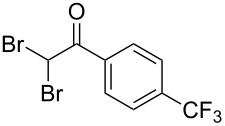 **11c**	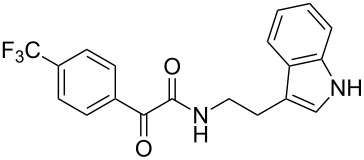 **9c**	52	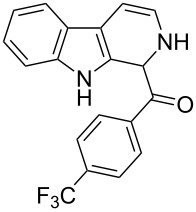 **7c**	38 (**7c**)	**8c** not isolated
4	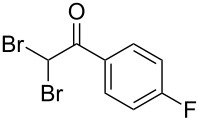 **11d**	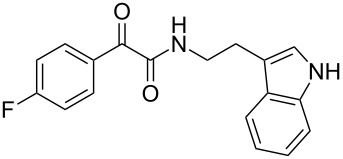 **9d**	45	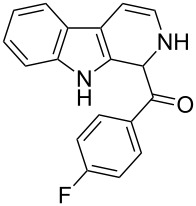 **7d**	25 (**7d**) 12 (**8d**)	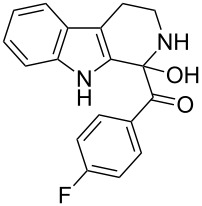 **8d**
5	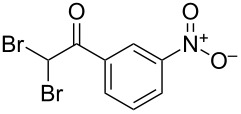 **11e**	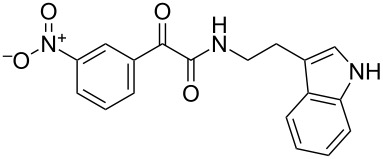 **9e**	38	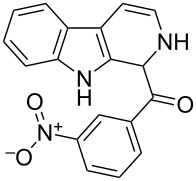 **7e**	20 (**7e**)	**8e** not isolated
6	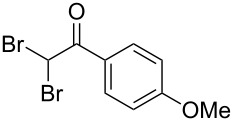 **11f**	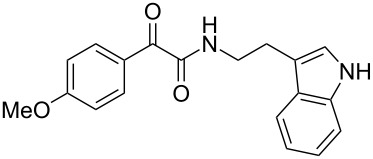 **9f**	55	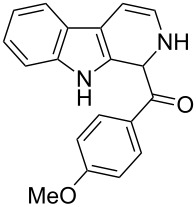 **7f**	38 (**7f**)	**8f** not isolated
7	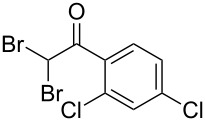 **11g**	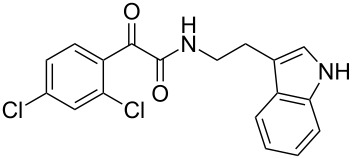 **9g**	50	**7g** not isolated	45 (**8g**)	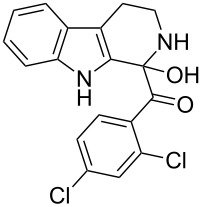 **8g**
8	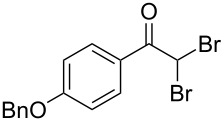 **11h**	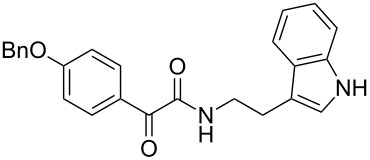 **9h**	52	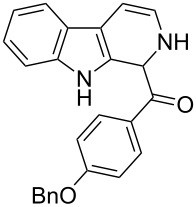 **7h**	46 (**7h**)	**8h** not isolated
9	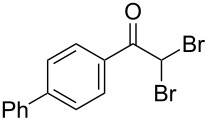 **11i**	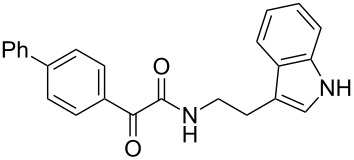 **9i**	60	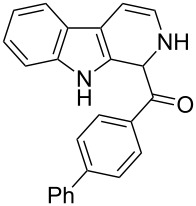 **7i**	35 (**7i**) 20 (**8i**)	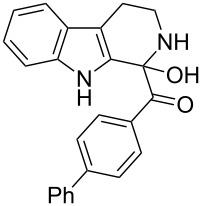 **8i**
10	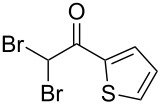 **11j**	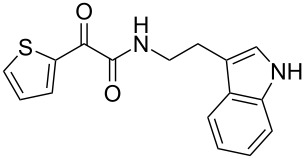 **9j**	42	**7j** not isolated	42 (**8j**)	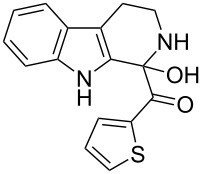 **8j**

^a^All products were characterized by ^1^H NMR, ^13^C NMR, Mass and IR.

## Conclusion

In conclusion, we have developed an oxidative amidation Bischler–Napieralski reaction methodology for the synthesis of dihydroeudistomin Y (**7a**–**7i**). A number of 1-benzoyl dihydro-β-carboline derivates have been synthesized as a part of these studies. The oxidative amidation Bischler–Napieralski reaction provides a simple and direct method for the synthesis of carbolines, which are otherwise synthesized by multistage reactions utilizing starting materials which are not readily available.

## Experimental

### General procedure for the synthesis of α-ketoamides (**9a**–**9j**)

A mixture of 2,2-dibromo-1-phenylethanone (**11a**, 6.0 g, 21.6 mmol) and sodium iodide (6.48 g, 43.2 mmol) in dimethyl sulfoxide (30.0 mL) at 25–45 °C was stirred for 40–50 minutes. Triethylamine (6.55 g, 64.8 mmol) and tryptamine (**10**, 3.45 g, 21.6 mmol) were then added to the mixture under a N_2_ atmosphere and it was stirred for 1–2 h at 25–45 °C. Then cumene hydroperoxide (88% *n*-hexane solution, 3.73 g, 21.6 mmol) was added to the mixture over a period of five minutes (exothermic reaction), and it was further stirred for another 3–6 h at the same temperature. After completion of the reaction (TLC), ice water was added to the mixture and extracted with DCM (2 × 50 mL). The DCM layer was washed with aq sodium bisulfite solution (50 mL) followed by brine (50 mL) and H_2_O (2 × 60 mL). The organic layer was dried (anhyd. Na_2_SO_4_) and evaporated to dryness under reduced pressure. The obtained crude product was subjected to CC purification and afforded the desired product in moderate to good yields.

***N*-(2-(1*H*-Indol-3-yl)ethyl)-2-oxo-2-phenylacetamide (9a):** 60% yield; mp 135–136.7 °C; IR (cm^–1^): 3408, 3386, 2927, 2862, 1663, 1531, 1288, 1248, 1218, 1093, 1080, 1071, 838, 742; ^1^H NMR (400 MHz, CDCl_3_) δ_H_ 8.32 (d, *J* = 8 Hz, 2H), 8.05 (s, 1H, NH), 7.63 (dd, *J* = 8.4, 6.8 Hz, 2H), 7.47 (t, *J* = 8 Hz, 2H), 7.38 (d, *J* = 8 Hz, 1H), 7.22 (dd, *J* = 6.0, 7.2 Hz, 1H), 7.15 (dd, *J* = 1.2, 6.8 Hz, 1H), 7.12 (dd, *J* = 2.0, 8.8 Hz, 1H), 7.10 (s, 1H), 3.75 (q, *J* = 6.8 Hz, 2H), 3.09 (t, *J* = 6.8 Hz, 2H); ^13^C NMR (100 MHz, DMSO-*d*_6_) δ_c_ 190.4, 164.8, 136.1, 134.3, 132.8, 129.6 (2C), 128.7 (2C), 127.1, 122.8, 120.8, 118.2 (2C), 111.2 (2C), 40.7, 24.7; MS *m*/*z* (%): 293 (M + 1), 315 (M + 23).

***N*-(2-(1*H*-Indol-3-yl)ethyl)-2-oxo-2-(*p*-tolyl)acetamide (9b):** 57% yield; mp 116.8–118.1 °C; IR (cm^–1^): 3340, 3255, 3093, 2841, 1661, 1644, 1514, 1457, 1310, 1263, 1224, 1169, 1024, 849, 798, 742; ^1^H NMR (400 MHz, CDCl_3_) δ_H_ 8.24 (d, *J* = 8 Hz, 2H), 8.06 (s, 1H, NH), 7.64 (d, *J* = 8 Hz, 1H), 7.39 (d, *J* = 8 Hz, 1H), 7.21 (t, *J* = 7.2 Hz, 2H), 7.13 (t, *J* = 7.2 Hz, 2H), 7.07 (d, *J* = 2.4 Hz, 1H), 3.73 (d, *J* = 6.4 Hz, 2H), 3.07 (t, *J* = 6.8 Hz, 2H), 2.42 (s, 3H): ^13^C NMR (100 MHz, DMSO-*d*_6_) δ_c_ 190.1, 165.2, 145.2, 136.2, 132.3, 129.8, 129.4, 127.1, 125.7, 122.8, 120.9 118.3 (2C), 114.2, 111.3 (2C), 55.7, 24.8, 21.4; MS *m*/*z* (%): 307.5 (M + 1), 329.5 (M + 23).

***N*-(2-(1*H*-Indol-3-yl)ethyl)-2-oxo-2-(4-(trifluoromethyl)phenyl)acetamide (9c):** 52% yield; mp 139.8–140.1 °C; IR (cm^−1^): 3338, 2938, 2842, 1640, 1334, 1224, 1157, 1135, 1075, 935, 842, 794, 747; ^1^H NMR (400 MHz, CDCl_3_) δ_H_ 8.42 (d, *J* = 8 Hz, 2H), 8.06 (s, 1H, NH), 7.73 (d, *J* = 8 Hz, 2H), 7.64 (d, *J* = 8 Hz, 1H), 7.40 (d, *J* = 8 Hz, 1H), 7.22 (t, *J* = 7.2 Hz, 1H), 7.14 (t, *J* = 7.2 Hz, 2H), 3.75 (d, *J* = 6.8 Hz, 2H), 3.09 (t, *J* = 6.8 Hz, 2H); ^13^C NMR (100 MHz, DMSO-*d*_6_) δ_c_ 189.0, 163.7, 136.1 (2C), 130.4 (2C), 127.1, 125.6 (2C), 122.8, 120.8 118.2 (2C), 111.2 (2C), 39.2, 24.7; MS *m*/*z* (%): 383 (M + 1), 405 (M + 23).

***N*-(2-(1*H*-Indol-3-yl)ethyl)-2-(4-fluorophenyl)-2-oxoacetamide (9d):** 45% yield; mp 138.5–141.2 °C; IR (cm^−1^): 3338, 3335, 2923, 2858, 1682, 1651, 1595, 1522, 1352, 1273, 1228, 1152, 850, 798, 742; ^1^H NMR (400 MHz, CDCl_3_) δ_H_ 8.41 (dd, *J* = 5.2, 2 Hz, 2H), 8.09 (s, 1H, NH), 7.63 (d, *J* = 7.6 Hz, 1H), 7.38 (d, *J* = 8 Hz, 1H), 7.21 (t, *J* = 7.2 Hz, 1H), 7.14 (t, *J* = 2 Hz, 1H), 7.13 to 7.11 (m, 1H), 7.10 (t, *J* = 2 Hz, 1H), 7.06 (d, *J* = 1.6 Hz, 1H), 3.73 (d, *J* = 6.4 Hz, 2H), 3.07 (t, *J* = 6.8 Hz, 2H); ^13^C NMR (100 MHz, CDCl_3_) δ_c_ 188.6, 164.3, 163.0, 136.1, 132.9, 132.7, 129.6, 127.1, 122.8, 120.9, 116.1, 115.7, 118.2 (2C), 111.2 (2C), 39.2, 24.7; MS *m*/*z* (%): 311.5 (M + 1), 333.4 (M + 23).

***N*-(2-(1*H*-Indol-3-yl)ethyl)-2-(3-nitrophenyl)-2-oxoacetamide (9e):** 38% yield; mp 138.1–139.5 °C; IR (cm^−1^): 3364, 3331, 3121, 2860, 1662, 1523, 1346, 1263, 1212, 1098, 815, 752, 725; ^1^H NMR (400 MHz, CDCl_3_) δ_H_ 9.13 (s, 1H), 8.68 (d, *J* = 7.2 Hz, 1H), 8.46 (d, *J* = 7.2 Hz, 2H), 8.09 (s, 1H, NH), 7.67 (dd, *J* = 8 Hz, 1H), 7.40 (d, *J* = 8 Hz, 1H), 7.25 to 7.10 (m, 3H, ArH), 3.77 (d, *J* = 6.6 Hz, 2H), 3.10 (t, *J* = 7 Hz, 2H); ^13^C NMR (100 MHz, DMSO-*d*_6_) δ_c_ 187.3, 162.8, 147.7, 136.1, 135.8, 134.1, 130.4, 128.2, 127.2, 124.2, 122.7, 120.8, 118.2, 111.2, 39.2, 24.7; MS *m*/*z* (%): 338.5 (M^+^), 360.5 (M^+^, 23).

### General procedure for the synthesis of dihydroeudistomin (**7a**–**7j**)

To a stirred solution of *N*-(2-(1*H*-indol-3-yl)ethyl)-2-oxo-2-phenylacetamide (**9a**, 2.0 g, 6.8 mmol) in diethyl ether (20 mL) was added 48% boron trifluoride etherate (10.1 g, 34.2 mmol) in a round-bottom flask under a N_2_ atmosphere, and the mixture was stirred at 25–30 °C for 2 h. After TLC indication for absence of starting material toluene (20 mL) was added. Then mixture was heated to 55–70 °C and it was maintained under stirring at that temperature for 4–8 h (TLC). The mixture was cooled to 25–30 °C and it was poured into a pre-cooled sat. NaHCO_3_ solution (160 mL) at 10 °C. The product was then extracted with ethyl acetate (2 × 25 mL) and the organic layer was washed with 10% NaHCO_3_ solution (25 mL) and dried (anhyd. Na_2_SO_4_). The solvent was distilled off under reduced pressure and the crude residue was subjected to column chromatography purification. The product was obtained in good to moderate yields.

**(2,9-Dihydro-1*H*-pyrido[3,4-*b*]indol-1-yl)(phenyl)methanone (7a):** 40% yield; mp 228.5–230.6 °C; ^1^H NMR (400 MHz, DMSO-*d*_6_) δ_H_ 11.37 (br s, 1H, NH), 9.68 (d, *J* = 5.2 Hz, 1H), 7.65 (d, *J* = 8.0 Hz, 1H), 7.42 (d, *J* = 7.6 Hz, 1H), 7.26 (t, *J* = 7.4 Hz, 2H), 7.22 (d, *J* = 7.2 Hz, 1H), 7.16 (t, *J* = 7.6 Hz, 1H), 7.08 (t, *J* =7.2 Hz, 1H), 7.01 (d, *J* = 7.2 Hz, 2H), 6.34 (d, *J* = 8.8 Hz, 1H), 5.77 (dd, *J* = 3.2 & 5.6 Hz, 1H), 5.29 (s, 1H); ^13^C NMR (100 MHz, DMSO-*d*_6_) δ_c_ 167.1, 136.4, 136.1, 130.8, 128.5 (2C), 127.2, 126.6 (2C), 125.9, 122.0, 119.6, 118.7, 118.2, 111.6, 109.9, 105.7, 53.2; MS *m*/*z* (%): 275.1 (M + 1), 297.2 (M + 23); HRMS (EI) *m*/*z*: [M]^+^ calcd for C_18_H_15_N_2_O, 275.1184; found, 275.1173.

**(1-Hydroxy-2,3,4,9-tetrahydro-1*H*-pyrido[3,4-*b*]indol-1-yl)(phenyl)methanone (8a):** 15% yield; mp 138.5–141.2 °C; ^1^H NMR (400 MHz, DMSO-*d*_6_) δ_H_ 10.93 (s, 1H), 8.52 (t, *J* = 5.2 Hz, 1H, NH), 7.44 (d, *J* = 6.8 Hz, 2H), 7.33 to 7.26 (m, 3H, ArH), 7.11 (t, *J* = 6.0 Hz, 1H), 7.06 (d, *J* = 5.6 Hz, 2H), 7.00 (t, *J* = 6.0 Hz, 1H), 6.13 (s, 1H, D_2_O exchangable proton), 3.10–3.02 (m, 2H), 2.88–2.49 (m, 2H); ^13^C NMR (100 MHz, DMSO–*d*_6_) δ_c_ 173.9, 143.9, 135.0, 131.0, 128.3 (2C), 127.8, 127.8, 126.0 (2C), 121.3, 118.5, 117.9, 111.5, 108.4, 75.6 (alkoxy carbon), 38.3, 25.2. HRMS (EI) *m*/*z*: [M]^+^ calcd for C_18_H_17_N_2_O_2_, 293.1290; found, 293.1333; HRMS of dehydrated **8a** (EI) *m*/*z*: [M]^+^ calcd for C_18_H_15_N_2_O, 275.1184; found, 275.1220.

**(2,9-Dihydro-1*H*-pyrido[3,4-*b*]indol-1-yl)(*p*-tolyl)methanone (7b):** 48% yield; mp 213.2–215.7 °C; IR (cm^−1^): 3222, 3179, 3043, 2940, 1656, 1625, 1558, 1479, 1335, 1246, 1065, 802, 768, 739; ^1^H NMR (400 MHz, DMSO-*d*_6_) δ_H_ 11.36 (br s, 1H, NH), 9.64 (d, *J* = 4.8 Hz, 1H), 7.64 (d, *J* = 7.6 Hz, 1H), 7.41 (d, *J* = 8.0 Hz, 1H), 7.15 (t, *J* = 7.6 Hz, 1H), 7.10 (d, *J* = 7.6 Hz, 1H), 7.06 (d, *J* = 7.6 Hz, 2H), 6.89 (d, *J* = 8.0 Hz, 2H), 6.32 (d, *J* = 8.4 Hz, 1H), 5.76 (dd, *J* = 3.6 & 5.6 Hz, 1H), 5.23 (s, 1H), 2.20 (s, 3H); ^13^C NMR (100 MHz, CDCl_3_ + DMSO-*d*_6_) δ_c_ 166.3, 135.1, 134.9, 131.5, 129.7, 127.5 (2H), 125.4 (2C), 124.6, 120.3, 118.1, 117.1, 116.5, 110.1, 108.5, 104.5, 51.6, 19.5; MS *m/z* (%): 289.5 (M + 1), 311.5 (M + 23); HRMS (EI) *m*/*z*: [M]^+^ calcd for C_19_H_17_N_2_O, 289.1341; found, 289.1329.

**(2,9-Dihydro-1*H*-pyrido[3,4-*b*]indol-1-yl)(4-(trifluoromethyl)phenyl)methanone (7c):** 38% yield. mp 162–164 °C; IR (cm^−1^): 3222, 3185, 3042, 2937, 2835, 1657, 1624, 1509, 1456, 1249, 1177, 1055, 1032, 848, 810, 770, 742; ^1^H NMR (400 MHz, CDCl_3_) δ_H_ 8.10 (s, 1H), 7.69 (d, *J* = 7.6 Hz, 1H), 7.58 (d, *J* = 8.0 Hz, 2H), 7.38 (t, *J* = 8.0 Hz, 2H), 7.29–7.21 (m, 4H), 6.48 (d, *J* = 8.8 Hz, 1H), 5.94 (dd, *J* = 3.6 & 5.6 Hz, 1H), 5.19 (s, 1H); ^13^C NMR (100 MHz, DMSO-*d*_6_) δ_c_ 166.0, 140.5, 136.3, 129.9, 127.9, 127.6, 127.4 (2C), 125.6 (doublet), 125.3 (2C), 121.9, 119.5, 118.7, 118.1, 111.6, 109.9, 105.7, 52.7; MS *m*/*z* (%): 343 (M + 1); HRMS (EI) *m*/*z*: [M]^+^ calcd for C_19_H_14_N_2_OF_3_, 343.1058; found, 343.1038.

**(2,9-dihydro-1*H*-pyrido[3,4-*b*]indol-1-yl)(4-fluorophenyl)methanone (7d):** 25% yield; mp 230.6–232.2 °C; IR (cm^−1^): 3351, 3279, 3083, 2895, 2839, 1659, 1605, 1508, 1459, 1304, 1234, 1161, 1072, 956, 837, 747; ^1^H NMR (400 MHz, CDCl_3_) δ_H_ 8.12 (s, 1H), 7.68 (d, *J* = 7.6 Hz, 1H), 7.37 (d, *J* = 7.6 Hz, 1H), 7.27–7.19 (m, 4H, ArH), 7.00 (t, *J* = 8.8 Hz, 2H), 6.51 (d, *J* = 8.4 Hz, 1H), 5.94 (dd, *J* = 3.6 & 4.8 Hz, 1H), 5.08 (s, 1H); ^13^C NMR (100 MHz, DMSO-*d*_6_) δ_c_ 166.7, 162.4, 136.3, 132.1, 132.1, 130.6 (doublet), 128.6 (2C), 128.6, 125.7, 121.9, 119.5, 118.7, 118.1, 115.3, 115.1, 111.6, 109.8, 105.6, 52.3; MS *m*/*z* (%): 293 (M + 1); HRMS (EI) *m*/*z*: [M]^+^ calcd for C_18_H_14_N_2_OF, 293.1090; found, 293.1073.

**(4-Fluorophenyl)(1-hydroxy-2,3,4,9-tetrahydro-1*H*-pyrido[3,4-*b*]indol-1-yl)methanone (8d):** 12% yield; mp 185–187 °C; IR (cm^−1^): 3268, 3178, 3041, 2934, 1658, 1626, 1506, 1471, 1335, 1239, 1225, 1156, 1014, 976, 840, 816, 738; ^1^H NMR (400 MHz, CDCl_3_ + DMSO-*d*_6_) δ_H_ 9.65 (s, 1H), 7.89 (t, *J* = 6.8 Hz, 1H), 7.47 (t, *J* = 9.4 Hz, 3H), 7.22–7.08 (m, H, ArH), 6.96 (t, *J* = 8.8 Hz, 2H), 5.92 (s, 1H), 3.24–3.19 (m, 2H), 2.94 (m, 2H); ^13^C NMR (100 MHz, DMSO-*d*_6_) δ_c_ 173.6, 140.3, 135.0, 130.8, 128.2, 128.1, 127.7, 121.4, 118.5, 118.0, 115.3, 114.9, 111.5, 108.5, 75.0, 25.1; MS *m*/*z* (%): 293 (M + 1), 315 (M + 23); HRMS (EI) *m*/*z*: [M]^+^ calcd for C_18_H_14_N_2_OF, 293.1090; found, 293.1070.

## Supporting Information

File 1 Experimental and analytical data.
