# Supplementary material for: An oxidative amidation and heterocyclization approach for the synthesis of β-carbolines and dihydroeudistomin Y
Source: Beilstein J Org Chem. 2014 Feb 25;10:471–80. doi: 10.3762/bjoc.10.45 (PMC3943975; doi:10.3762/bjoc.10.45)

Supporting Information

for

An oxidative amidation and heterocyclization approach for the synthesis of β-carbolines and dihydroeudistomin Y

Suresh Babu Meruva*[1, 2], Akula Raghunadh[1], Raghavendra Rao Kamaraju[1], U. K. Syam Kumar[1] and P. K. Dubey[2]

Address: [1]Technology Development Centre, Custom Pharmaceutical Services, Dr. Reddy's Laboratories Ltd., Miyapur, Hyderabad - 500049, India and [2]Department of Chemistry, College of Engineering, JNTUH, Kukatpally, Hyderabad - 500085, India.

Email: Suresh Babu Meruva* - sureshbabum@drreddys.com

*Corresponding author

Experimental and analytical data

Table of contents

Section A: General information..s2

Section B: Experimental section...s2

Section C: [1]H, ^{13}C, Mass, and IR...s7

Section A: General information

All reagents were used as received from commercial sources without further purification or prepared as described in the literature. Reactions were stirred using Teflon-coated magnetic stirring bars. TLC plates were visualized by ultraviolet light or by treatment with a spray of Pancaldi reagent {$(NH4)_6MoO_4$, $Ce(SO_4)_2$, H_2SO_4, H_2O}. Chromatographic purification of products was carried out by flash column chromatography on silica gel (60–120mesh). Melting points were determined either on DSC-60A, Schimadzu or on an electro thermal melting point apparatus and are uncorrected. NMR spectra were measured in $CDCl_3$, and DMSO-d_6 (all with TMS as internal standard) on a Varian Gemini 400 MHz FT and 500 MHz FT magnetic resonance spectrometers. Chemical shifts (*δ*) are reported in ppm, and coupling constants (*J*) are in Hz. The following abbreviations were used to explain the multiplicities: s = singlet, d = doublet, t = triplet, q = quartet, m = multiplet. Mass spectra were recorded on an HP-5989A quadrapole mass spectrometer.

Section B: Experimental section:

Analytical data of *α*-keto-amides (9f–9j).

***N*-(2-(1*H*-Indol-3-yl)ethyl)-2-(4-methoxyphenyl)-2-oxoacetamide (9f):**

55% Yield mp 130-132.4 °C. IR (cm^{-1}): 3246, 2935, 2842, 1668, 1650, 1621, 1598, 1568, 1315, 1264, 1173, 1029, 841, 764, 741. ^1H NMR (400 MHz, $CDCl_3$): δ_H 8.40 (d, 2H, *J* = 9.2 Hz), 8.04 (1H, s, NH), 7.65 (d, 1H, *J* = 7.6 Hz), 7.39 (d, 1H, *J* = 8 Hz), 7.23 to 7.09 (3H ArH), 6.94 (d, 2H, *J* = 8.8 Hz), 3.88 (s, 3H), 3.73 (d, 2H, *J* = 6.8 Hz), 3.09 (t, 2H, *J* = 6.8 Hz). ^{13}C NMR (100 MHz, DMSO d^6): δ_c 188.7, 165.0, 164.0, 136.1, 132.2

(2C), 127.1, 125.7, 122.7, 120.8, 118.2 (2C), 114.1 (2C), 111.3 (2C), 55.6, 39.2 and 24.7. Ms, *m/z* (%) = 323.2 (M+1), 345.2 (M+23).

***N*-(2-(1*H*-Indol-3-yl)ethyl)-2-(2,4-dichlorophenyl)-2-oxoacetamide (9g):**

50% Yield. ^{1}H NMR (400 MHz, DMSO d^6): δ_H 10.84 (s, 1H, NH), 9.14 (s, 1H, NH), 7.77 (s, 1H), 7.62 to 7.53 (3H, ArH), 7.36 (d, 1H, *J* = 2.0 Hz), 7.19 (s, 1H), 6.98 (dd, 1H, *J* = 7.6 Hz), 7.09 (dd, 1H, *J* = 7.6 Hz), 3.52 (d, 2H, *J* = 6.8 Hz), 2.95 (t, 2H, *J* = 7.2 Hz). ^{13}C NMR (100 MHz, DMSO d^6): δ_c 189.5, 162.3, 137.5, 136.2, 132.8, 132.6, 129.8 (2C), 127.4, 127.1, 122.7, 120.9, 118.2 (2C), 111.3 (2C), 55.7 and 24.5. Ms, *m/z* (%) = 361.1 (M+1), 363.1 (M+3), 383.1 (M+23).

***N*-(2-(1*H*-Indol-3-yl)ethyl)-2-(4-(benzyloxy)phenyl)-2-oxoacetamide (9h)**:

52% Yield. mp 137.1-138.2 °C. IR (cm^{-1}): 3439, 3251, 3091, 2902, 1681, 1643, 1598, 1568, 1456, 1258, 1225, 1173, 995, 850, 741. ^{1}H NMR (400 MHz, $CDCl_3$): δ_H 8.39 (d, 2H, *J* = 9.2 Hz), 8.06 (s, 1H, NH), 7.64 (d, 1H, *J* = 7.6 Hz), 7.43- 7.34 (m, 5 ArH), 7.23 – 7.13 (m, 3ArH), 7.11 (s, 1H), 7.02 (d, 2H, *J* = 9.2 Hz), 5.25 (s, 2H), 3.72 (d, 2H, *J* = 6.6 Hz), 3.07 (t, 2H, *J* = 6.8 Hz). ^{13}C NMR (100 MHz, DMSO d^6): δ_c 188.6, 164.9, 163.0, 136.1 (2C), 132.1 (2C), 128.3 (2C), 127.9, 127.6 (2C), 127.0, 125.9, 122.6, 120.8, 118.1 (2C), 114.8 (2C), 111.3, 111.2, 69.5, 39.2 and 24.6. Ms, *m/z* (%) = 399.4 (M+1).

***N*-(2-(1*H*-Indol-3-yl)ethyl)-2-([1,1'-biphenyl]-4-yl)-2-oxoacetamide (9i)**:

60% Yield. mp 175.1-177.1 °C. IR (cm^{-1}): 3436, 3239, 3089, 1684, 1645, 1599, 1485, 1457, 1202, 1100, 857, 762, 743. ^{1}H NMR: (400 MHz, $CDCl_3$): δ_H 8.41 (d, 2H, *J* = 6.6 Hz), 8.06 (s, 1H, NH), 7.70 - 7.63 (m, 5 ArH), 7.47- 7.38 (m, 4 ArH), 7.22 – 7.10 (m, 3 ArH), 3.76 (d, 2H, *J* = 6.6 Hz), 3.10 (t, 2H, *J* = 6.8 Hz). ^{13}C NMR (100 MHz, DMSO d^6):

δ_c 189.9, 164.8, 145.6, 138.6, 136.2, 131.6, 130.4 (2C), 129.1 (2C), 128.6, 127.1 (2C), 127.0 (2C), 126.9, 122.9, 120.9, 118.2 (2C), 111.3 (2C), 39.2 and 24.8. Ms, *m/z* (%) = 369.2 (M+1), 391.2 (M+23).

N-(2-(1*H*-Indol-3-yl)ethyl)-2-oxo-2-(thiophen-2-yl)acetamide (9j):

42% Yield. mp 147.7-149.9 °C. IR (cm^{-1}): 3394, 3354, 2936, 2916, 2859, 1685, 1654, 1567, 1500, 1404, 1357, 1287, 1093, 1023, 844, 817, 743, 725. ^{1}H NMR (400 MHz, $CDCl_3$): δ_H 8.38 (d, 1H, *J* = 4 Hz), 8.08 (s, 1H, NH), 7.81 (d, 1H, *J* = 5.5 Hz), 7.63 (d, 1H, *J* = 8.0 Hz), 7.39 (d, 2H, *J* = 8.4 Hz), 7.23 – 7.11 (m, 2 ArH), 7.06 (d, 1H, *J* = 2 Hz), 3.73 (d, 2H, *J* = 6.6 Hz), 3.07 (t, 2H, *J* = 7.2 Hz). ^{13}C NMR (100 MHz, DMSO d^6): δ_c 179.8, 161.8, 138.8, 137.2, 137.2, 136.2, 128.5, 127.1, 122.6, 120.9, 118.2 (2C), 111.3 (2C), 39.2 and 24.6. Ms, *m/z* (%) = 299.4 (M+1), 321.4 (M+23).

Analytical data of dihydro-eudistomin (7e–7j).

(2,9-Dihydro-1*H*-pyrido[3,4-*b*]indol-1-yl)(3-nitrophenyl)methanone (7e):

20% Yield. ^{1}H NMR (400 MHz, $CDCl_3$): δ_H 8.33 (s, 1H), 8.14 (d, 1H, *J* = 8.0 Hz), 8.06 (s, 1H), 7.68 (d, 1H, *J* = 8.0 Hz), 7.53 (d, 1H, *J* = Hz), 7.48 (t, 1H, *J* = 8.0 Hz), 7.41 (d, 1H, *J* = 8.0 Hz), 7.30 - 7.17 (m, 3 ArH), 6.52 (d, 1H, *J* = 8.4 Hz), 5.91 (dd, 1H, *J* = 3.2 & 5.4 Hz), 5.28 (s, 1H), 3.78 (s, 3H). Ms, *m/z* (%) = 320 (M+1).

(2,9-Dihydro-1*H*-pyrido[3,4-*b*]indol-1-yl)(4-methoxyphenyl)methanone (7f):

38% Yield. IR (cm^{-1}): 3222, 3185, 3042, 2937, 2835, 1657, 1624, 1509, 1456, 1249, 1177, 1055, 1032, 848, 810, 770, 742. ^{1}H NMR (400 MHz, $CDCl_3$): δ_H 8.17 (s, 1H), 7.67 (d, 1H, *J* = 7.2 Hz), 7.35 (t, 2H, *J* = 8.0 Hz), 7.23 (d, 1H, *J* = 7.2 Hz), 7.20 (d, 3H, *J* = 8.0 Hz), 6.86 (d, 2H, *J* = 7.6 Hz), 6.48 (d, 1H, *J* = 8.8 Hz), 5.94 (dd, 1H, *J* = 3.6 & 5.6 Hz), 5.02 (s, 1H), 3.78 (s, 3H). ^{13}C NMR (100 MHz, $CDCl_3$ + DMSO d^6): δ_c 166.8, 157.3,

135.4, 130.1, 129.4, 127.0, 126.7, 124.9, 120.6, 118.4, 117.3, 116.9, 112.5, 110.6, 109.0, 108.8, 105.0, 53.9, 51.5. Ms, *m/z* (%) = 305 (M+1), 327 (M+23).

(2,4-Dichlorophenyl)(1-hydroxy-2,3,4,9-tetrahydro-1*H*-pyrido[3,4-*b*]indol-1-yl)methanone (8g):

45% Yield. mp 281.1-284.2 °C. IR (cm^{-1}): 3356, 3211, 3086, 2834, 1678, 1581, 1556, 1469, 1385, 1337, 1184, 1110, 1073, 1052, 906, 822, 800, 745. ^{1}H NMR (400 MHz, DMSO d^6): δ_H 10.95 (s, 1H), 8.50 (t, 1H, *J* = 6.8 Hz), 7.64 (s, 1H), 7.43 (d, 2H, *J* = 8.4 Hz), 7.34 (dd, 1H, *J* = 2.0, 6.4 Hz) 7.12 (t, 1H, *J* = 7.6 Hz), 7.02 (t, 1H, *J* = 7.6 Hz), 6.77 (d, 1H, *J* = 8.8 Hz), 6.15 (s, 1H), 3.20 (m, 1H), 3.01 (m, 1H), 2.81 (m, 2H). ^{13}C NMR (100 MHz, DMSO d^6): δ_c 171.9, 139.7, 135.1, 133.4, 132.4, 131.6, 130.5, 130.2, 127.9, 127.1, 121.7, 118.7, 118.1, 111.7, 108.6, 74.8, 38.2 and 25.2. Ms, *m/z* (%) = 343.1 (M+1), 321.4 (M+23). HRMS (EI): calcd. *m/z* for $C_{18}H_{13}N_2OCl_2$ $[M]^+$ 343.0405, found 343.0101.

(4-(Benzyloxy)phenyl)(2,9-dihydro-1*H*-pyrido[3,4-*b*]indol-1-yl)methanone (7h):

46% Yield. ^{1}H NMR (400 MHz, $CDCl_3$): δ_H 8.04 (s, 1H), 7.65 (d, 1H, *J* = 8.8 Hz), 7. 40-7.32 (m, 5 ArH), 7.23-7.11 (m, 4 ArH), 6.95 (d, 2H, *J* = 8.8 Hz), 6.51 (d, 1H, *J* = 8.4 Hz), 5.95 (dd, 1H, *J* = 5.6 & 3.6 Hz), 5.04 (s, 2H), 5.00 (s, 1H). Ms, *m/z* (%) = 381.2 (M+1), 403 (M+23). HRMS (EI): calcd. *m/z* for $C_{25}H_{21}N_2O_2$ $[M]^+$ 381.1603, found 381.1583.

[1,1'-Biphenyl]-4-yl(2,9-dihydro-1*H*-pyrido[3,4-*b*]indol-1-yl)methanone (7i):

35% Yield. mp 224-226 °C. ^{1}H NMR (400 MHz, $CDCl_3$): δ_H 8.12 (s, 1H), 7.69 (d, 1H, *J* = 8.0 Hz), 7.64 - 7.21 (m, 12 ArH), 6.54 (d, 1H, *J* = 8.4 Hz), 5.97 (dd, 1H, *J* = 3.6 & 4.8 Hz), 5.13 (s, 1H). ^{13}C NMR (100 MHz, DMSO d^6): δ_c 173.7, 166.8, 143.0, 139.5, 138.9,

136.2, 135.2, 130.7, 128.8, 127.8, 127.3, 126.9, 126.6, 125.7, 121.8, 119.4, 118.6, 118.0, 111.5, 109.7, 108.5, 105.4, 75.4, 52.8, 25.2 Ms, *m/z* (%) = 351.2 (M+1).

[1,1'-Biphenyl]-4-yl(1-hydroxy-2,3,4,9-tetrahydro-1*H*-pyrido[3,4-*b*]indol-1-yl)methanone (8i):

20% Yield. mp 271.1-273.6 °C. ^{1}H NMR (400 MHz, $CDCl_3$): δ_H 8.81 (*s*, 1H), 7.55 - 7.27 (m, 11 ArH), 6.31 (t, 1H, *J* = 7.0 Hz, NH), 5.77 (s, 1H, OH), 3.49 (m, 1H), 3.27 (m, 1H), 3.00 (m, 2H). ^{13}C NMR (100 MHz, DMSO d^6): δ_c. 173.7, 143.0, 139.5, 139.4, 135.0, 130.9, 128.8 (3C), 127.7, 127.5, 126.6 (5), 121.3, 118.4, 117.9, 111.5, 108.5, 75.3,38.2 and 25.2. Ms, *m/z* (%) = 351.2 (M+1).

(1-Hydroxy-2,3,4,9-tetrahydro-1*H*-pyrido[3,4-*b*]indol-1-yl)(thiophen-2-yl)methanone (8j):

42% Yield. ^{1}H NMR (400 MHz, $CDCl_3$): δ_H 8.77 (s, 1H), 7.52 (d, 1H, *J* = 8.0 Hz), 7.44 (m, 2H), 7.17 (t, 2H, *J* = 7.6 Hz), 7.05 (d, 1H, *J* = 8.8 Hz), 6.84 (d, 1H, *J* = 8.8 Hz), 6.15 (brs, 1H, NH), 5.84 (s, 1H, OH), 3.36 – 3.29 (m, 2H), 3.02 (m, 1H), 2.89 (m, 1H). ^{13}C NMR (100 MHz, DMSO d^6): δ_c 171.8, 139.6, 135.1, 133.4, 132.4, 131.5, 130.5, 127.8, 127.0, 121.6, 118.0, 111.6, 108.6, 74.7, 38.2 and 25.1. Ms, *m/z* (%) = 281 (M+1).

Section C: ^{1}H, ^{13}C, Mass, and IR.

^{1}H NMR of *N*-(2-(1*H*-indol-3-yl)ethyl)-2-oxo-2-phenylacetamide (**9a**):

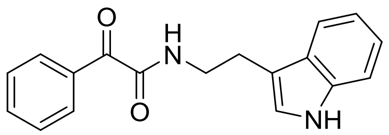

9a

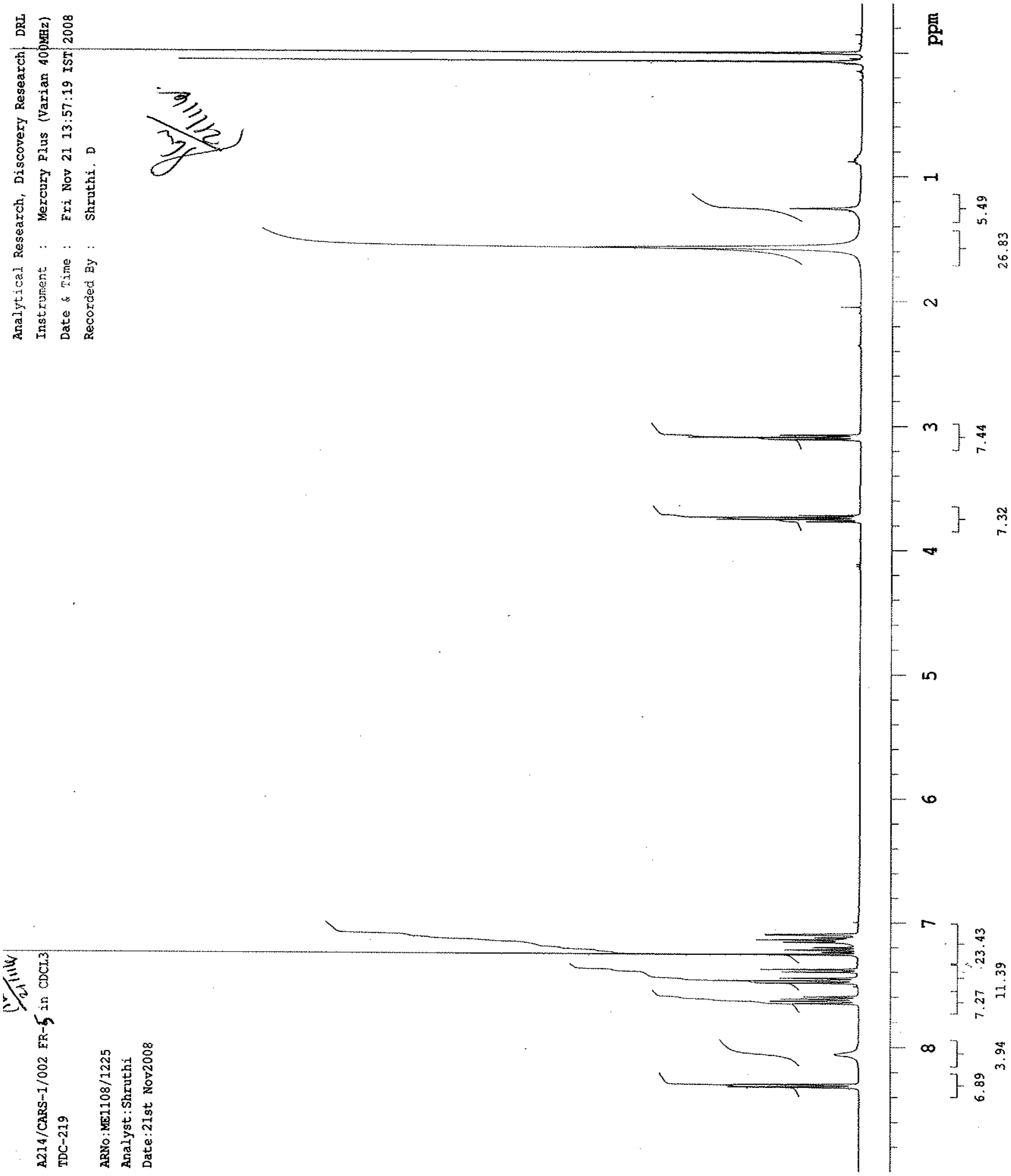

^{13}C NMR of *N*-(2-(1*H*-indol-3-yl)ethyl)-2-oxo-2-phenylacetamide (**9a**):

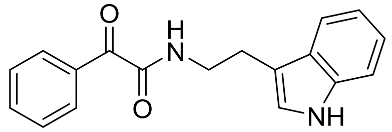

9a

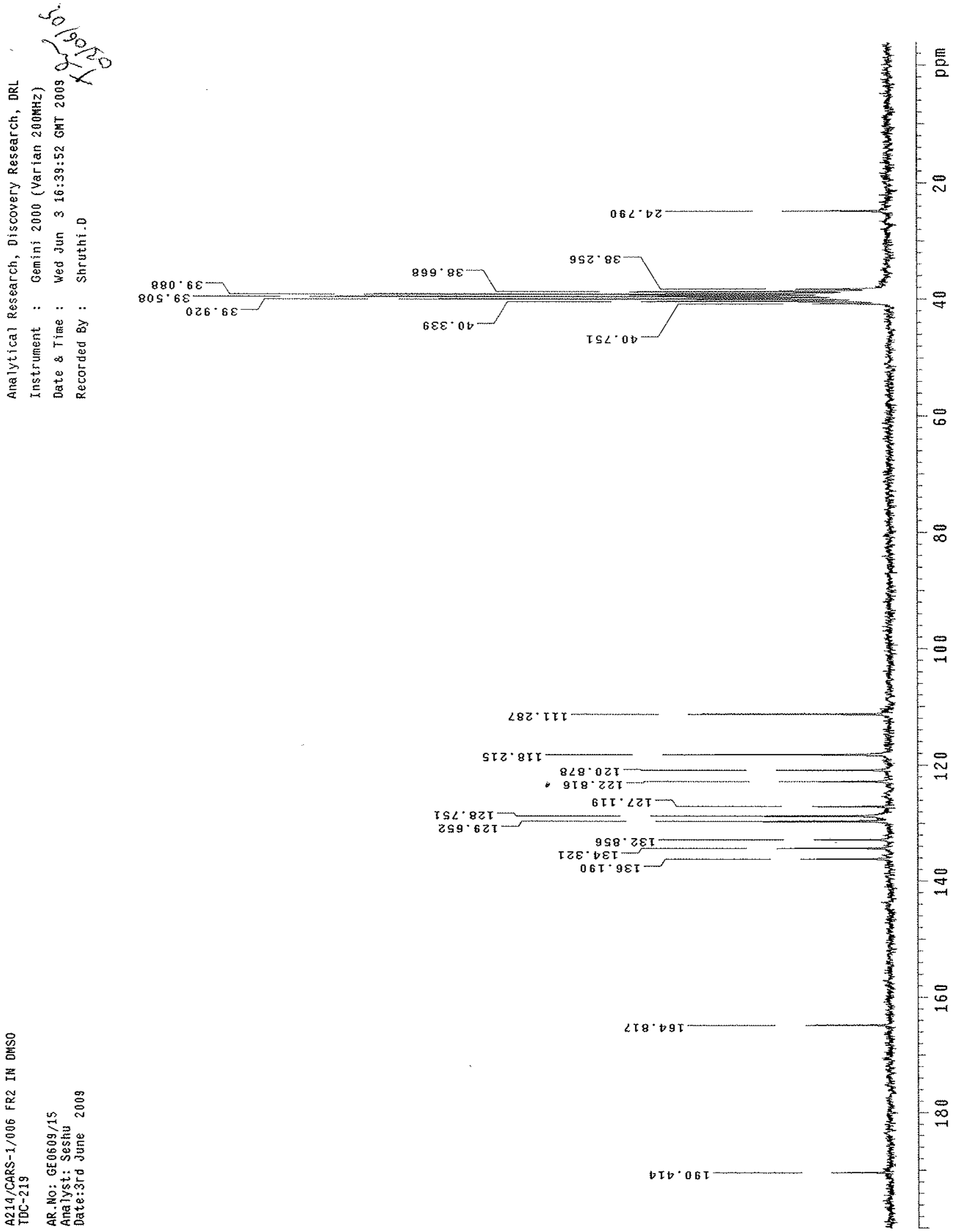

Mass spectrum of *N*-(2-(1*H*-indol-3-yl)ethyl)-2-oxo-2-phenylacetamide (**9a**):

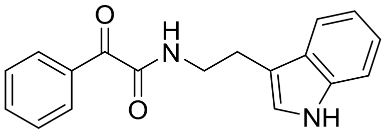

9a

CPS,MIYAPUR

Mass Analysis Report

**Data Filename**	08112413.d	**Sample Name**	A214/CARS-[illegible]/002 F-Y-5
**Sample Type**	Sample	**Position**	Vial 13
**Instrument Name**	Instrument 1	**User Name**	
**Acq Method**	ESI.m	**IRM Calibration Status**	Success
**DA Method**	DA.m	**Comment**	

User Spectra

Fragmentor Voltage	Collision Energy	Ionization Mode
135	0	Esi

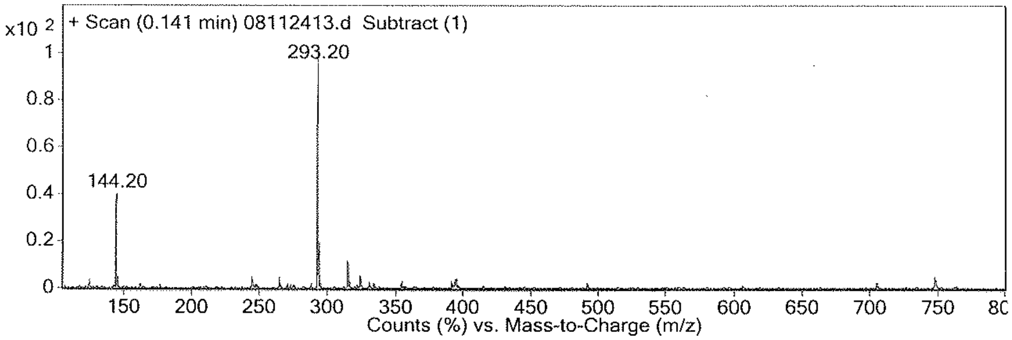

--- End Of Report ---

24/11/08

IR spectrum of *N*-(2-(1*H*-indol-3-yl)ethyl)-2-oxo-2-phenylacetamide (**9a**):

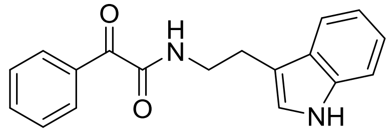

9a

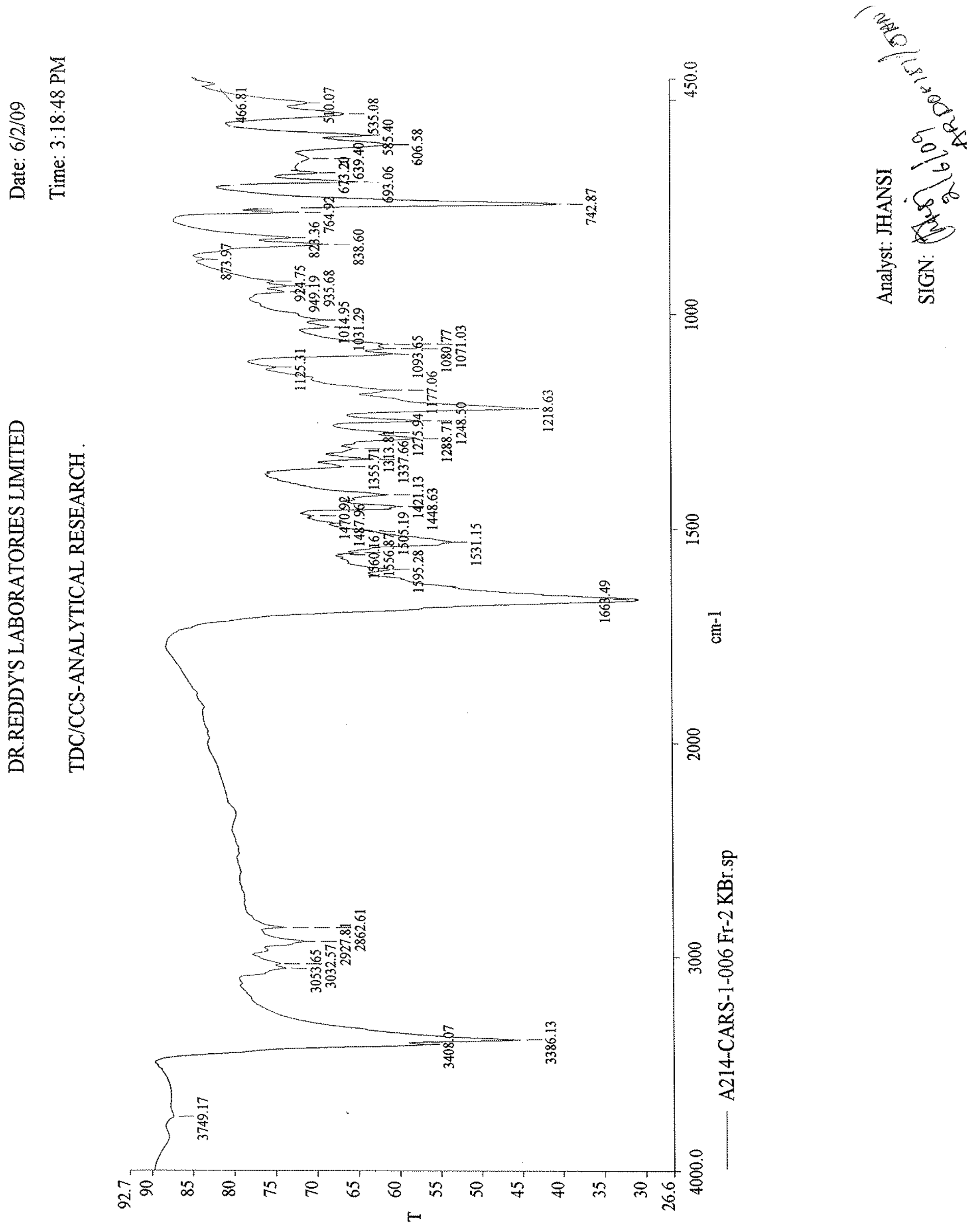

^{1}H NMR of *N*-(2-(1*H*-indol-3-yl)ethyl)-2-oxo-2-(*p*-tolyl)acetamide (**9b**):

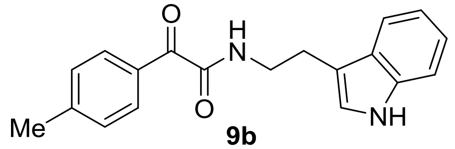

9b

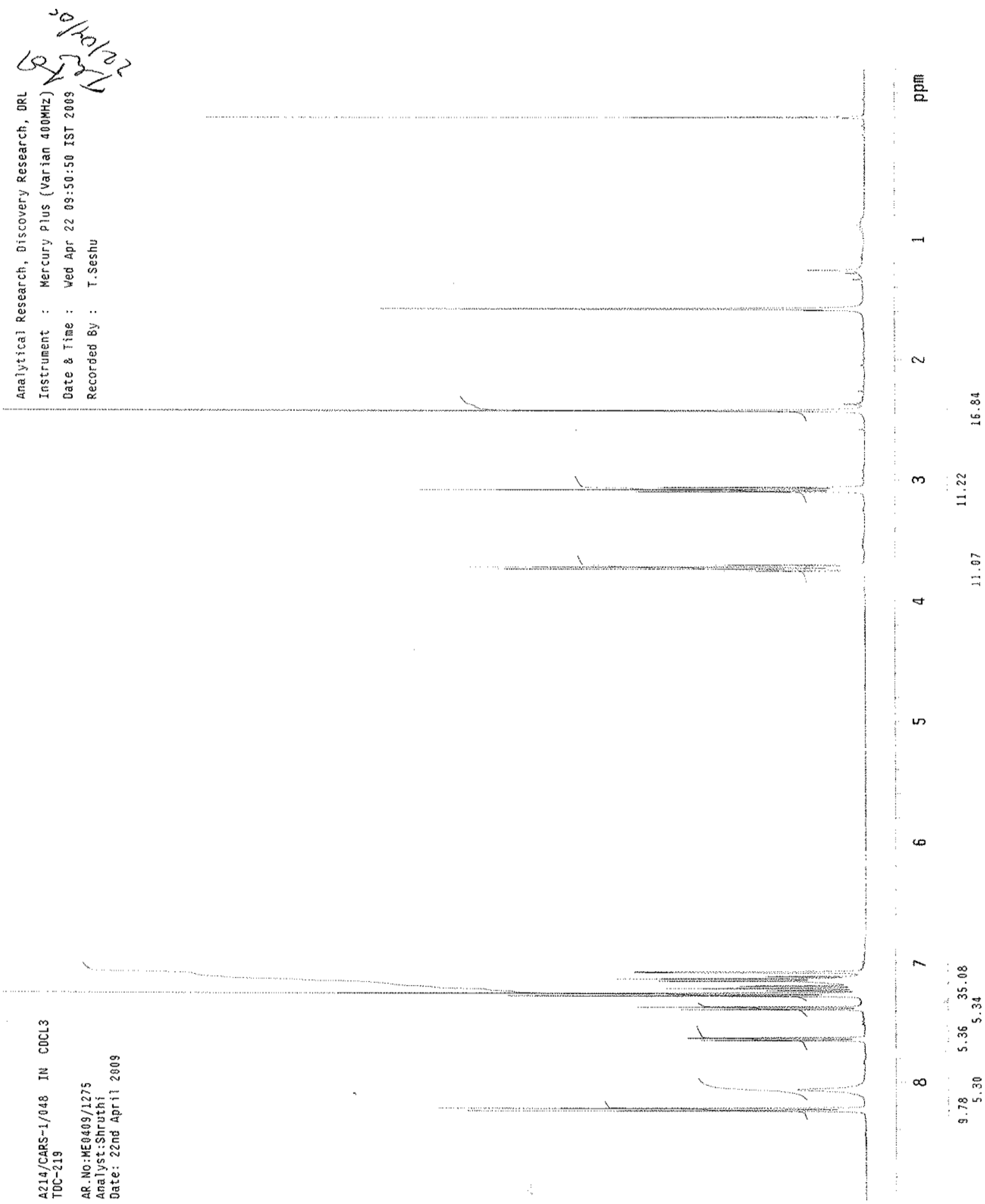

^{13}C NMR of *N*-(2-(1*H*-indol-3-yl)ethyl)-2-oxo-2-(*p*-tolyl)acetamide (**9b**):

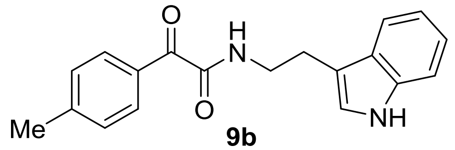

9b

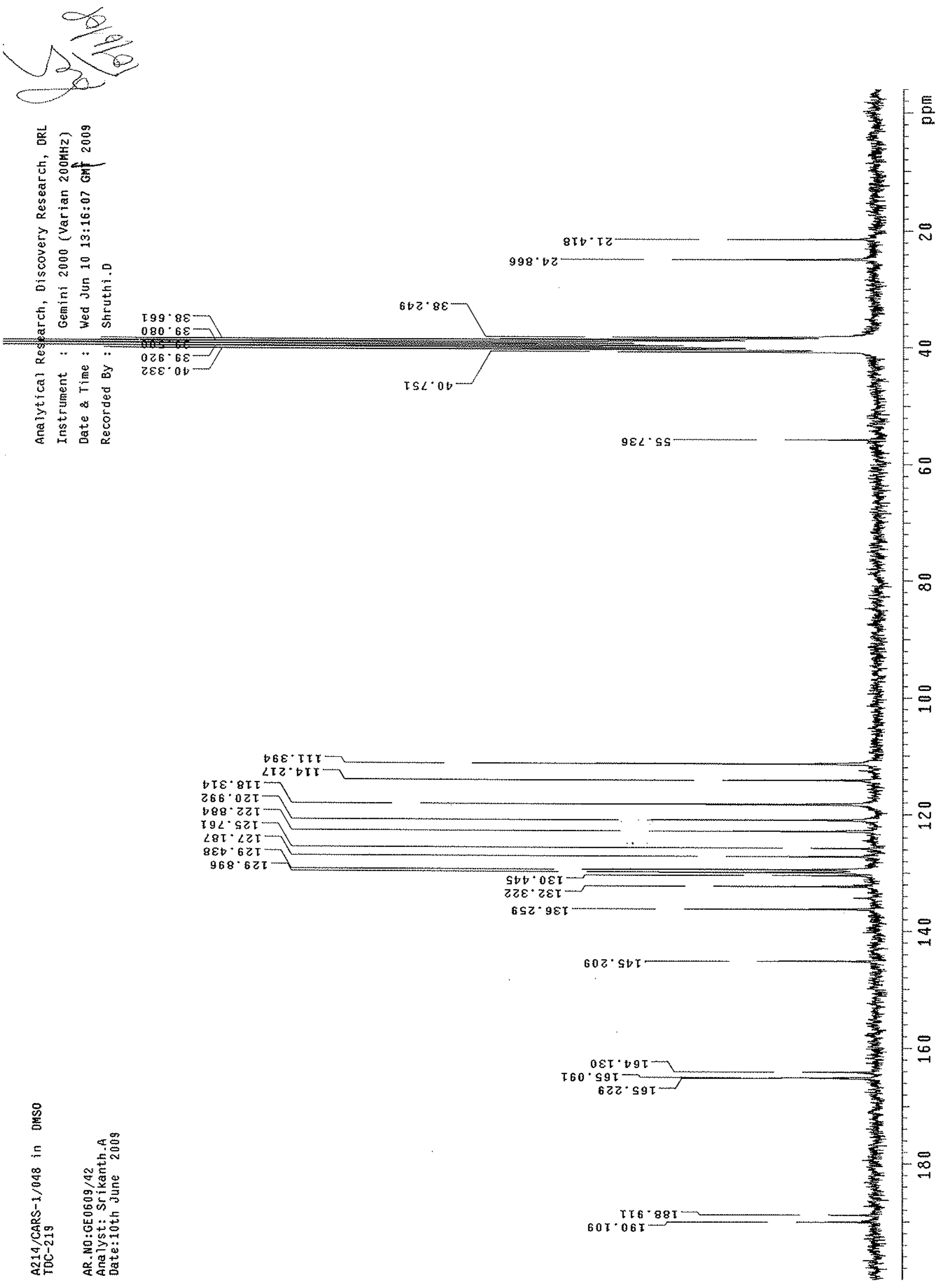

Mass spectrum of *N*-(2-(1*H*-indol-3-yl)ethyl)-2-oxo-2-(*p*-tolyl)acetamide (**9b**):

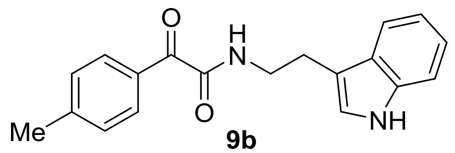

9b

CPS,MIYAPUR

Mass Analysis Report

**Data Filename**	090421015.d	**Sample Name**	A214/CARS-1/048
**Sample Type**	Sample	**Position**	Vial 54
**Instrument Name**	Instrument 1	**User Name**	
**Acq Method**		**IRM Calibration Status**	Success
**DA Method**	Quant Process.m	**Comment**	

User Spectra

Fragmentor Voltage	Collision Energy	Ionization Mode
135	0	Esi

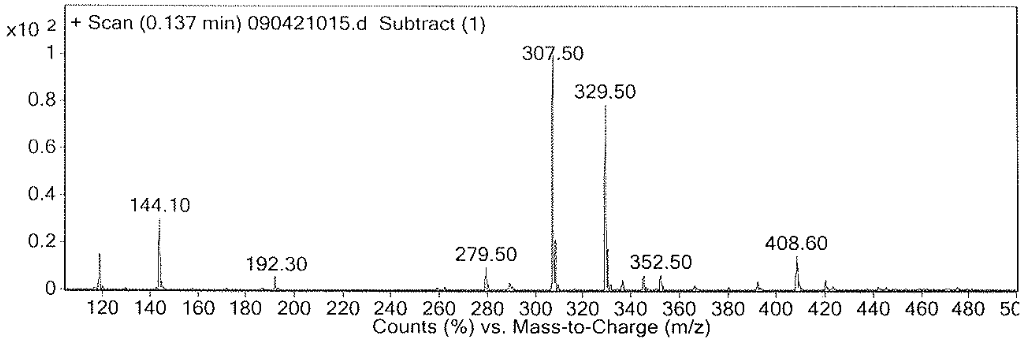

--- End Of Report ---

IR spectrum of *N*-(2-(1*H*-indol-3-yl)ethyl)-2-oxo-2-(*p*-tolyl)acetamide (**9b**):

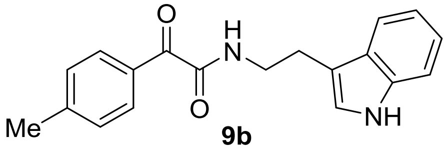

9b

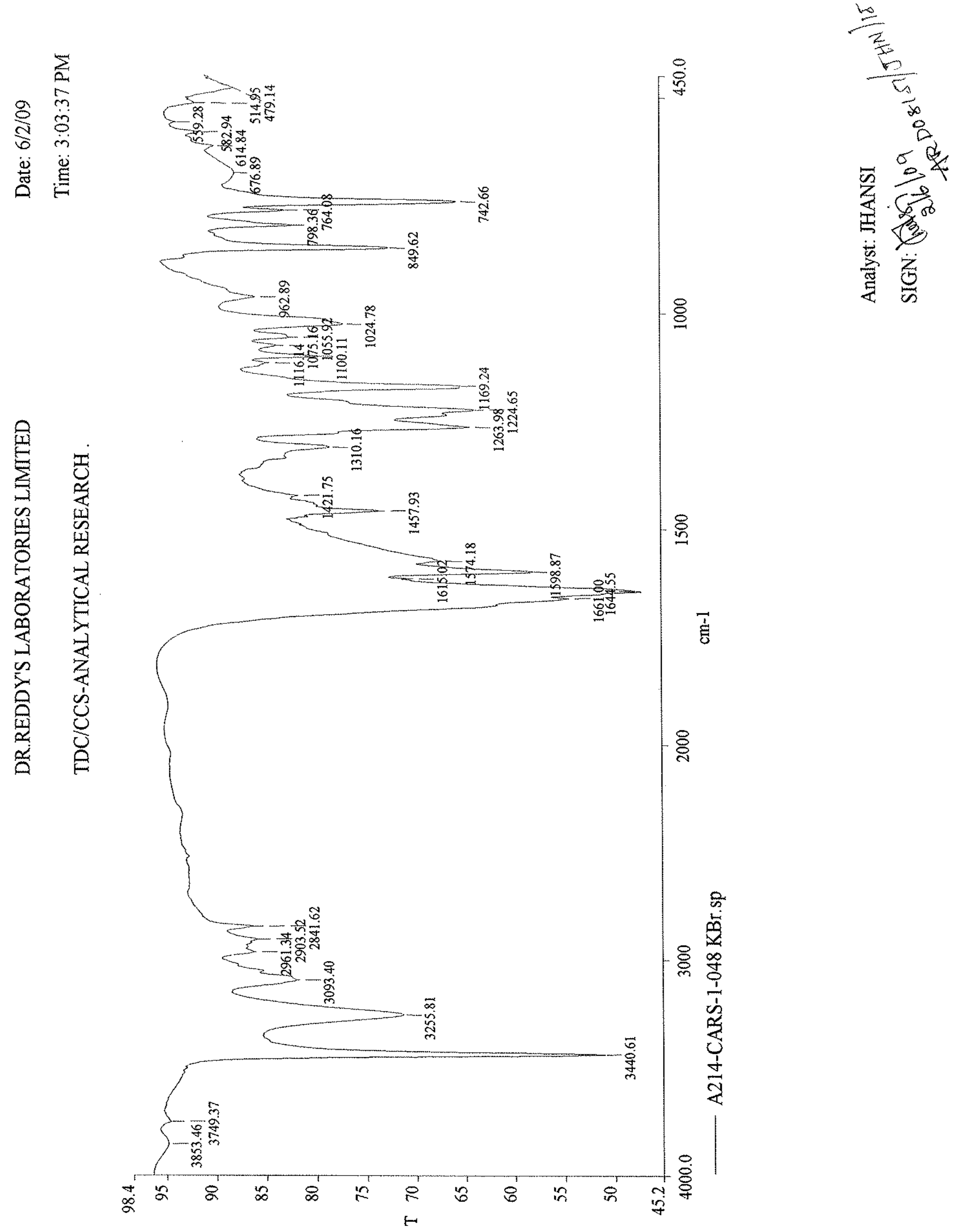

^{1}H NMR of *N*-(2-(1*H*-indol-3-yl)ethyl)-2-oxo-2-(4-(trifluoromethyl)phenyl)acetamide (**9c**):

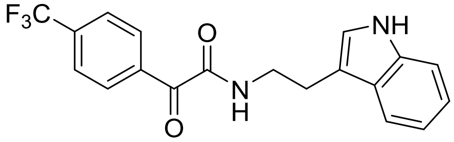

9c

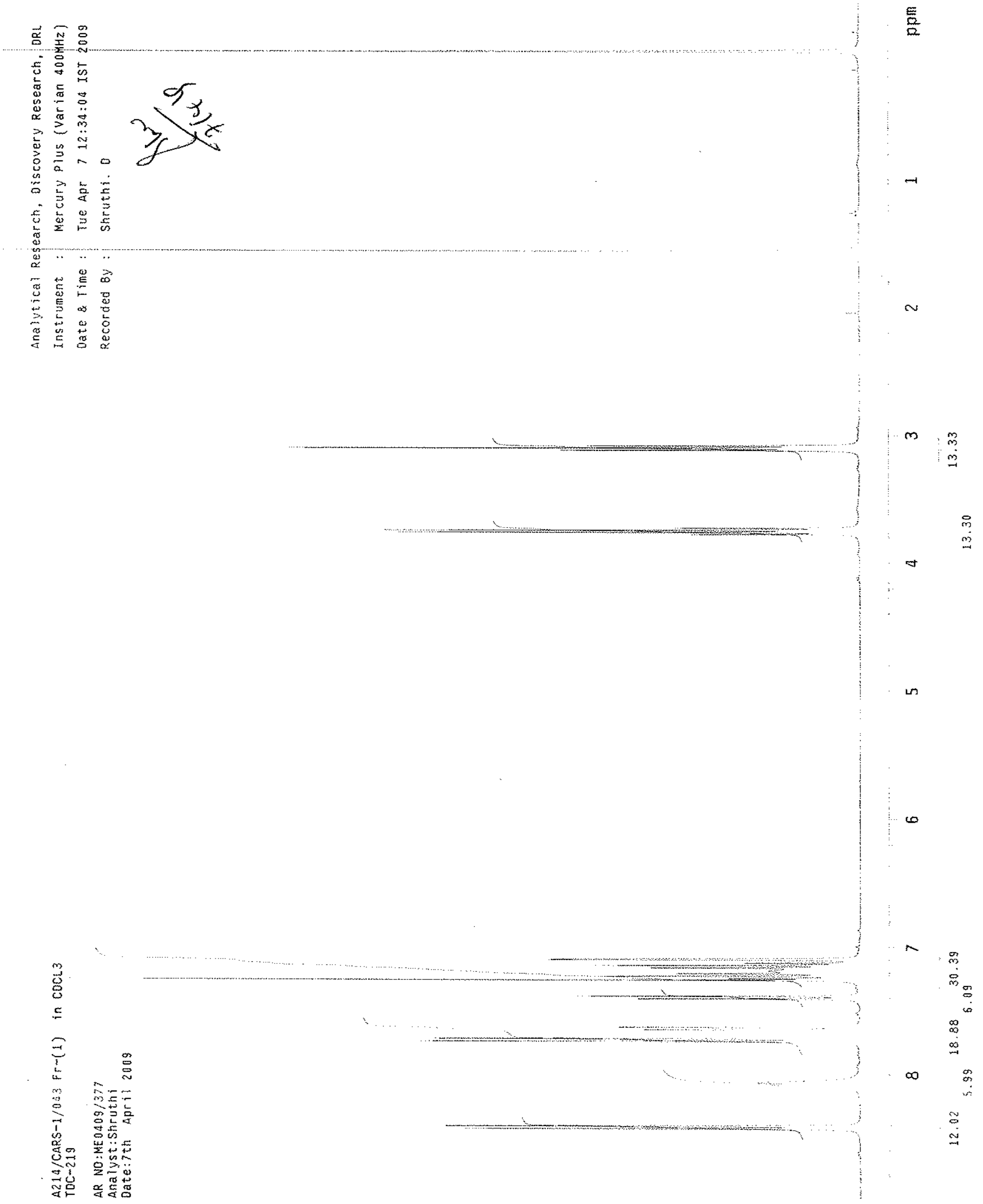

^{13}C NMR of *N*-(2-(1*H*-indol-3-yl)ethyl)-2-oxo-2-(4-(trifluoromethyl)phenyl)acetamide (**9c**):

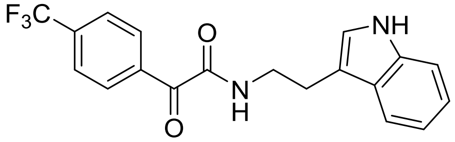

9c

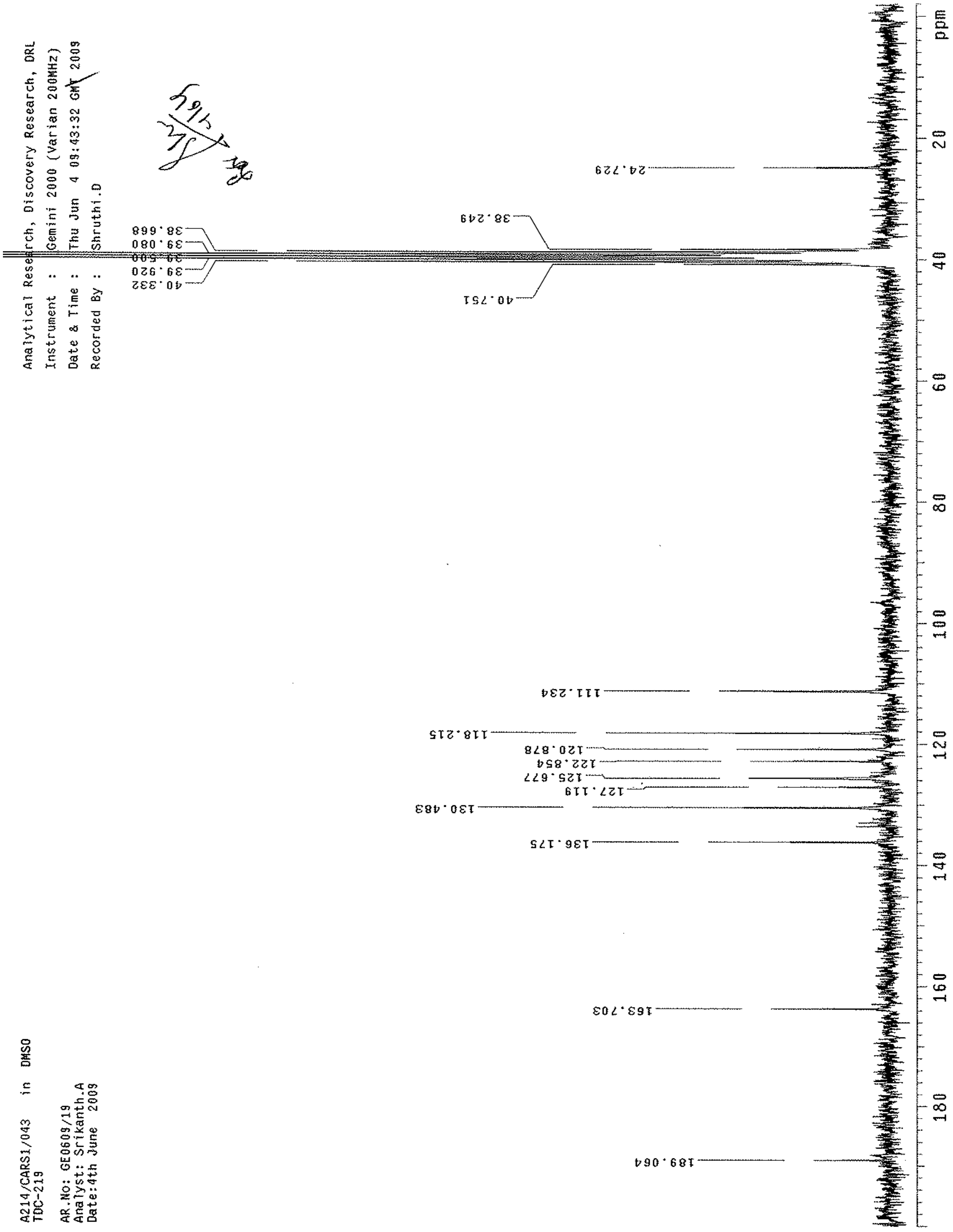

Mass spectrum of *N*-(2-(1*H*-indol-3-yl)ethyl)-2-oxo-2-(4-(trifluoromethyl)phenyl)acetamide (**9c**):

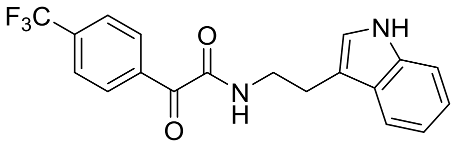

9c

CPS,MIYAPUR

Mass Analysis Report

**Data Filename**	090407032.d	**Sample Name**	A214/CARS-1/043 FR-1
**Sample Type**	Sample	**Position**	Vial 78
**Instrument Name**	Instrument 1	**User Name**	
**Acq Method**	ESI.m	**IRM Calibration Status**	Success
**DA Method**	ESMS.m	**Comment**	

User Spectra

Fragmentor Voltage	Collision Energy	Ionization Mode
100	0	Esi

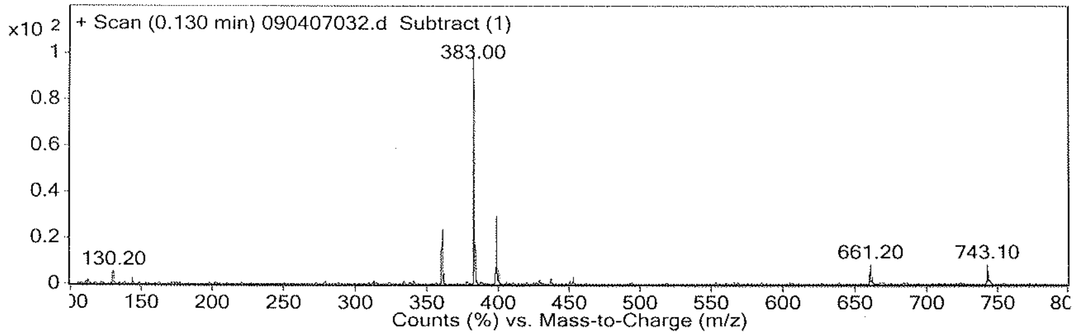

--- End Of Report ---

07/04/09

IR spectrum of *N*-(2-(1*H*-indol-3-yl)ethyl)-2-oxo-2-(4-(trifluoromethyl)phenyl)acetamide (**9c**):

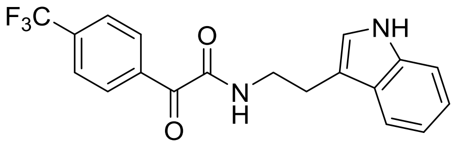

9c

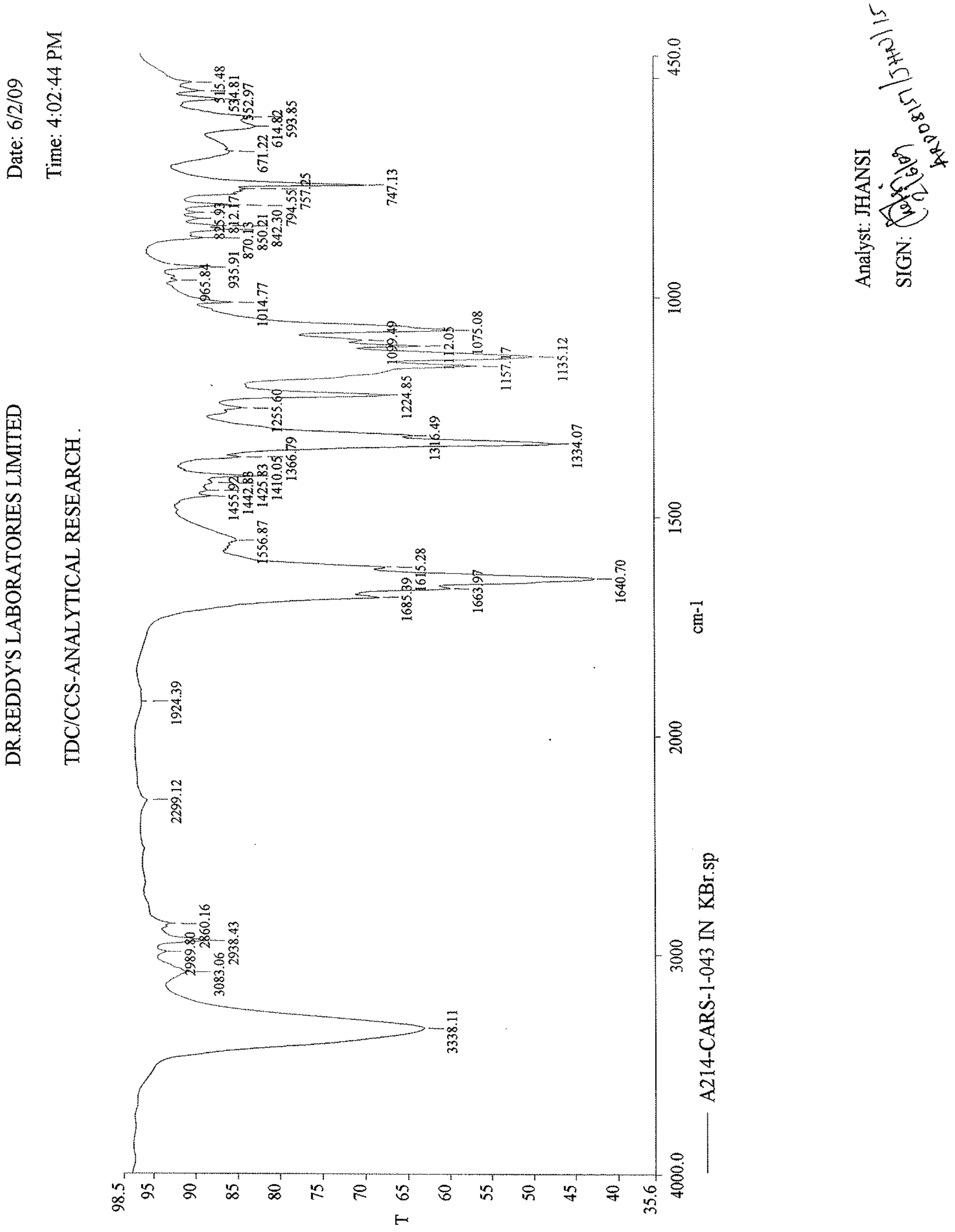

^{1}H NMR of *N*-(2-(1*H*-indol-3-yl)ethyl)-2-(4-fluorophenyl)-2-oxoacetamide

(**9d**): 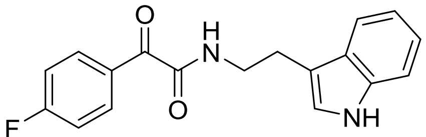

9d

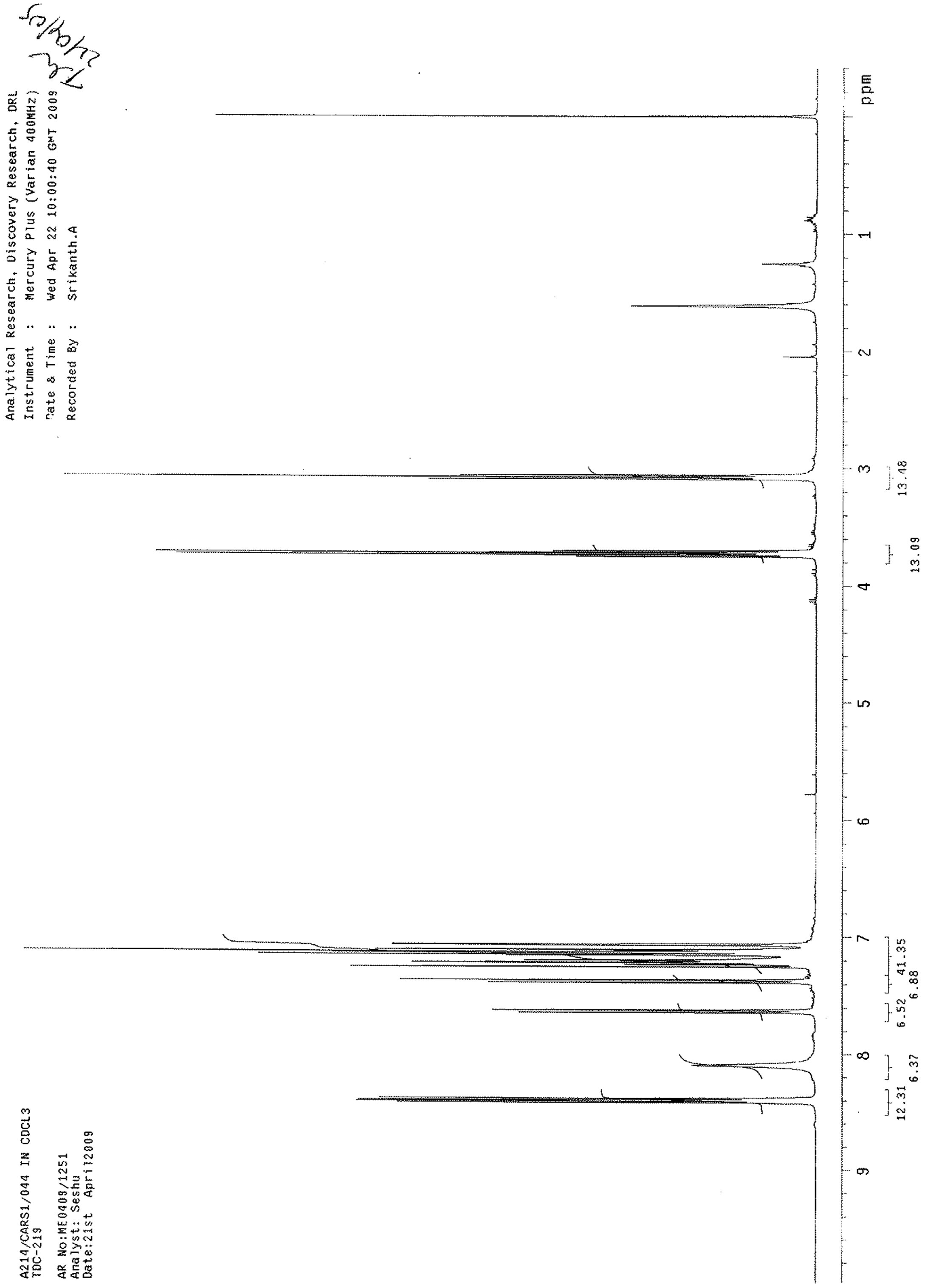

^{13}C NMR of *N*-(2-(1*H*-indol-3-yl)ethyl)-2-(4-fluorophenyl)-2-oxoacetamide (**9d**):

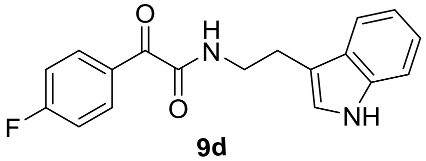

9d

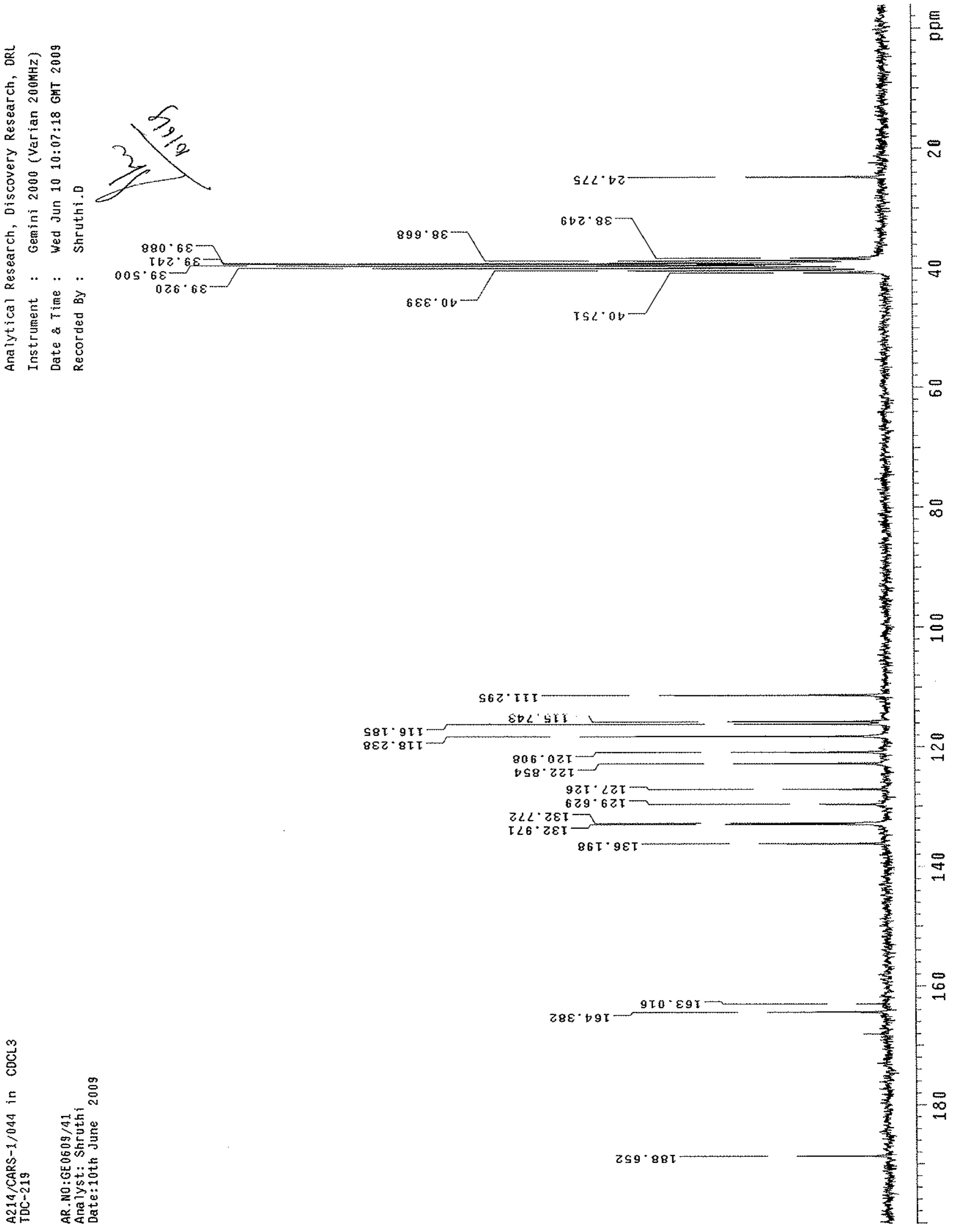

Mass spectrum of *N*-(2-(1*H*-indol-3-yl)ethyl)-2-(4-fluorophenyl)-2-oxoacetamide (**9d**):

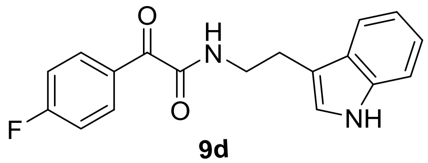

9d

CPS,MIYAPUR

Mass Analysis Report

**Data Filename**	090421014.d	**Sample Name**	A214/CARS-1/044
**Sample Type**	Sample	**Position**	Vial 53
**Instrument Name**	Instrument 1	**User Name**	
**Acq Method**		**IRM Calibration Status**	Success
**DA Method**	Quant Process.m	**Comment**	

User Spectra

Fragmentor Voltage	Collision Energy	Ionization Mode
135	0	Esi

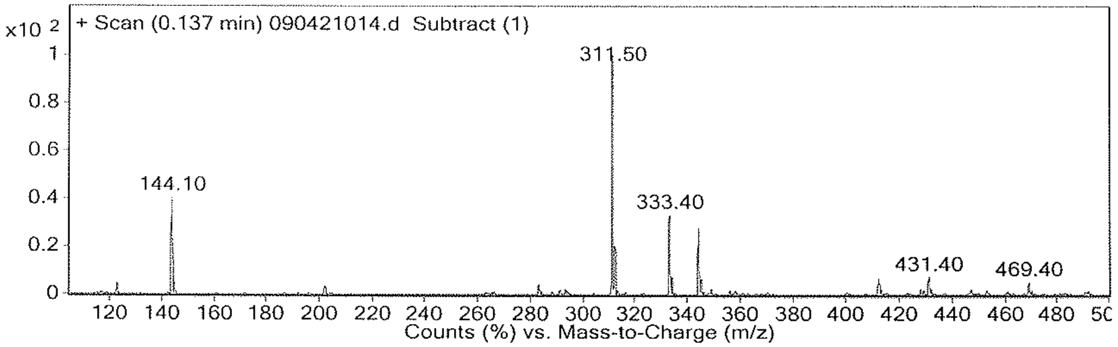

--- End Of Report ---

21/04/09

IR spectrum of *N*-(2-(1*H*-indol-3-yl)ethyl)-2-(4-fluorophenyl)-2-oxoacetamide (**9d**):

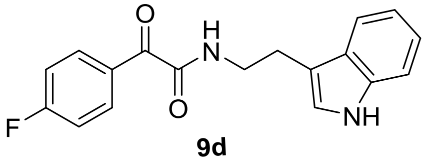

9d

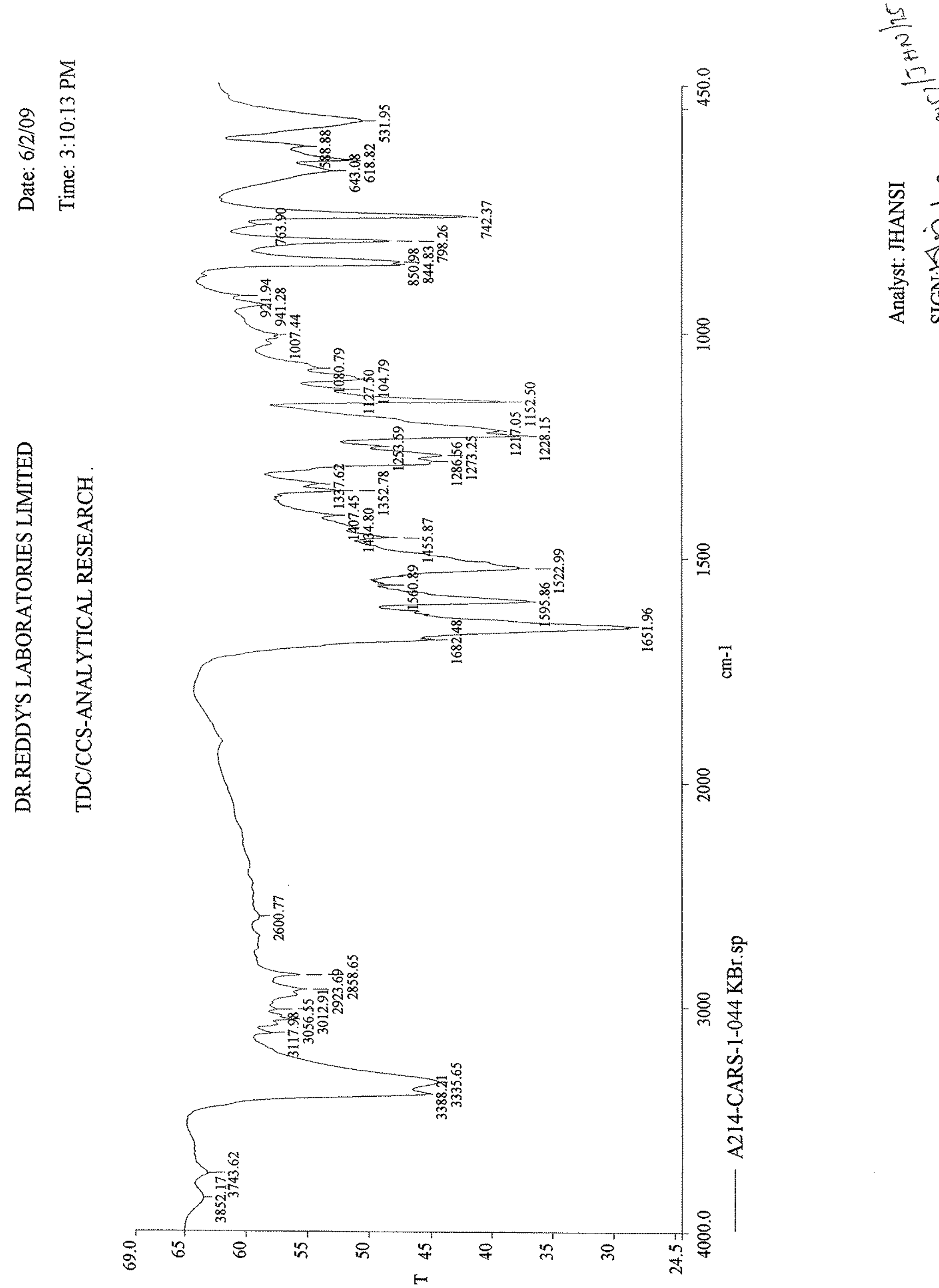

^{1}H NMR of *N*-(2-(1*H*-indol-3-yl)ethyl)-2-(3-nitrophenyl)-2-oxoacetamide (**9e**):

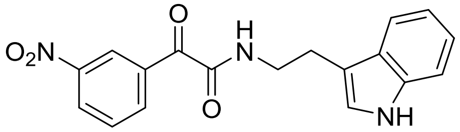

9e

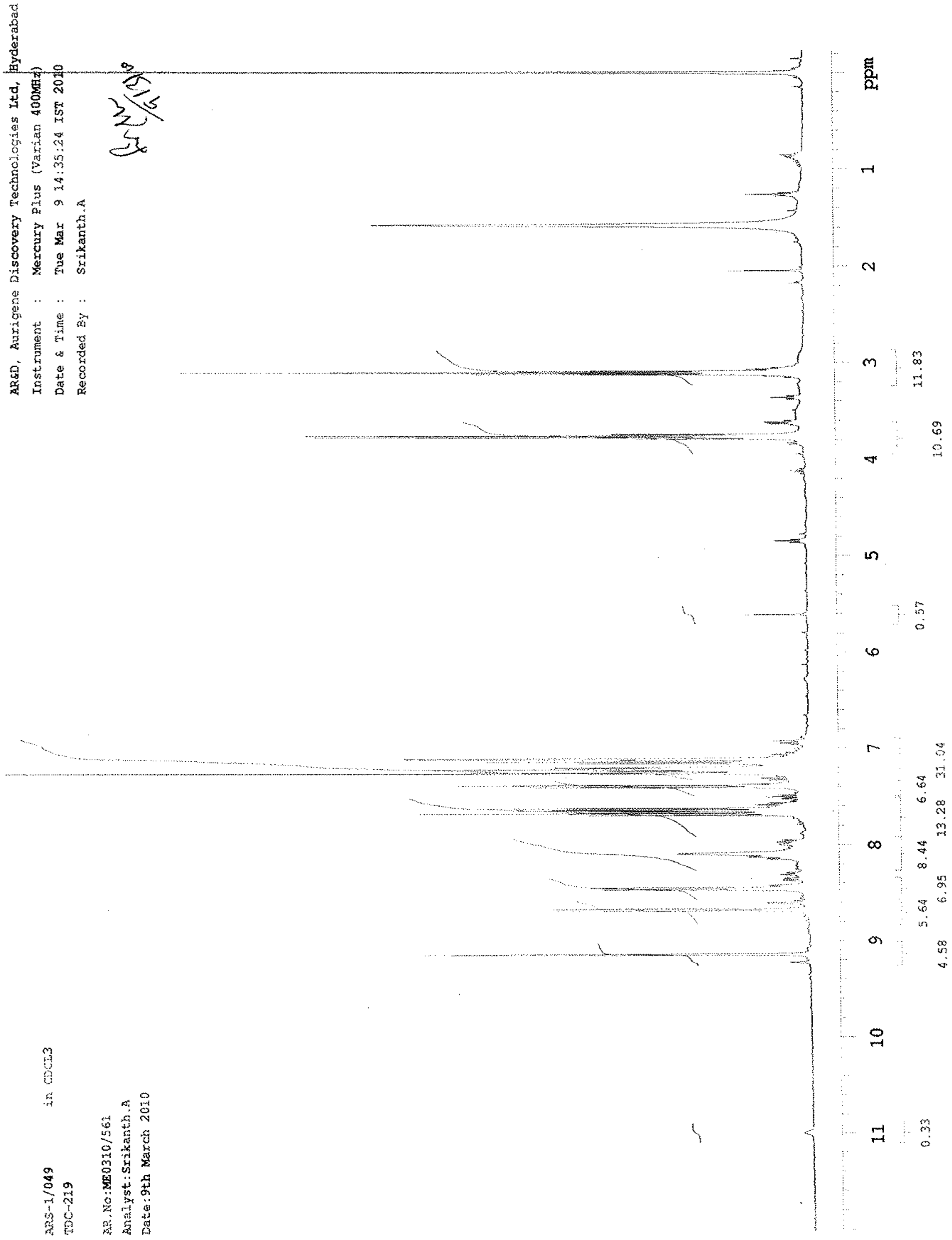

^{13}C NMR of *N*-(2-(1*H*-indol-3-yl)ethyl)-2-(3-nitrophenyl)-2-oxoacetamide (**9e**):

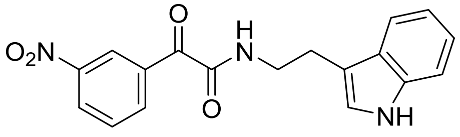

9e

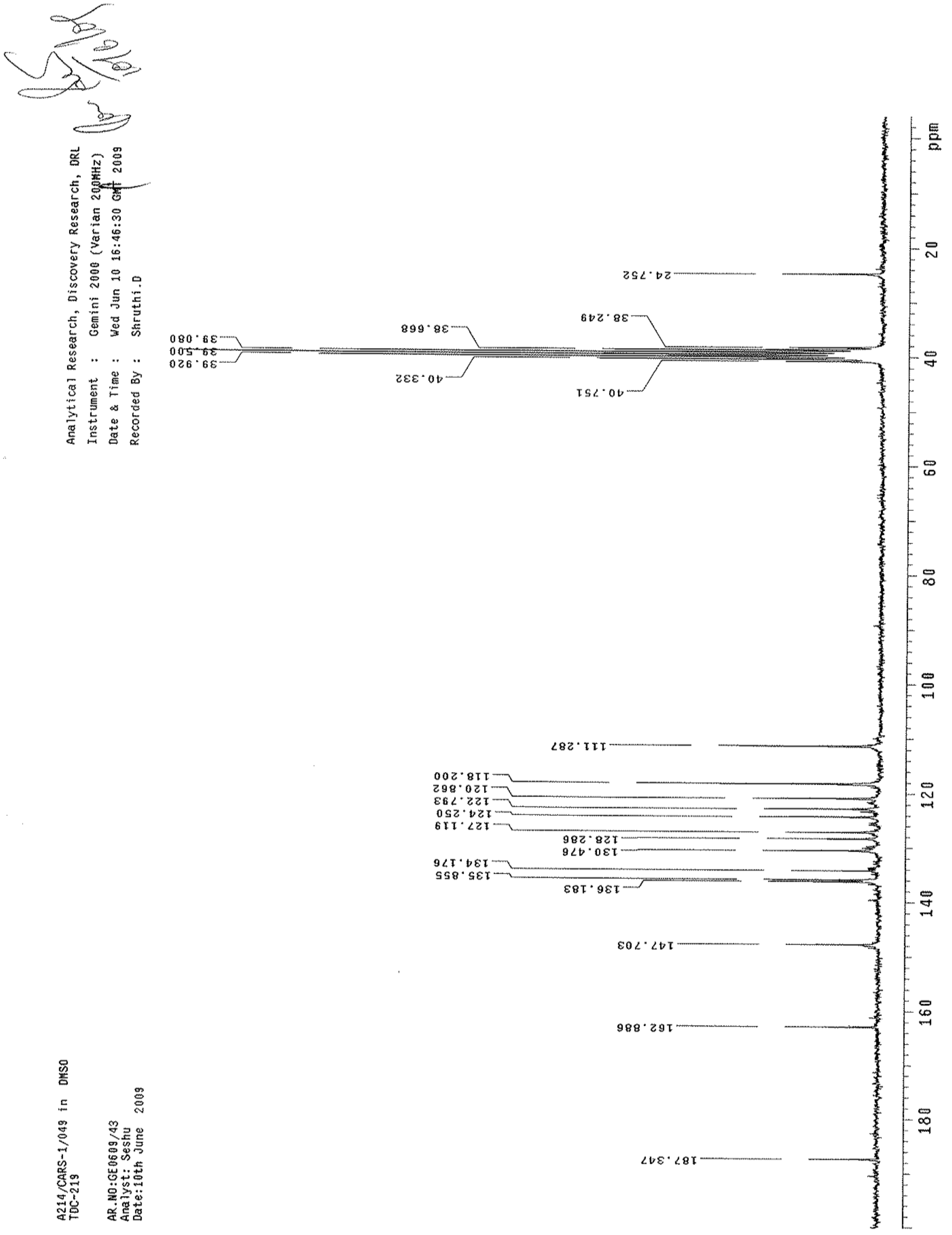

Mass spectrum of *N*-(2-(1*H*-indol-3-yl)ethyl)-2-(3-nitrophenyl)-2-oxoacetamide (**9e**):

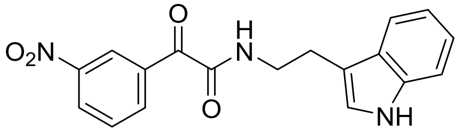

9e

CPS,MIYAPUR

Mass Analysis Report

**Data Filename**	090428027.d	**Sample Name**	A214/CARS-1/049
**Sample Type**	Sample	**Position**	Vial 82
**Instrument Name**	Instrument 1	**User Name**	
**Acq Method**		**IRM Calibration Status**	Success
**DA Method**	Quant Process.m	**Comment**	

User Spectra

Fragmentor Voltage	Collision Energy	Ionization Mode
100	0	Esi

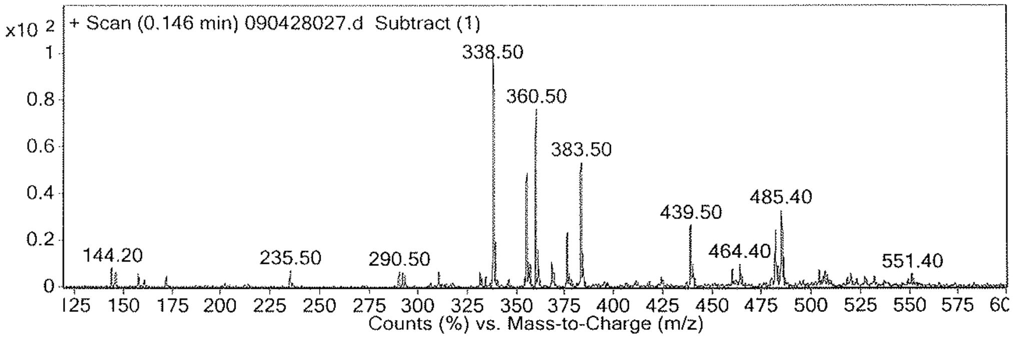

--- End Of Report ---

[illegible] 28/04/09

IR spectrum of *N*-(2-(1*H*-indol-3-yl)ethyl)-2-(3-nitrophenyl)-2-oxoacetamide (**9e**):

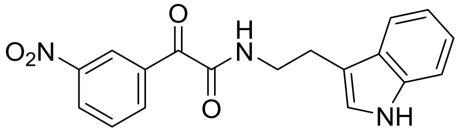

9e

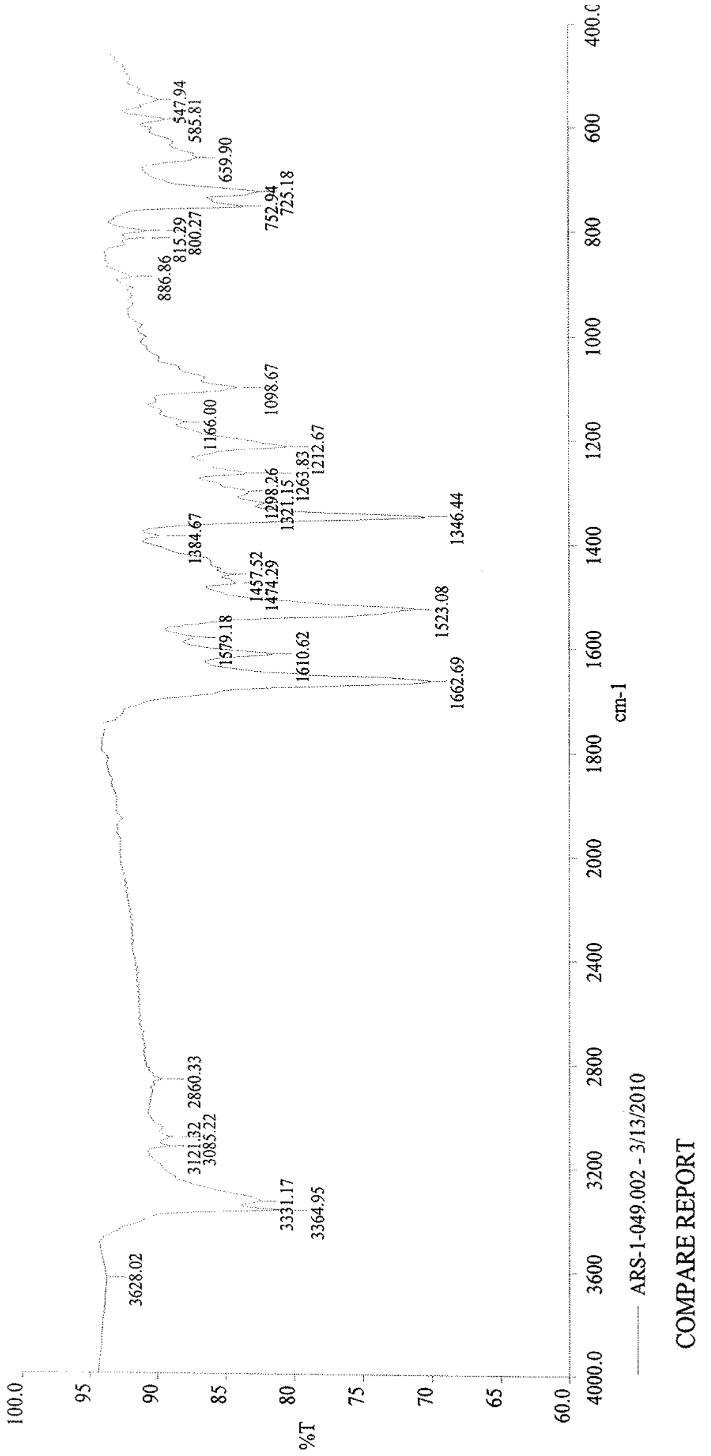

^{1}H NMR of *N*-(2-(1*H*-indol-3-yl)ethyl)-2-(4-methoxyphenyl)-2-oxoacetamide (**9f**):

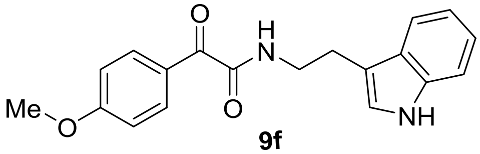

9f

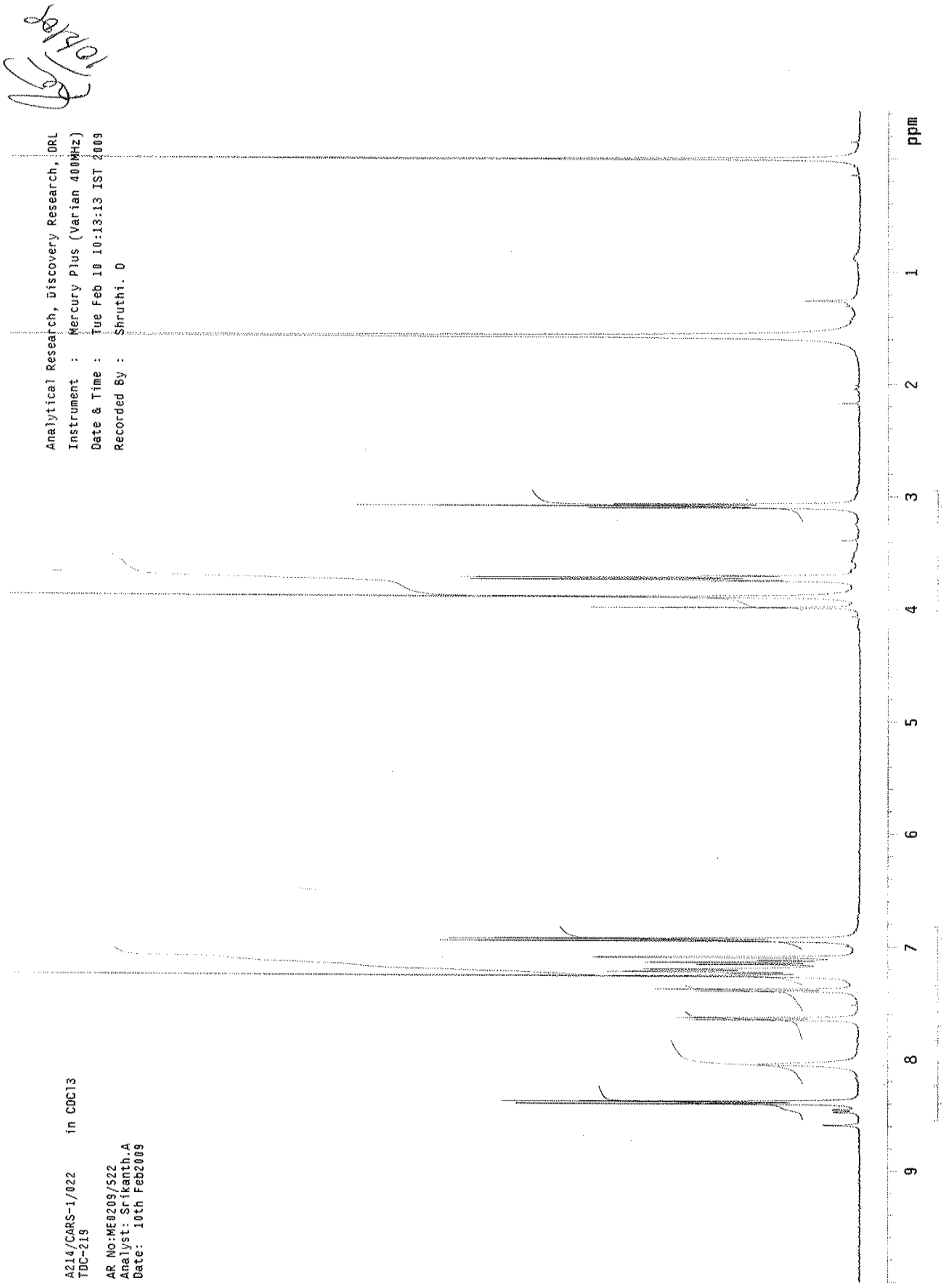

^{13}C NMR of *N*-(2-(1*H*-indol-3-yl)ethyl)-2-(4-methoxyphenyl)-2-oxoacetamide (**9f**):

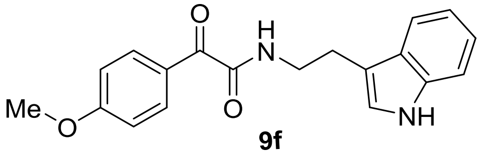

9f

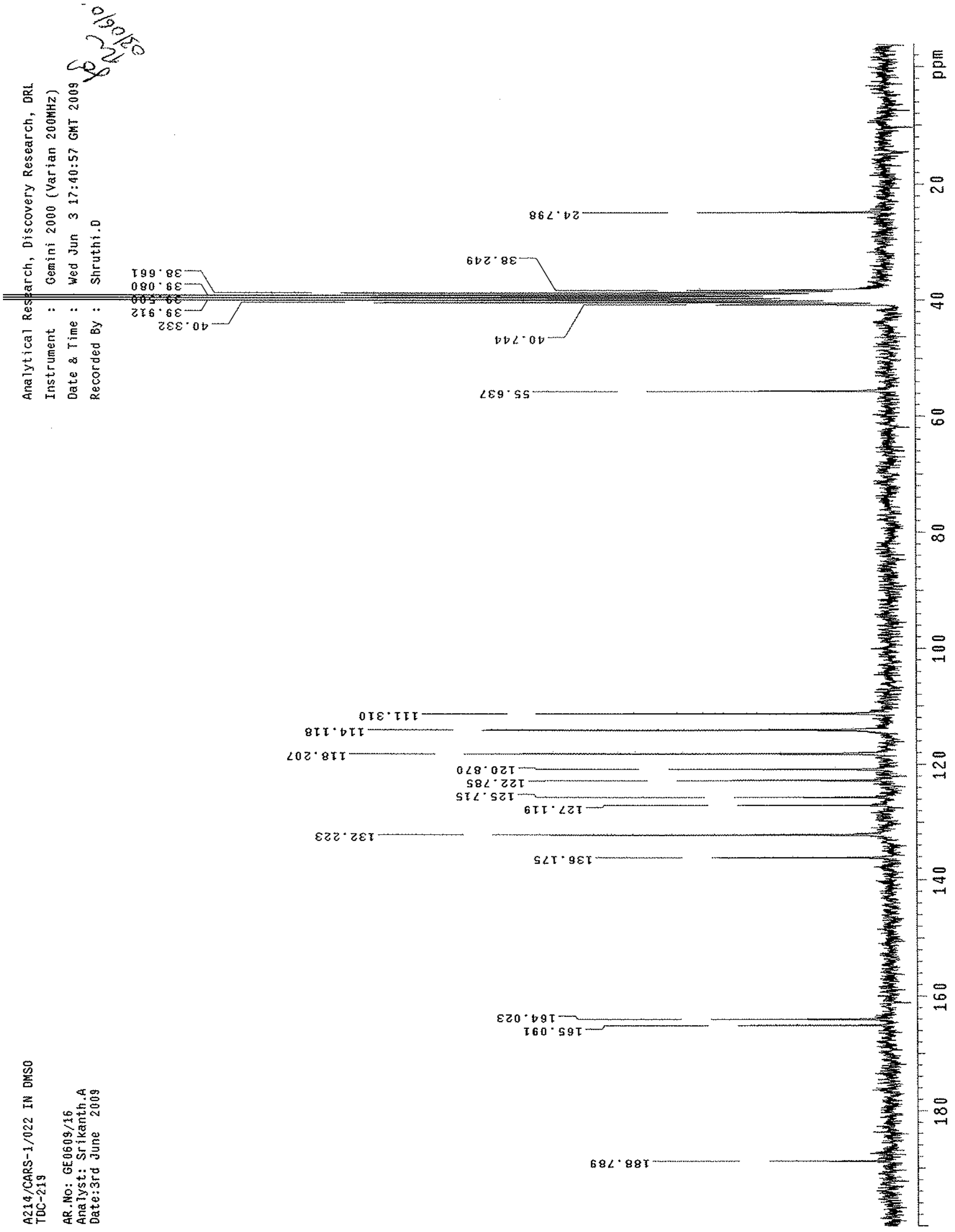

Mass spectrum of *N*-(2-(1*H*-indol-3-yl)ethyl)-2-(4-methoxyphenyl)-2-oxoacetamide (**9f**):

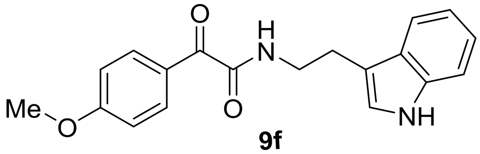

9f

CPS,MIYAPUR

Mass Analysis Report

**Data Filename**	090211031.d	**Sample Name**	A214/CARS/022
**Sample Type**	Sample	**Position**	Vial 30
**Instrument Name**	Instrument 1	**User Name**	
**Acq Method**	ESI.m	**IRM Calibration Status**	Success
**DA Method**	DA.m	**Comment**	

User Spectra

Fragmentor Voltage	Collision Energy	Ionization Mode
70	0	Esi

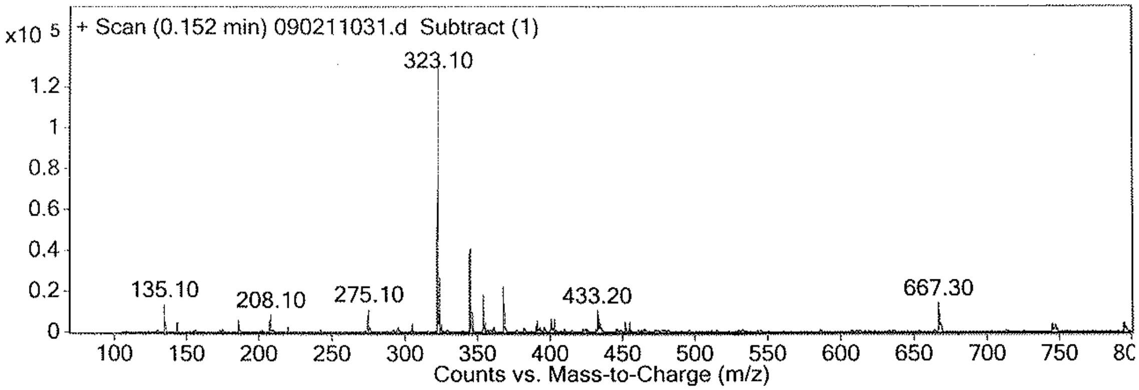

--- End Of Report ---

IR spectrum of *N*-(2-(1*H*-indol-3-yl)ethyl)-2-(4-methoxyphenyl)-2-oxoacetamide (**9f**):

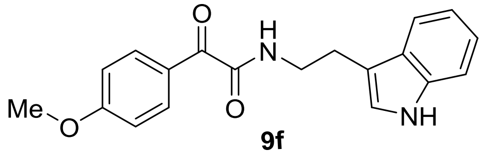

9f

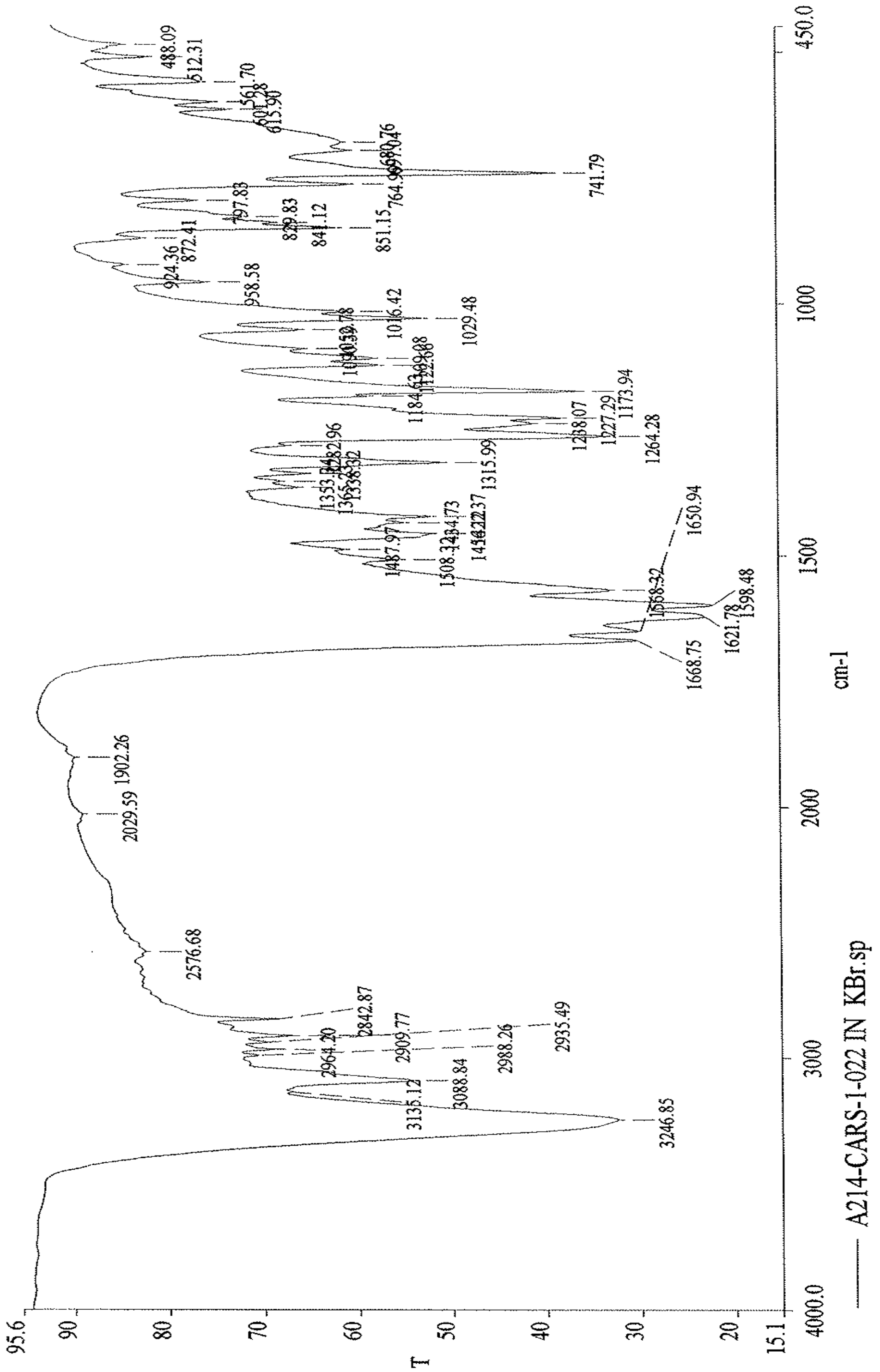

^{1}H NMR of *N*-(2-(1*H*-indol-3-yl)ethyl)-2-(2,4-dichlorophenyl)-2-oxoacetamide (**9g**):

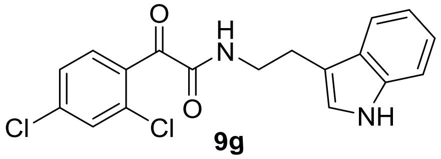

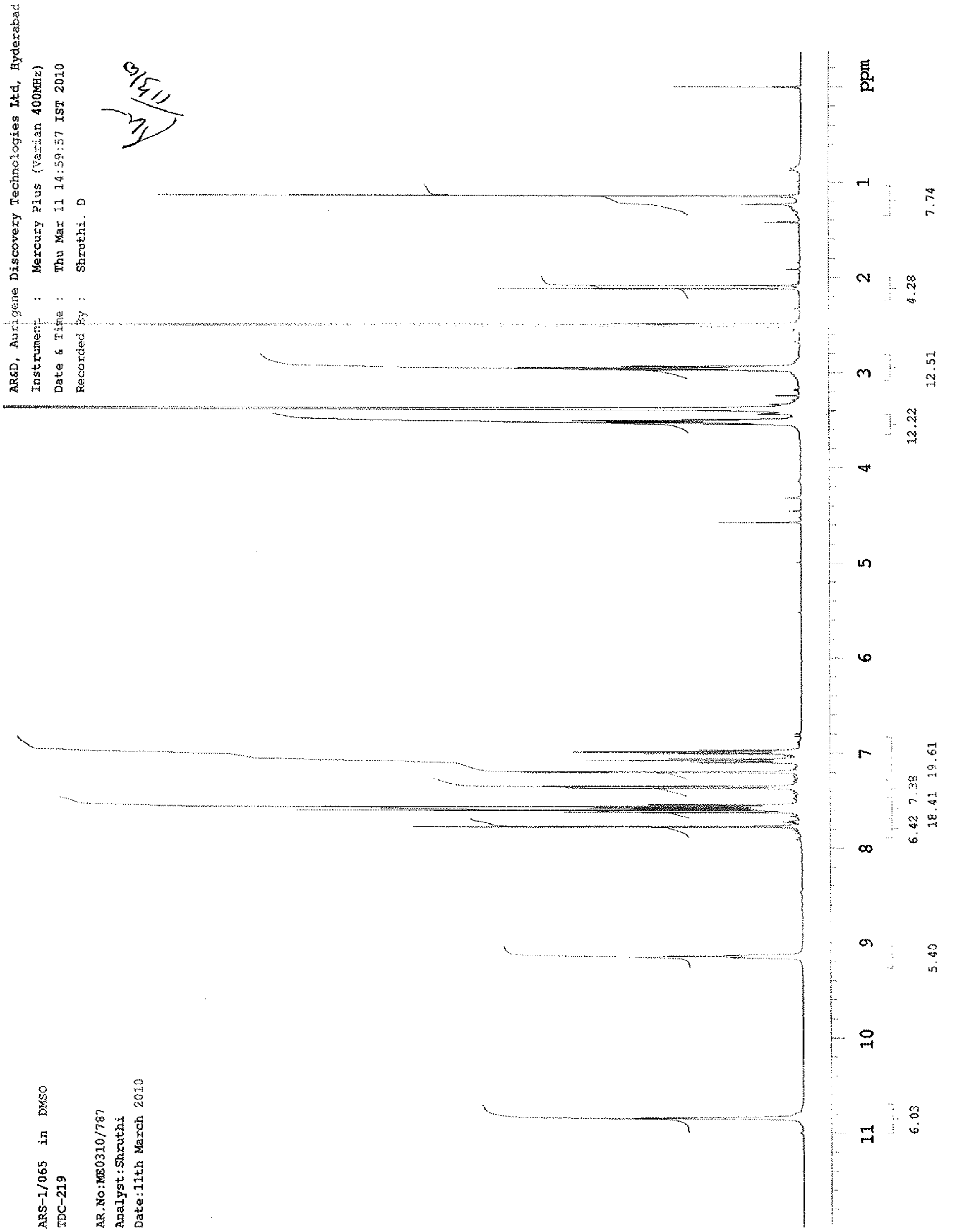

^{13}C NMR of *N*-(2-(1*H*-indol-3-yl)ethyl)-2-(2,4-dichlorophenyl)-2-oxoacetamide (**9g**):

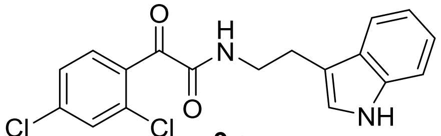

9g

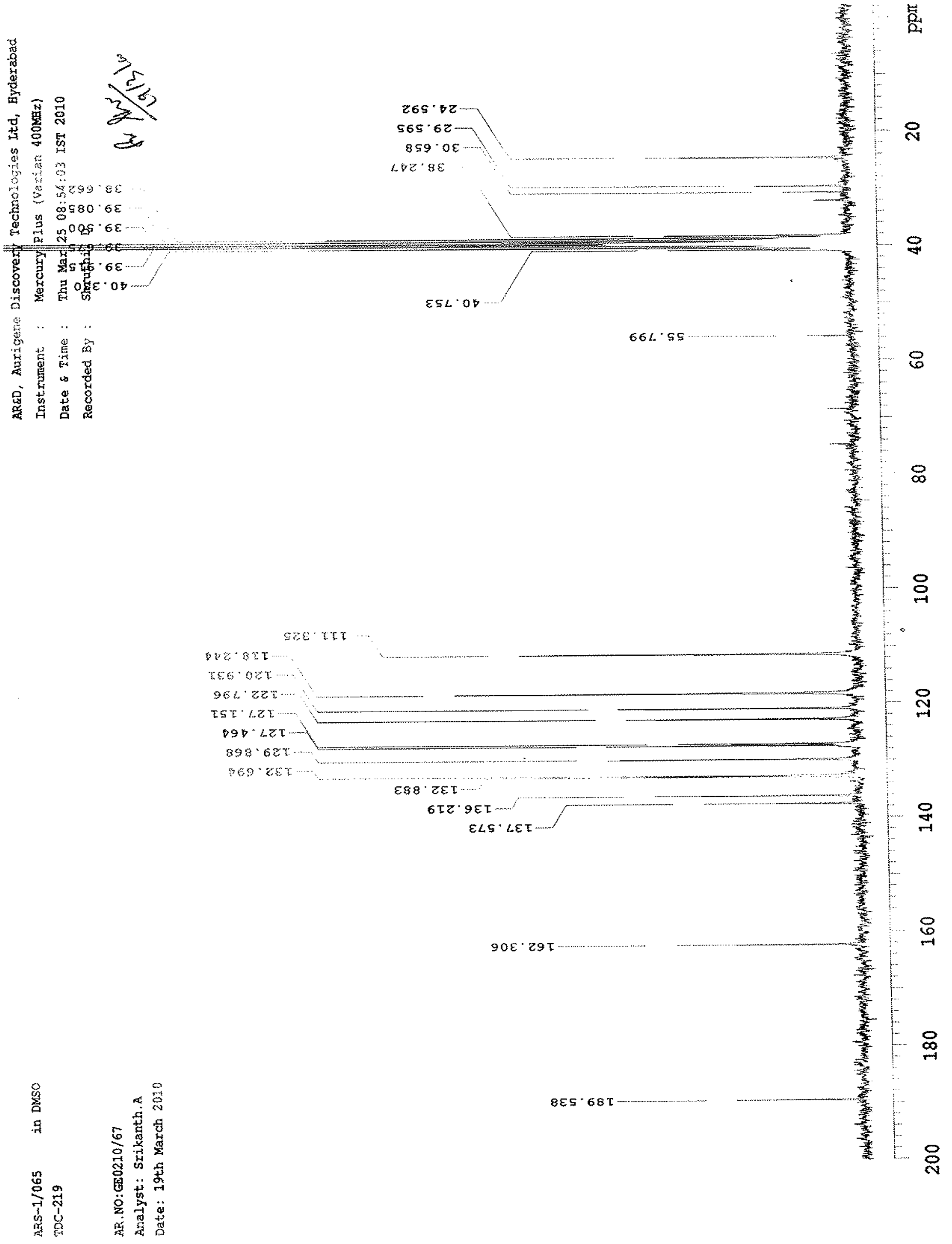

Mass spectrum of *N*-(2-(1*H*-indol-3-yl)ethyl)-2-(2,4-dichlorophenyl)-2-oxoacetamide (**9g**):

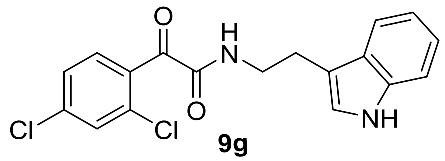

9g

CPS,MIYAPUR

Mass Analysis Report

**Data Filename**	100308022.d	**Sample Name**	ARS-1/065
**Sample Type**	Sample	**Position**	Vial 62
**Instrument Name**	Instrument 1	**User Name**	
**Acq Method**	ESI.m	**IRM Calibration Status**	Success
**DA Method**	default.m	**Comment**	

User Spectra

Fragmentor Voltage	Collision Energy	Ionization Mode
100	0	Esi

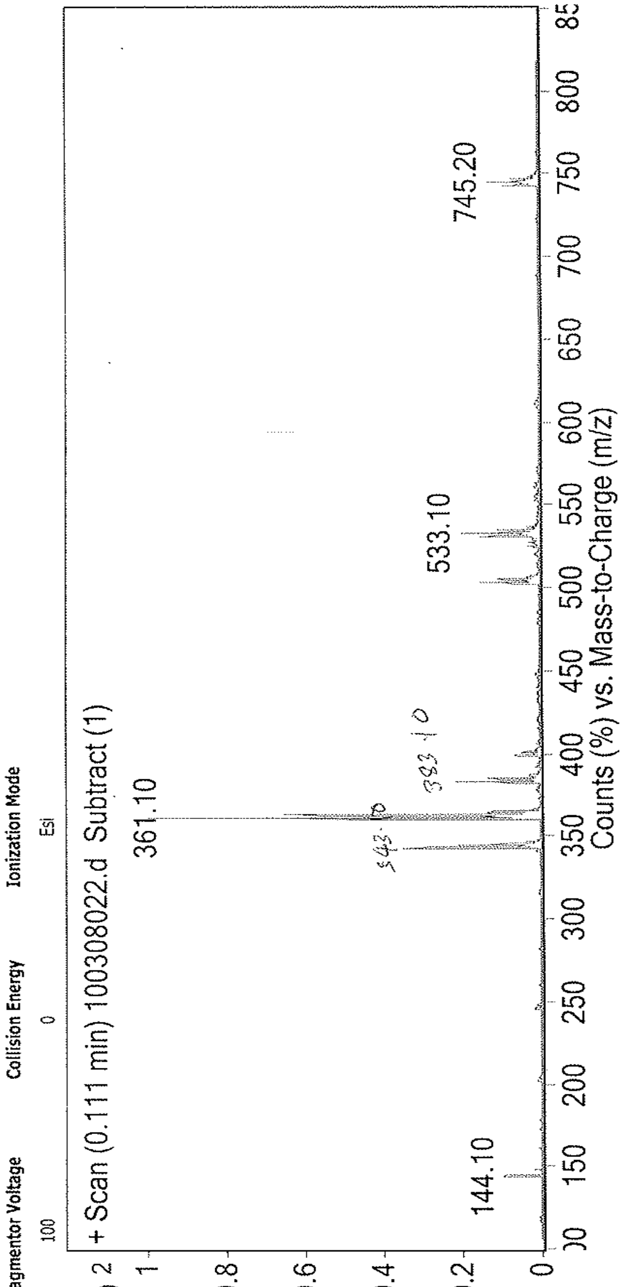

--- End Of Report ---

^{1}H NMR of *N*-(2-(1*H*-indol-3-yl)ethyl)-2-(4-(benzyloxy)phenyl)-2-oxoacetamide (**9h**):

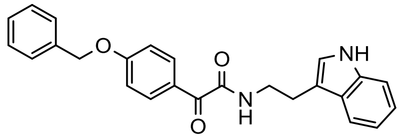

9h

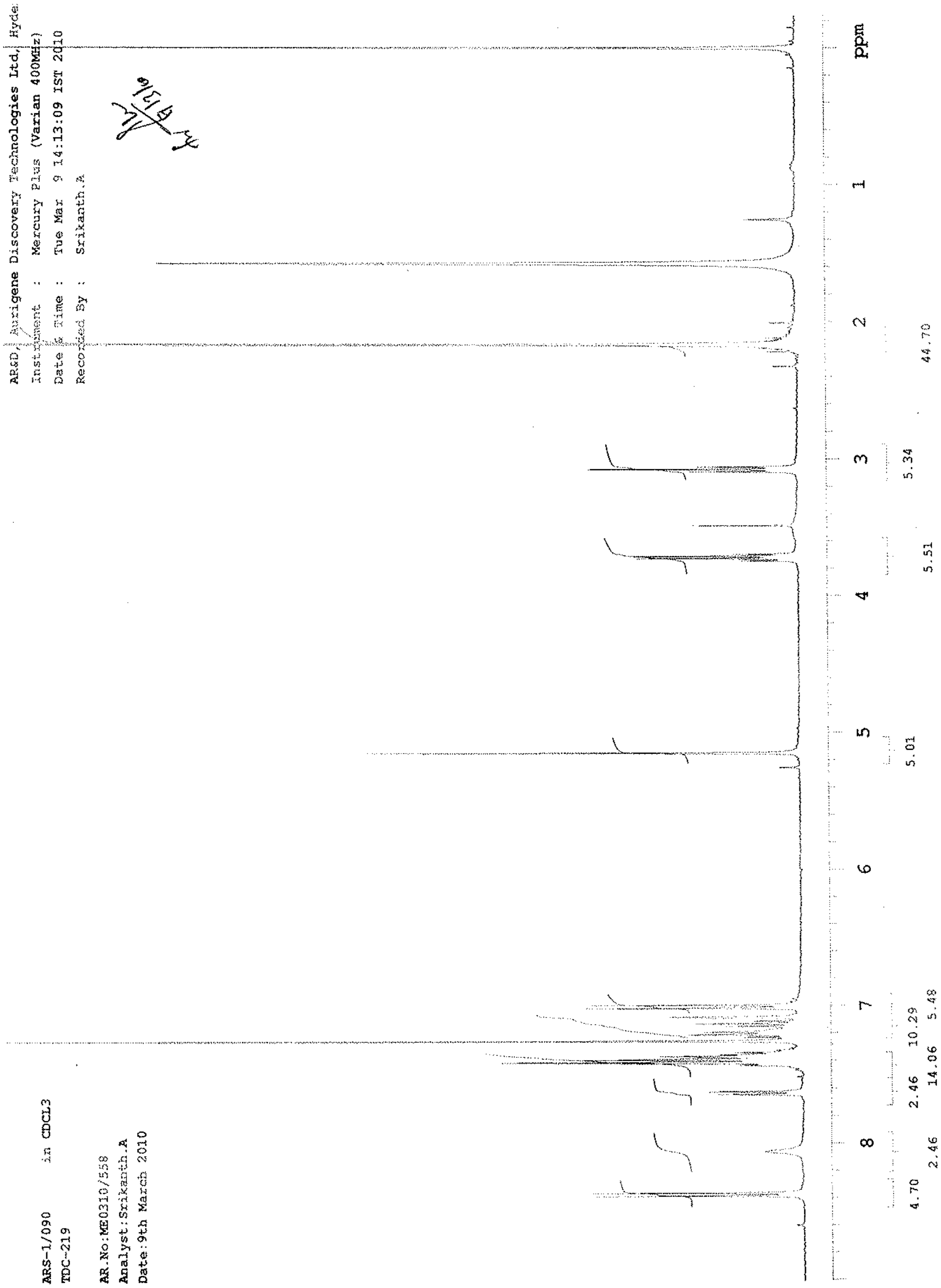

^{13}C NMR of *N*-(2-(1*H*-indol-3-yl)ethyl)-2-(4-(benzyloxy)phenyl)-2-oxoacetamide (**9h**):

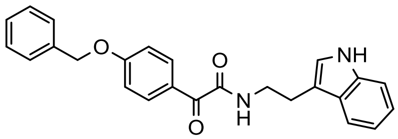

9h

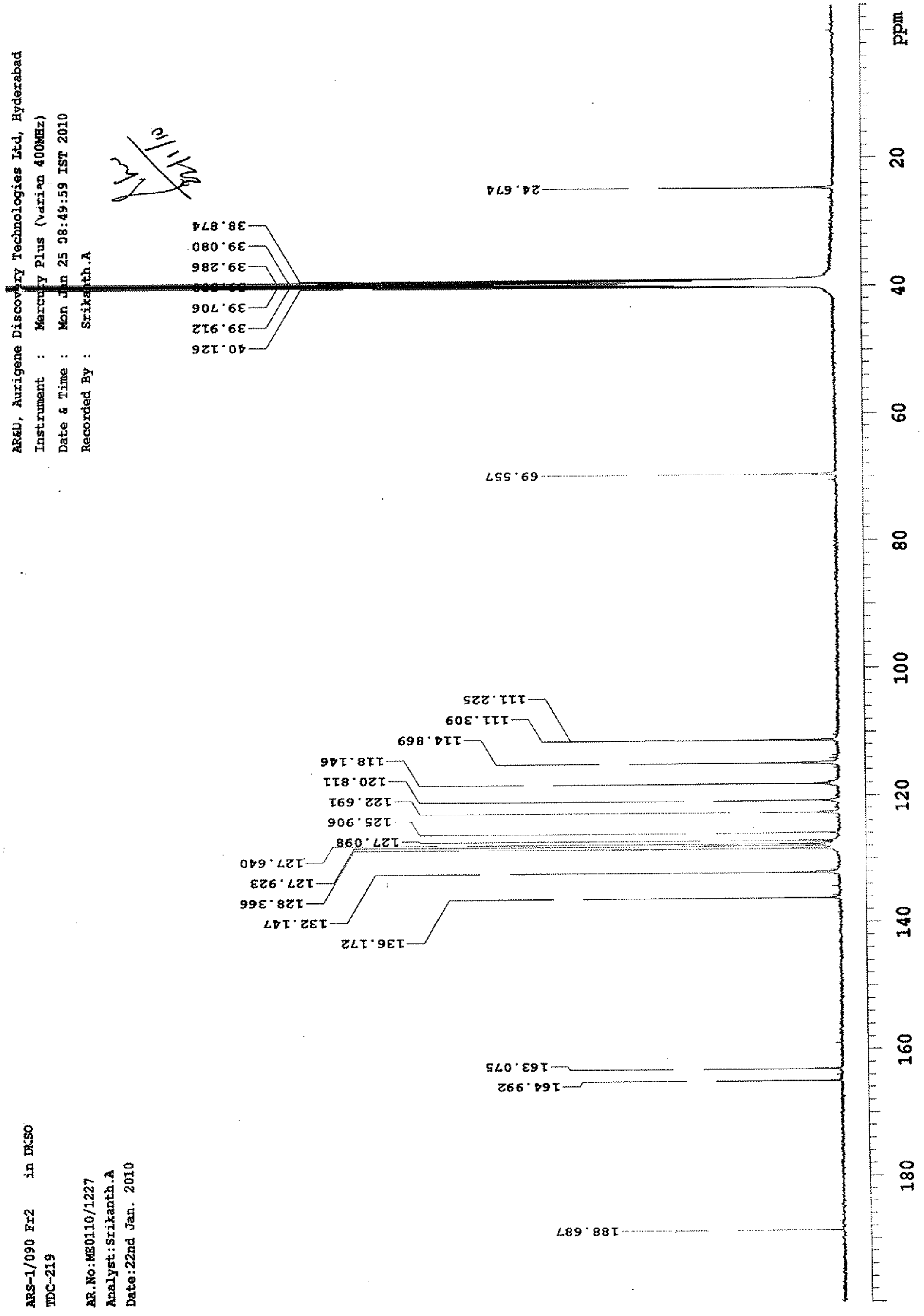

Mass spectrum of *N*-(2-(1*H*-indol-3-yl)ethyl)-2-(4-(benzyloxy)phenyl)-2-oxoacetamide (**9h**):

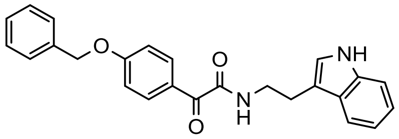

9h

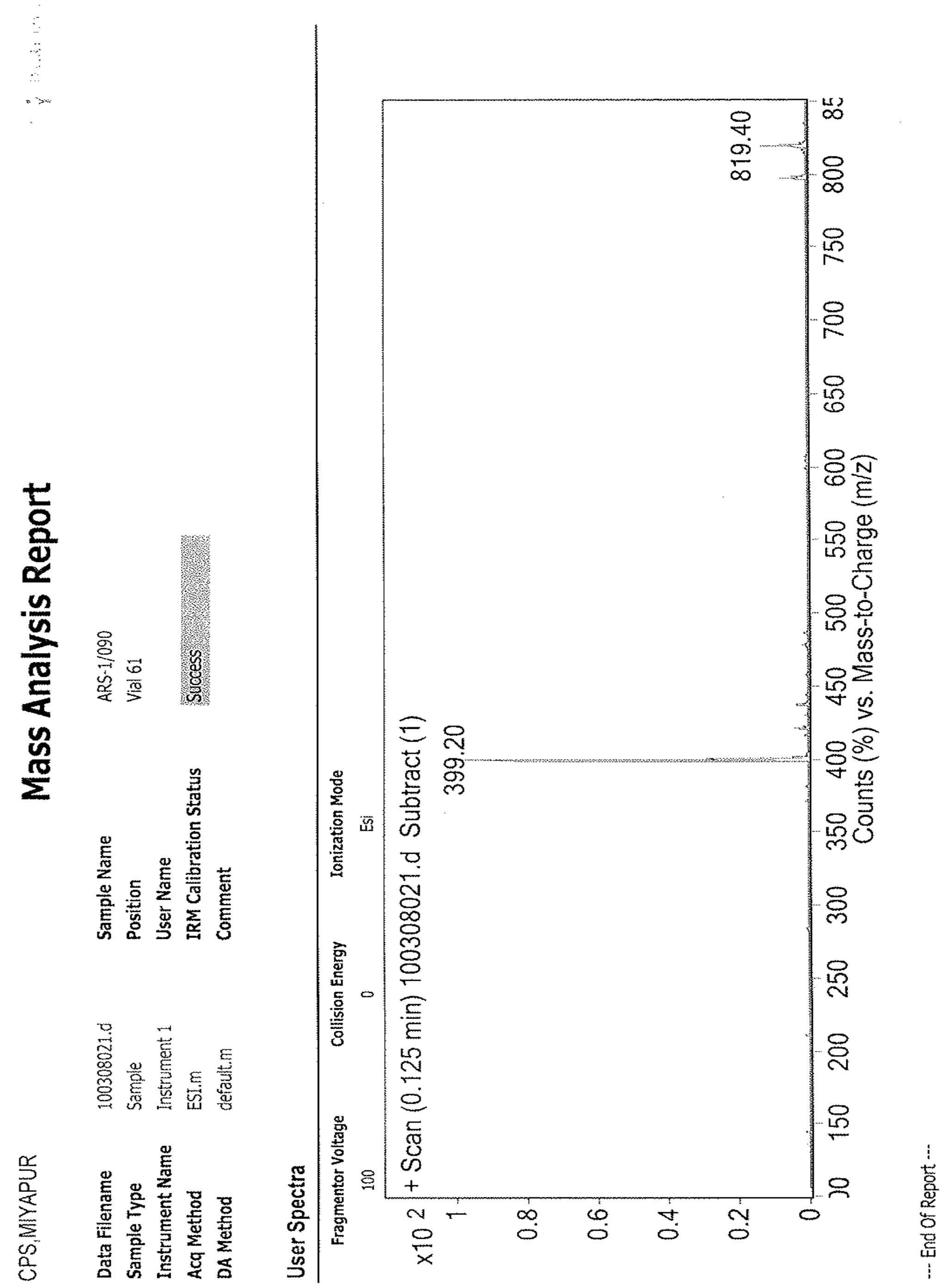

IR spectrum of *N*-(2-(1*H*-indol-3-yl)ethyl)-2-(4-(benzyloxy)phenyl)-2-oxoacetamide (**9h**):

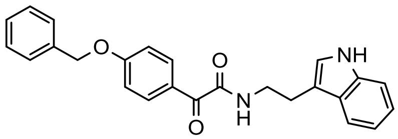

9h

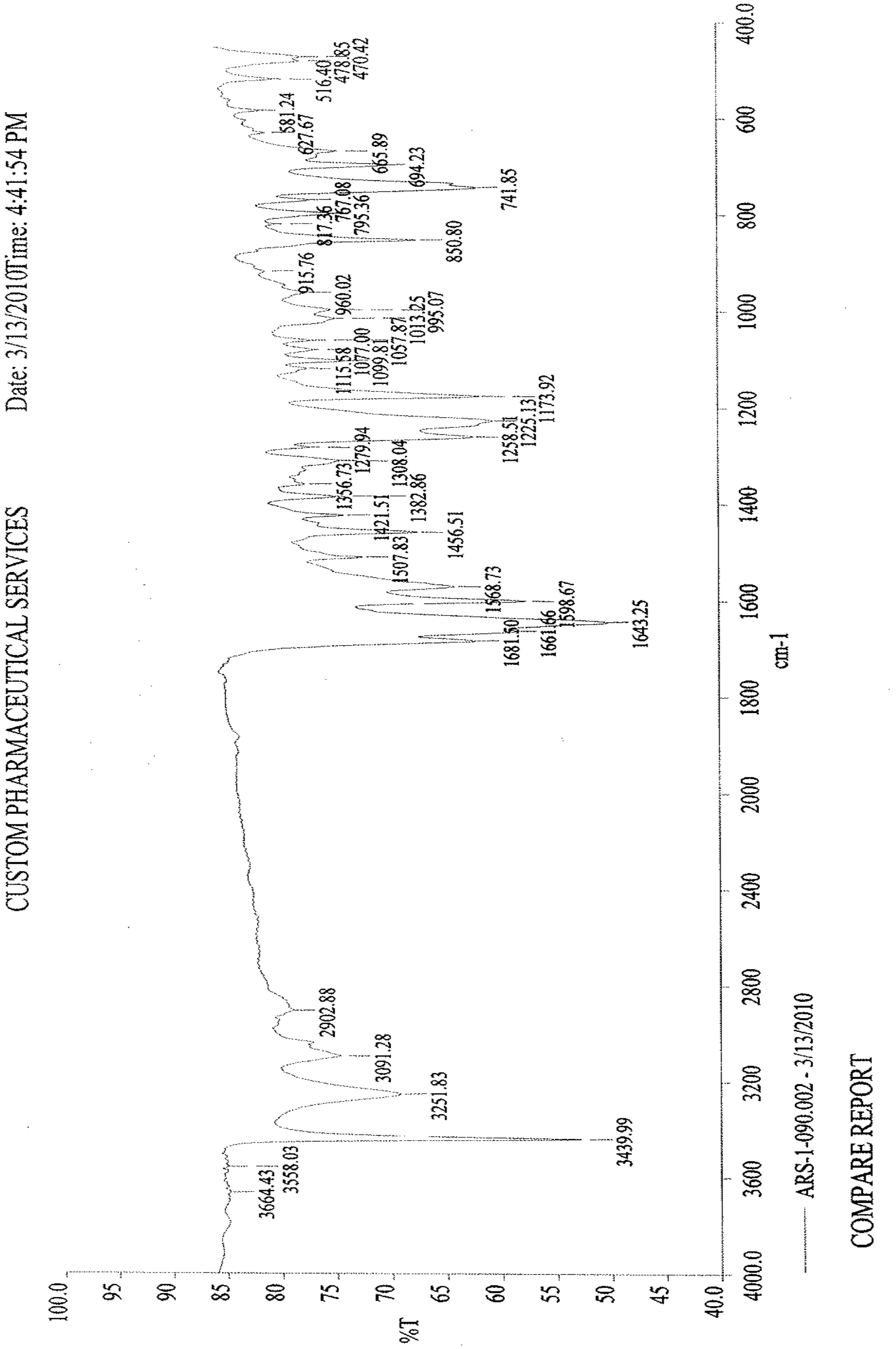

^{1}H NMR of *N*-(2-(1*H*-indol-3-yl)ethyl)-2-([1,1'-biphenyl]-4-yl)-2-oxoacetamide (**9i**):

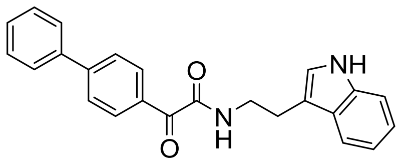

9i

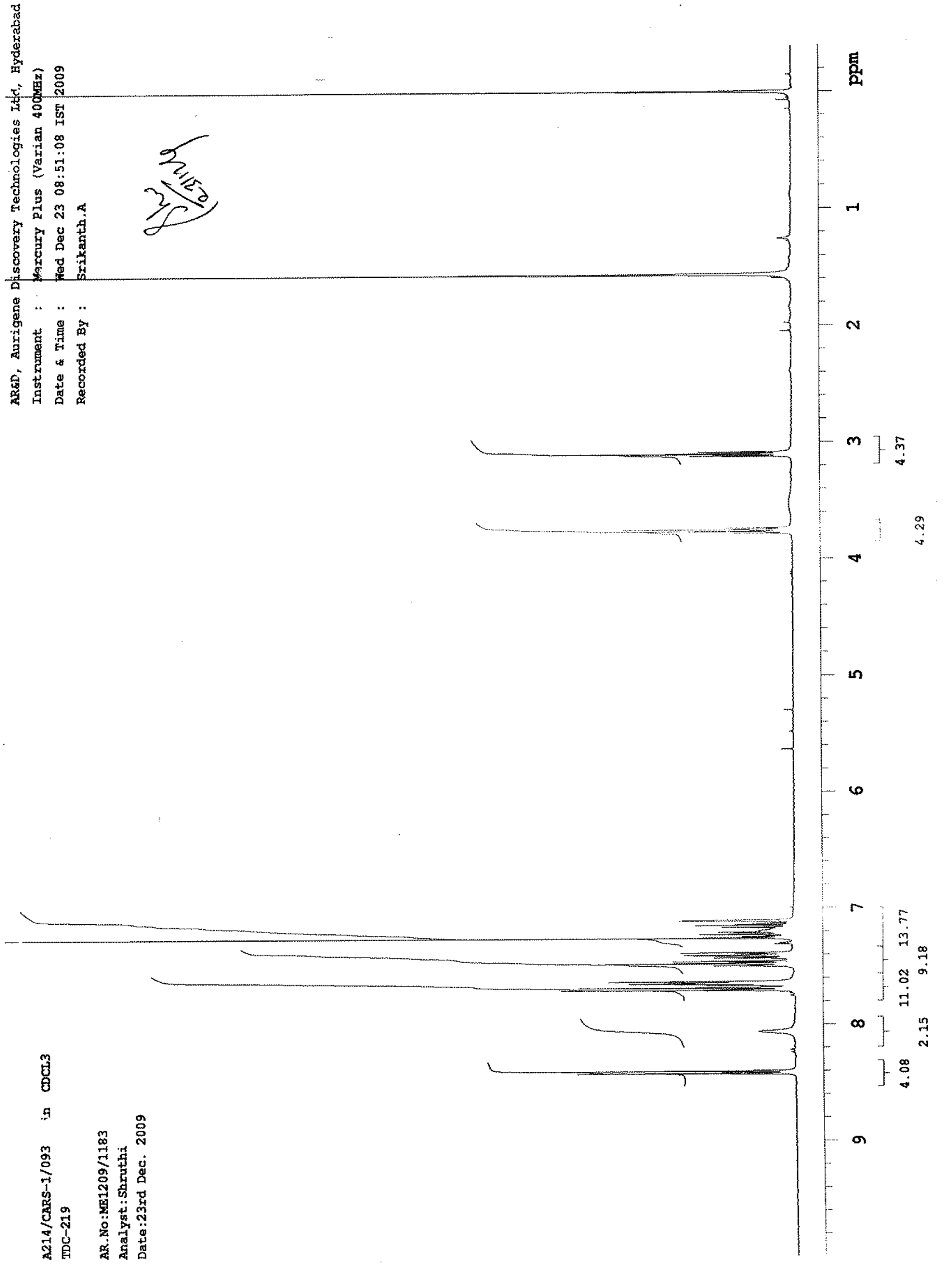

^{13}C NMR of *N*-(2-(1*H*-indol-3-yl)ethyl)-2-([1,1'-biphenyl]-4-yl)-2-oxoacetamide (**9i**):

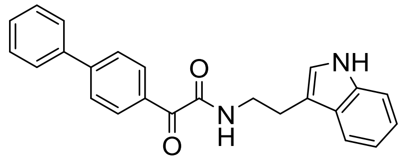

9i

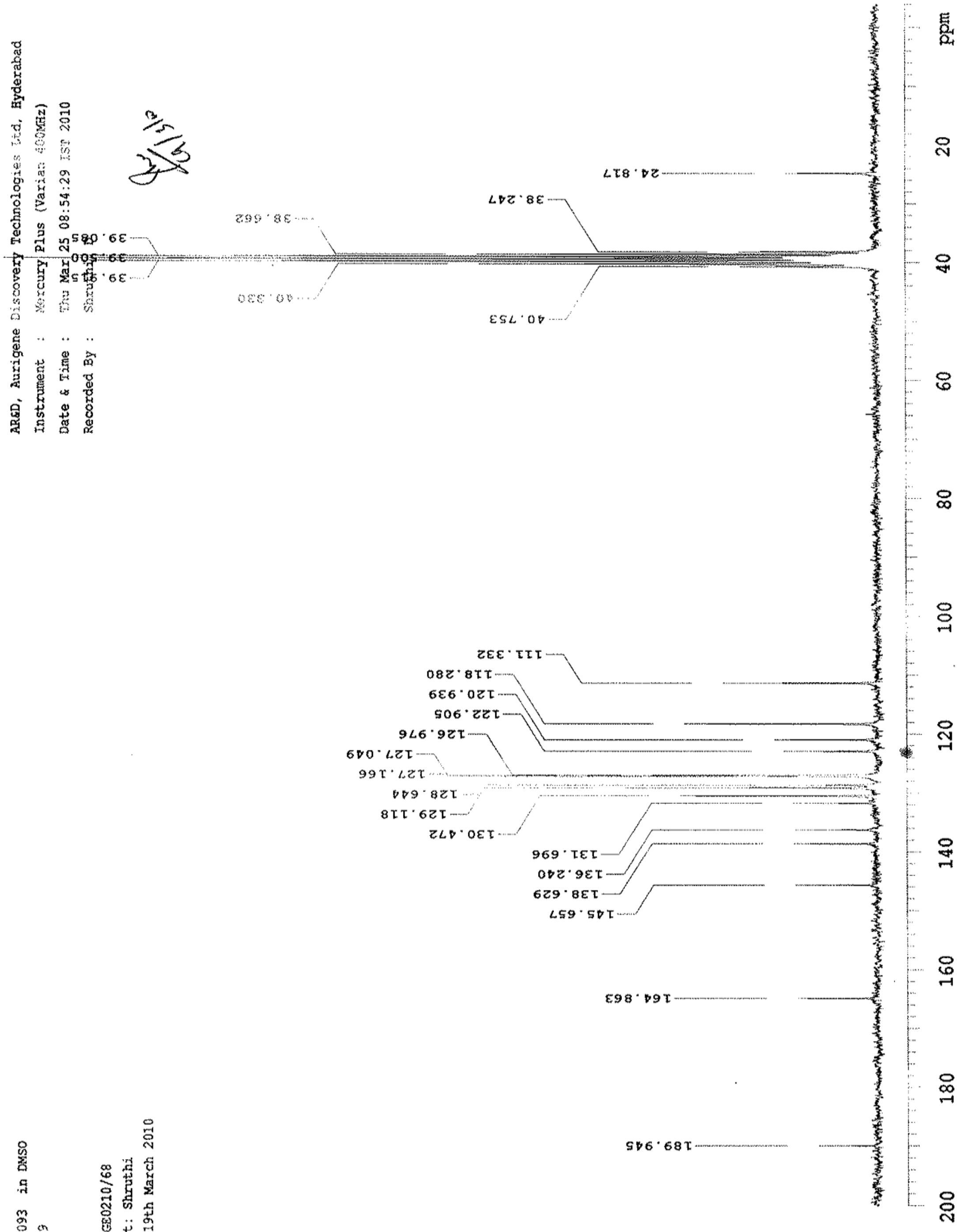

Mass spectrum of *N*-(2-(1*H*-indol-3-yl)ethyl)-2-([1,1'-biphenyl]-4-yl)-2-oxoacetamide (**9i**):

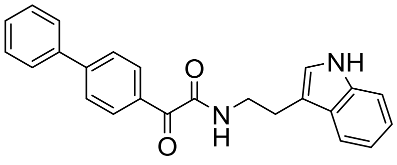

9i

CPS,MIYAPUR

Mass Analysis Report

**Data Filename**	091222014.d	**Sample Name**	A214/CARS-1/093
**Sample Type**	Sample	**Position**	Vial 44
**Instrument Name**	Instrument 1	**User Name**	
**Acq Method**	ESI.m	**IRM Calibration Status**	Success
**DA Method**	default.m	**Comment**	

User Spectra

Fragmentor Voltage	Collision Energy	Ionization Mode
135	0	Esi

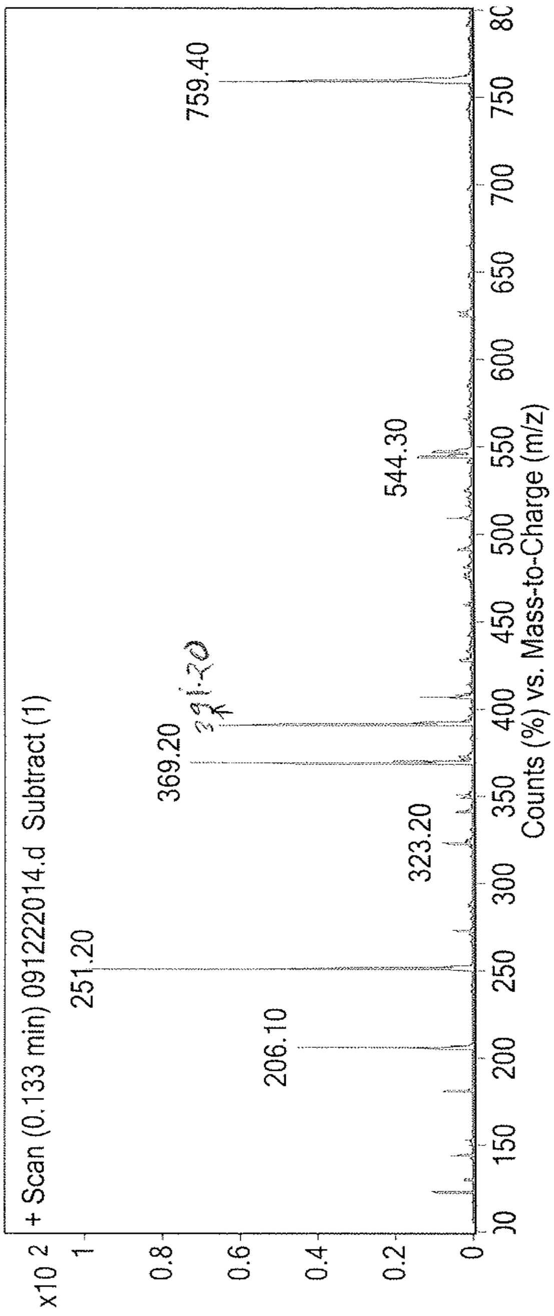

--- End Of Report ---

IR spectrum of *N*-(2-(1*H*-indol-3-yl)ethyl)-2-([1,1'-biphenyl]-4-yl)-2-oxoacetamide (**9i**):

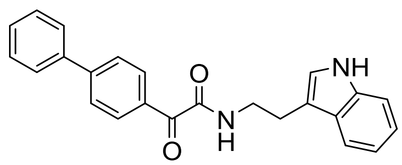

9i

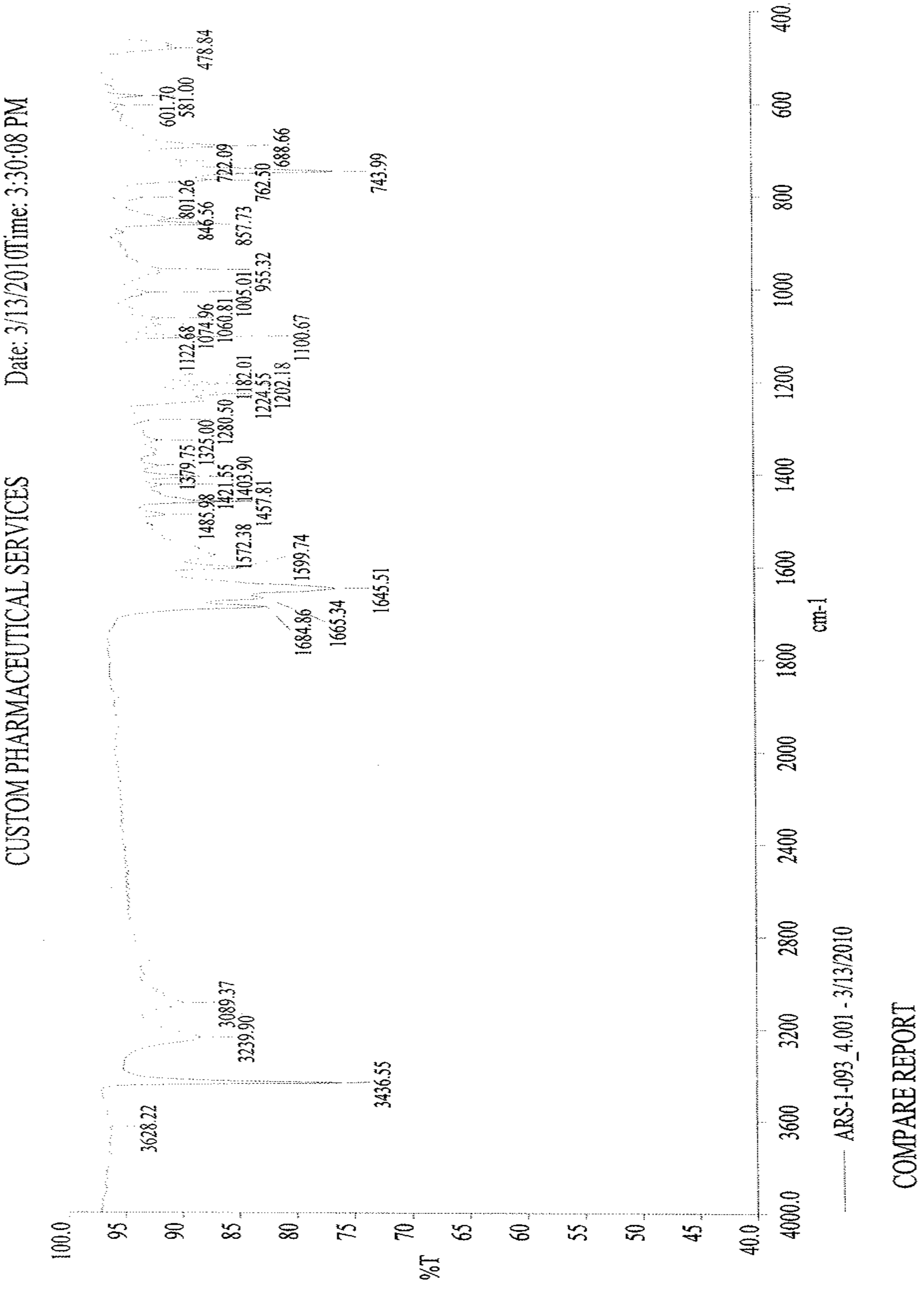

^{1}H NMR of *N*-(2-(1*H*-indol-3-yl)ethyl)-2-oxo-2-(thiophen-2-yl)acetamide (**9j**):

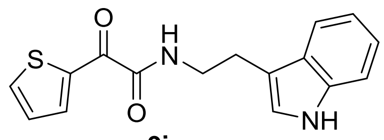

9j

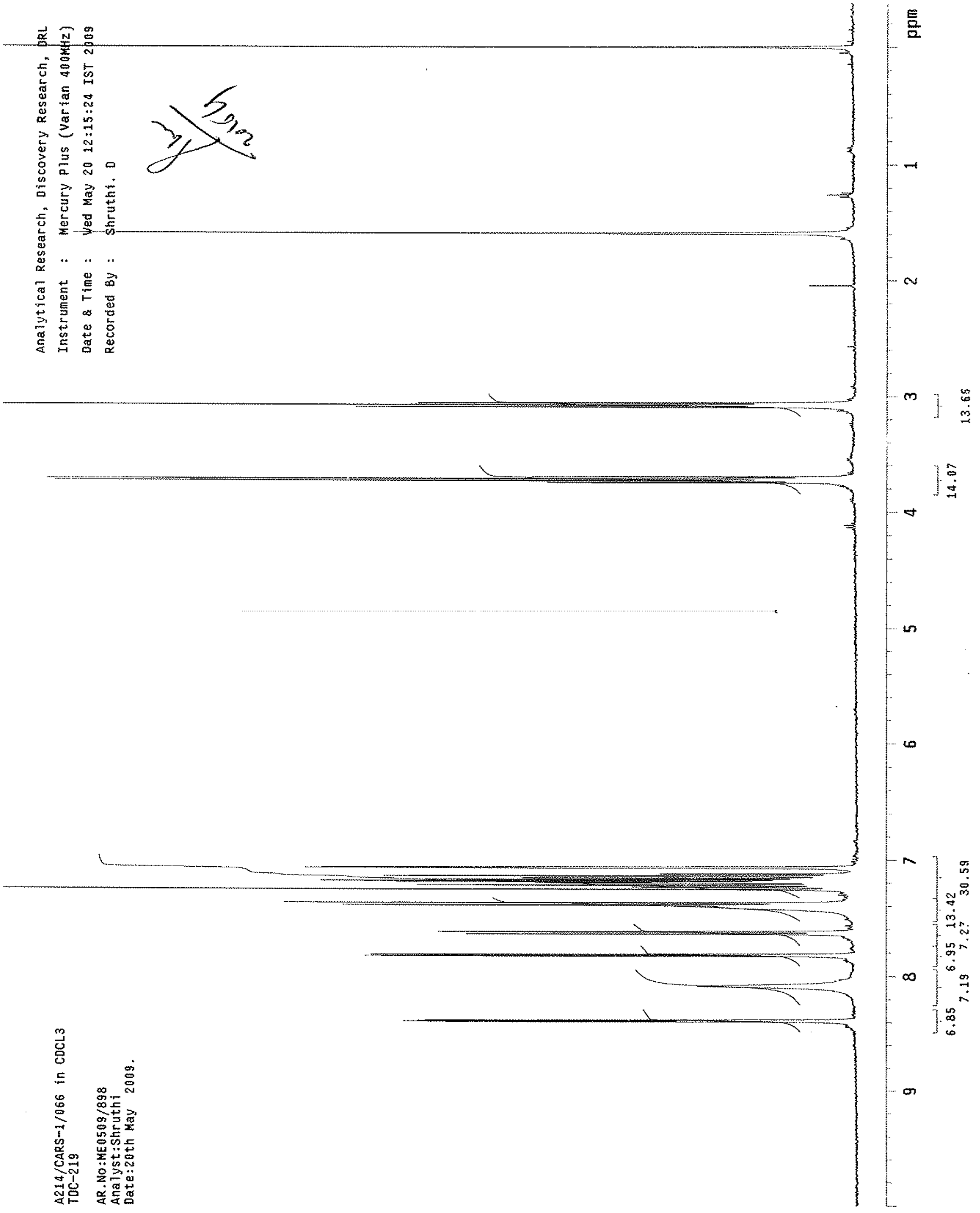

^{13}C NMR of *N*-(2-(1*H*-indol-3-yl)ethyl)-2-oxo-2-(thiophen-2-yl)acetamide (**9j**):

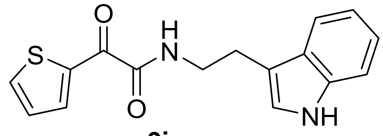

9j

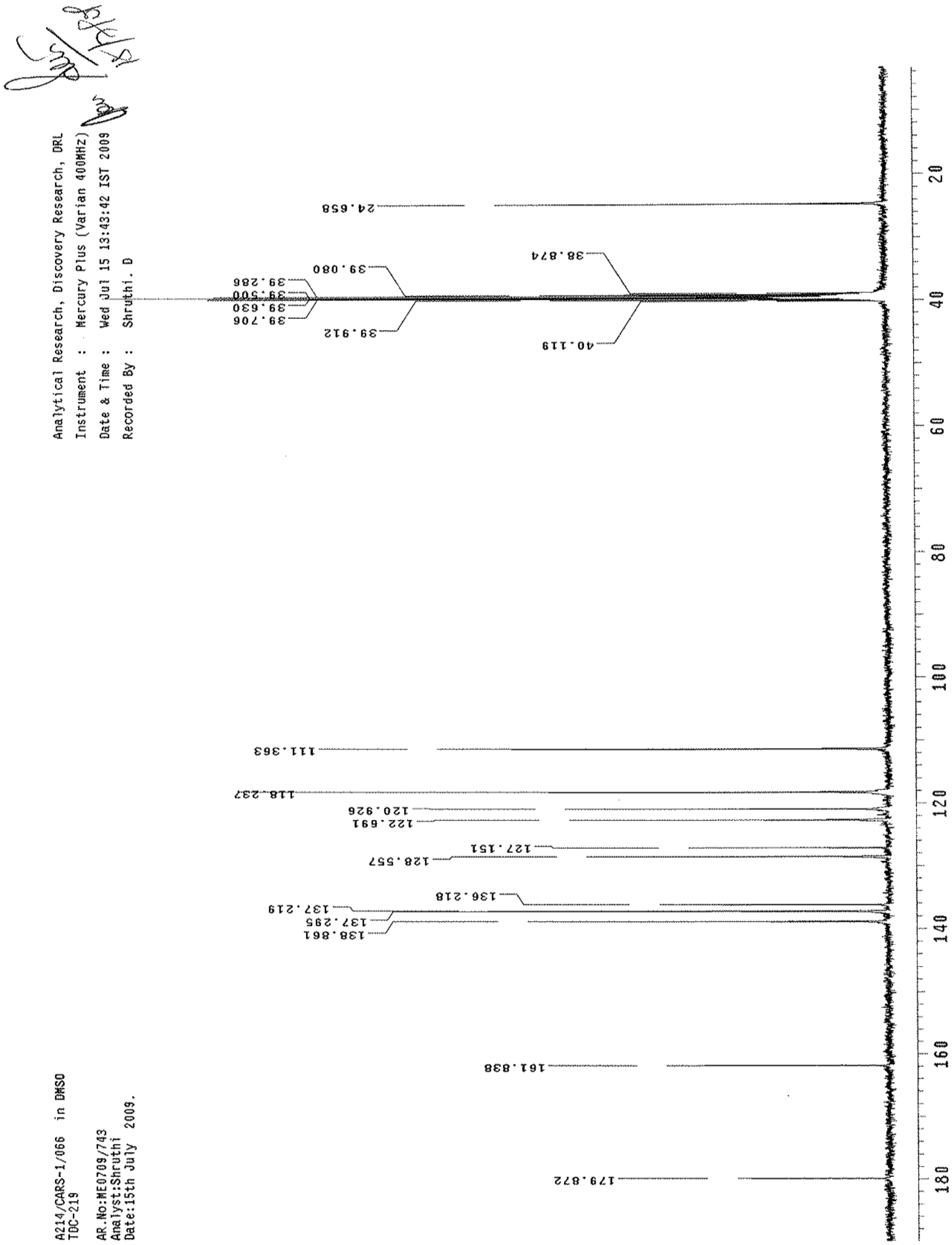

Mass spectrum of *N*-(2-(1*H*-indol-3-yl)ethyl)-2-oxo-2-(thiophen-2-yl)acetamide (**9j**):

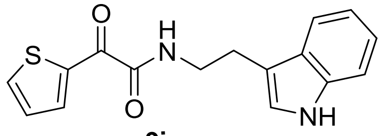

9j

CPS,MIYAPUR

Mass Analysis Report

**Data Filename**	090520004.d	**Sample Name**	A214/CARS-1/066 FR-1
**Sample Type**	Sample	**Position**	Vial 54
**Instrument Name**	Instrument 1	**User Name**	
**Acq Method**		**IRM Calibration Status**	Success
**DA Method**	Quant Process.m	**Comment**	

User Spectra

Fragmentor Voltage	Collision Energy	Ionization Mode
135	0	Esi

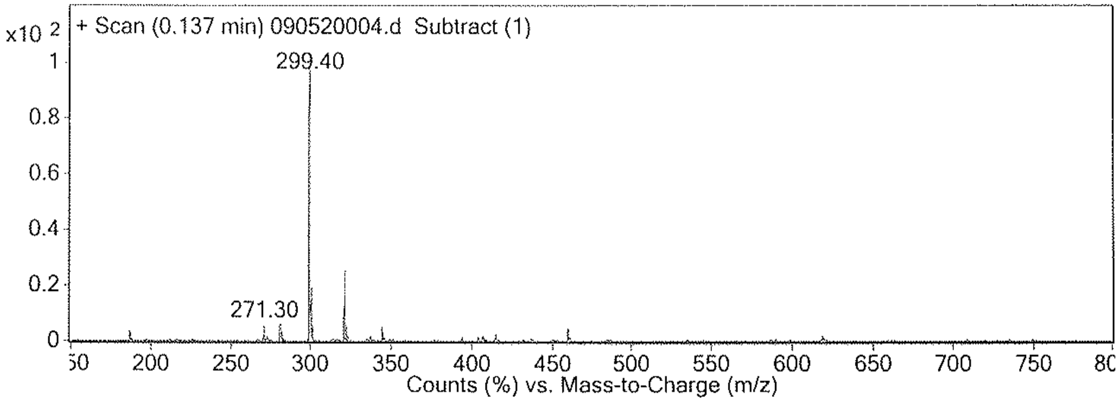

--- End Of Report ---

IR spectrum of *N*-(2-(1*H*-indol-3-yl)ethyl)-2-oxo-2-(thiophen-2-yl)acetamide (**9j**):

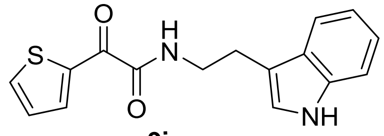

9j

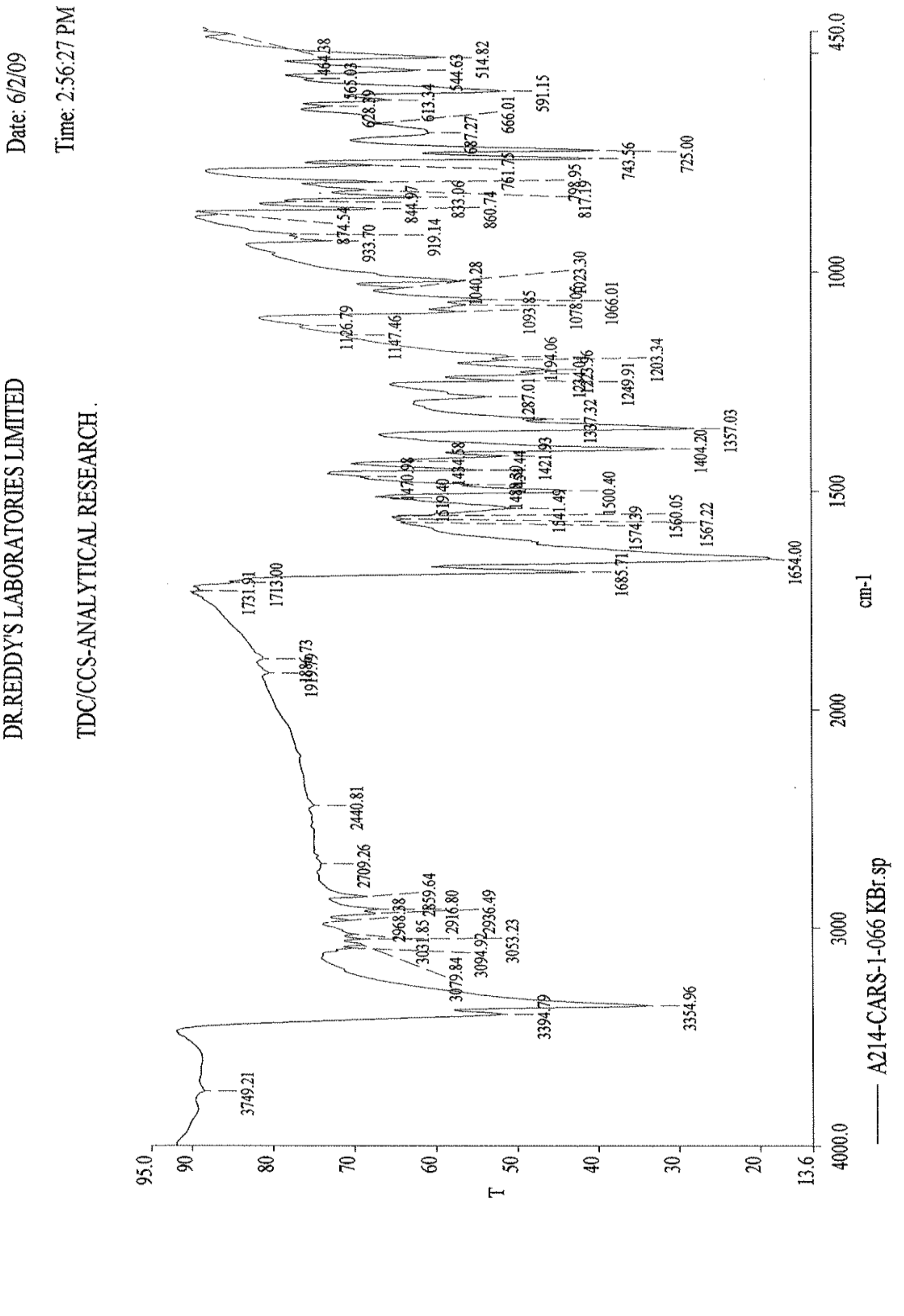

^{1}H NMR of (2,9-dihydro-1*H*-pyrido[3,4-b]indol-1-yl)(phenyl)methanone (**7a**):

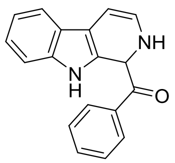

7a

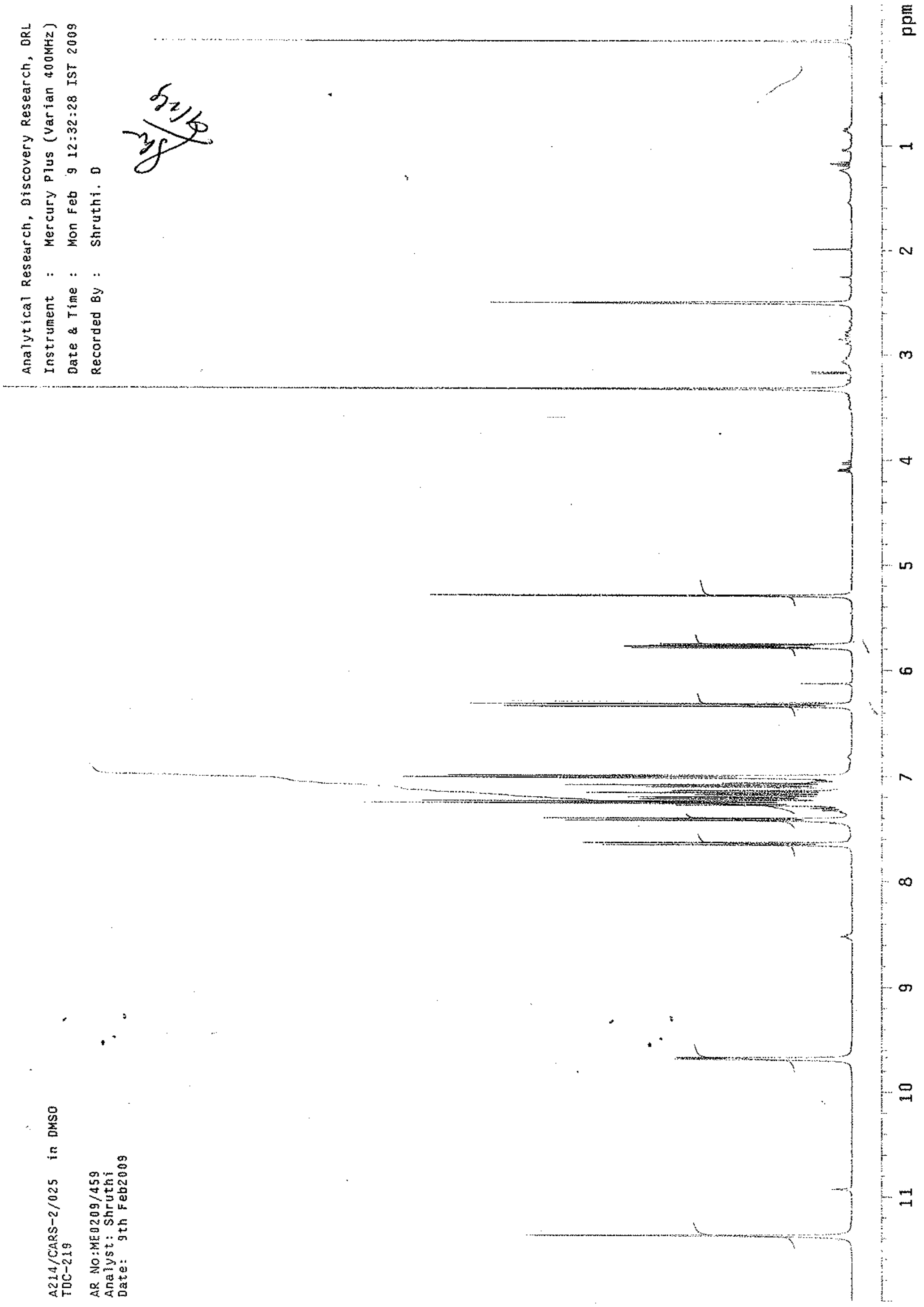

^{13}C NMR of (2,9-dihydro-1*H*-pyrido[3,4-b]indol-1-yl)(phenyl)methanone (**7a**):

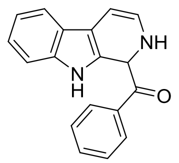

7a

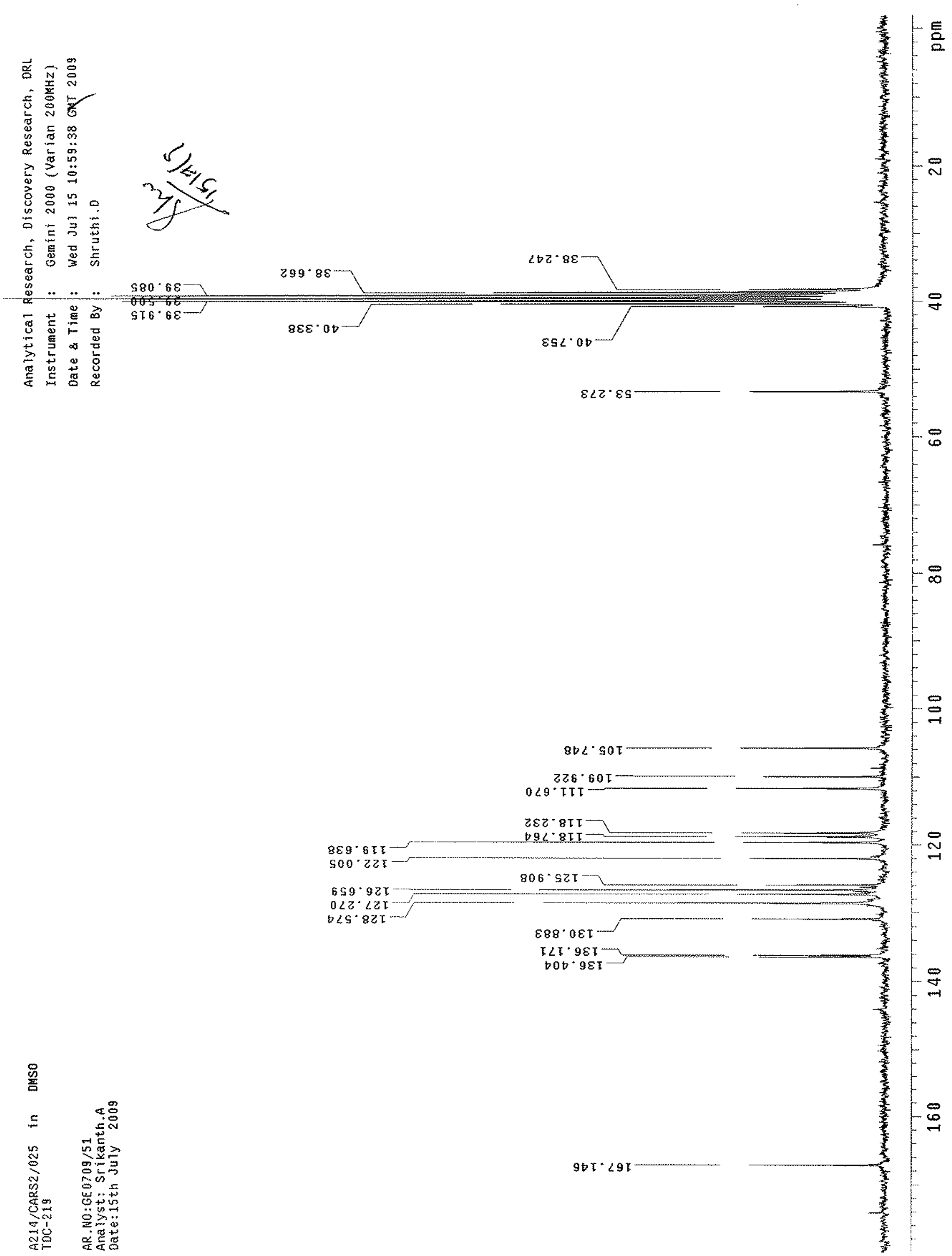

COSY spectrum of (2,9-dihydro-1*H*-pyrido[3,4-b]indol-1-yl)(phenyl)methanone (**7a**):

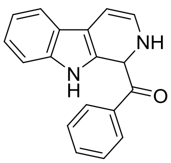

7a

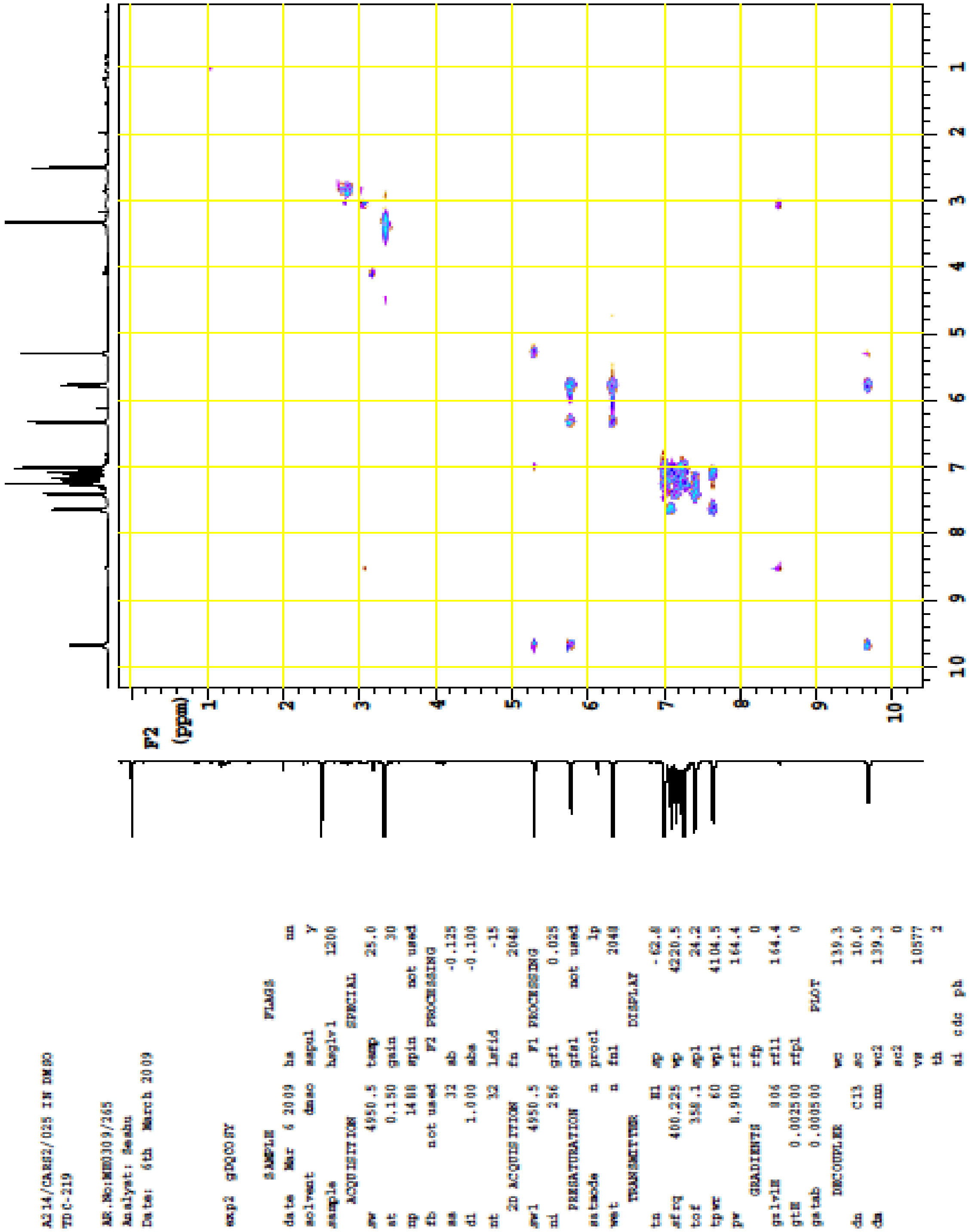

HSQC spectrum of (2,9-dihydro-1*H*-pyrido[3,4-b]indol-1-yl)(phenyl)methanone (**7a**):

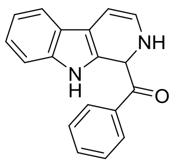

7a

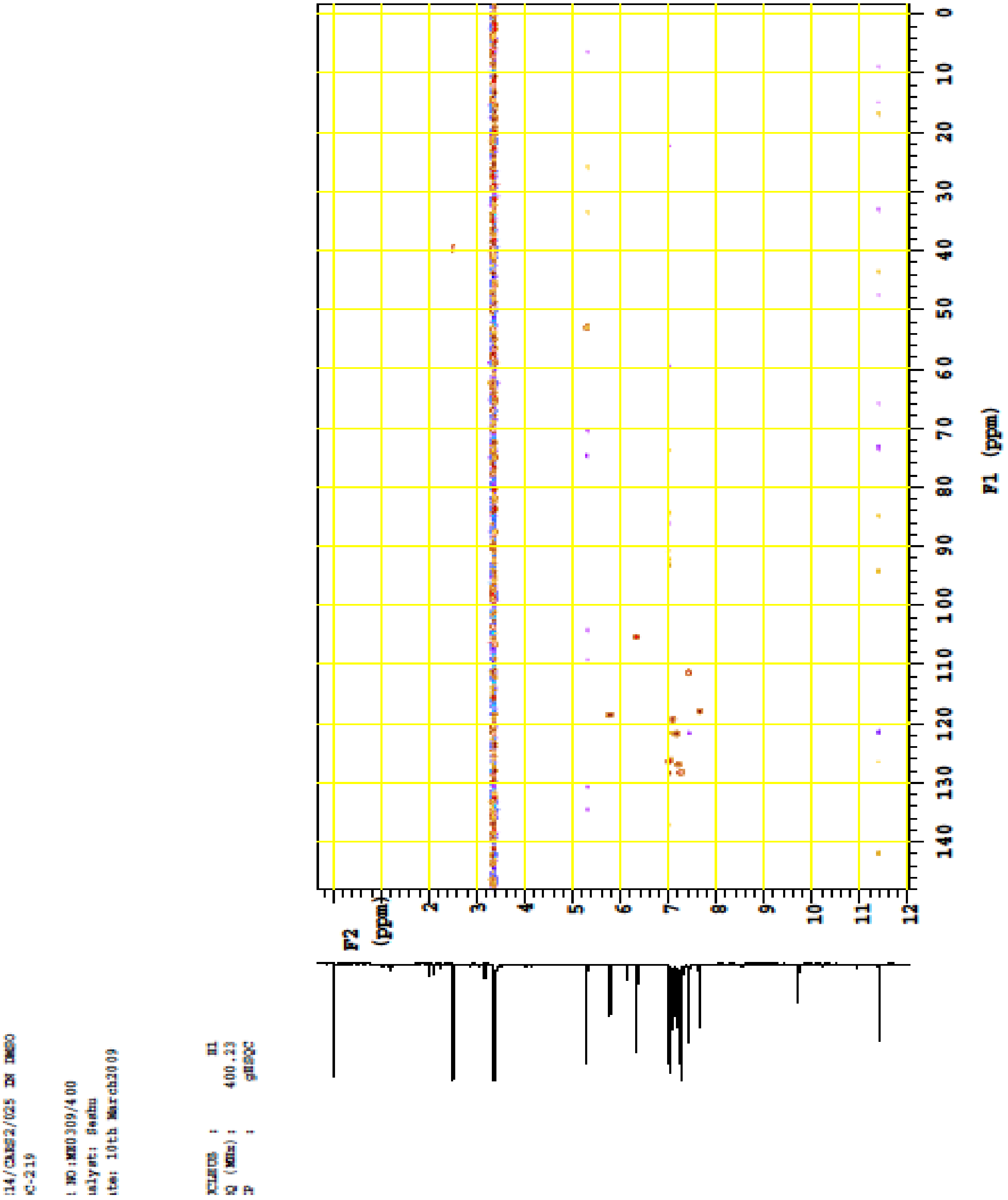

Mass spectrum of (2,9-dihydro-1*H*-pyrido[3,4-b]indol-1-yl)(phenyl)methanone (**7a**):

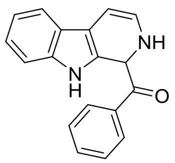

7a

CPS,MIYAPUR

Mass Analysis Report

**Data Filename**	090119006.d	**Sample Name**	A214/CARS-2/016 RC
**Sample Type**	Sample	**Position**	Vial 5
**Instrument Name**	Instrument 1	**User Name**	
**Acq Method**	ESI.m	**IRM Calibration Status**	Success
**DA Method**	DA.m	**Comment**	

User Spectra

Fragmentor Voltage	Collision Energy	Ionization Mode
135	0	Esi

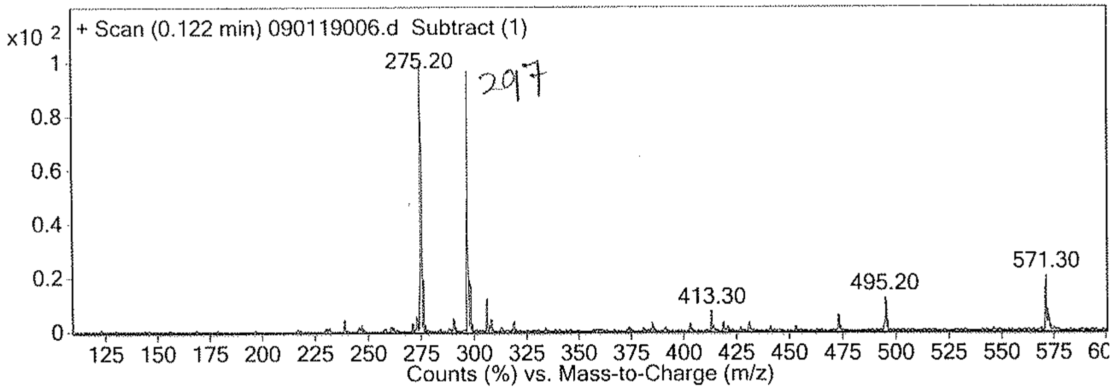

--- End Of Report ---

HRMS of (2,9-dihydro-1*H*-pyrido[3,4-b]indol-1-yl)(phenyl)methanone (**7a**):

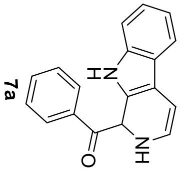

7a

Elemental Composition Report — **Page 1**

Single Mass Analysis
Tolerance = 5.0 PPM / DBE: min = -1.5, max = 80.0
Element prediction: Off
Number of isotope peaks used for i-FIT = 3

Monoisotopic Mass, Even Electron Ions
96 formula(e) evaluated with 1 results within limits (up to 4 best isotopic matches for each mass)
Elements Used:
C: 0-30 H: 0-30 N: 0-5 O: 0-5
9a
UT1212_148 10 (0.366) Cm (10:13-(1:4+22:28)x0.010)

Gajanan
1: TOF MS ES+
7.37e+003

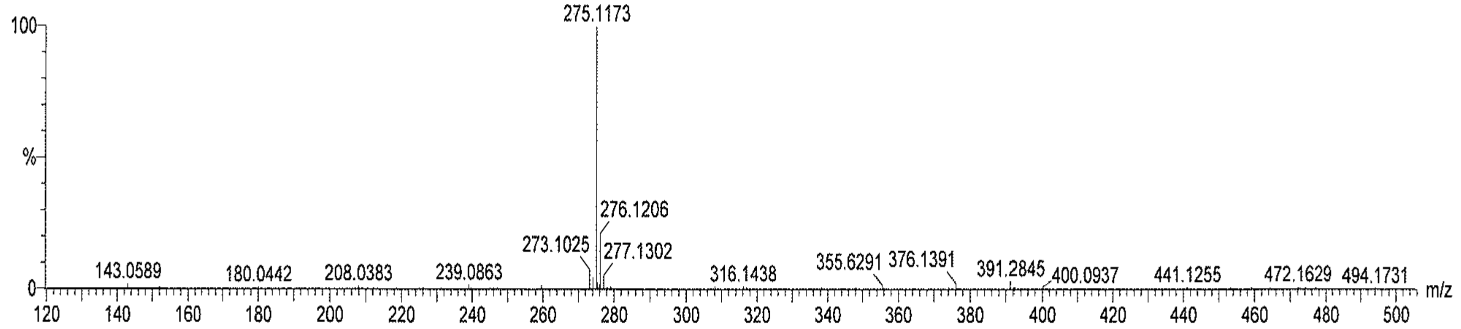

Minimum:				-1.5		
Maximum:		5.0	5.0	80.0		
Mass	Calc. Mass	mDa	PPM	DBE	i-FIT	Formula
275.1173	275.1184	-1.1	-4.0	12.5	54.7	C18 H15 N2 O

^{1}H NMR of (1-hydroxy-2,3,4,9-tetrahydro-1*H*-pyrido[3,4-b]indol-1-yl)(phenyl)methanone (**8a**):

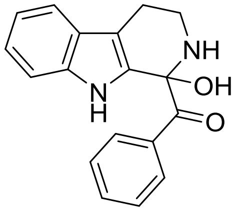

8a

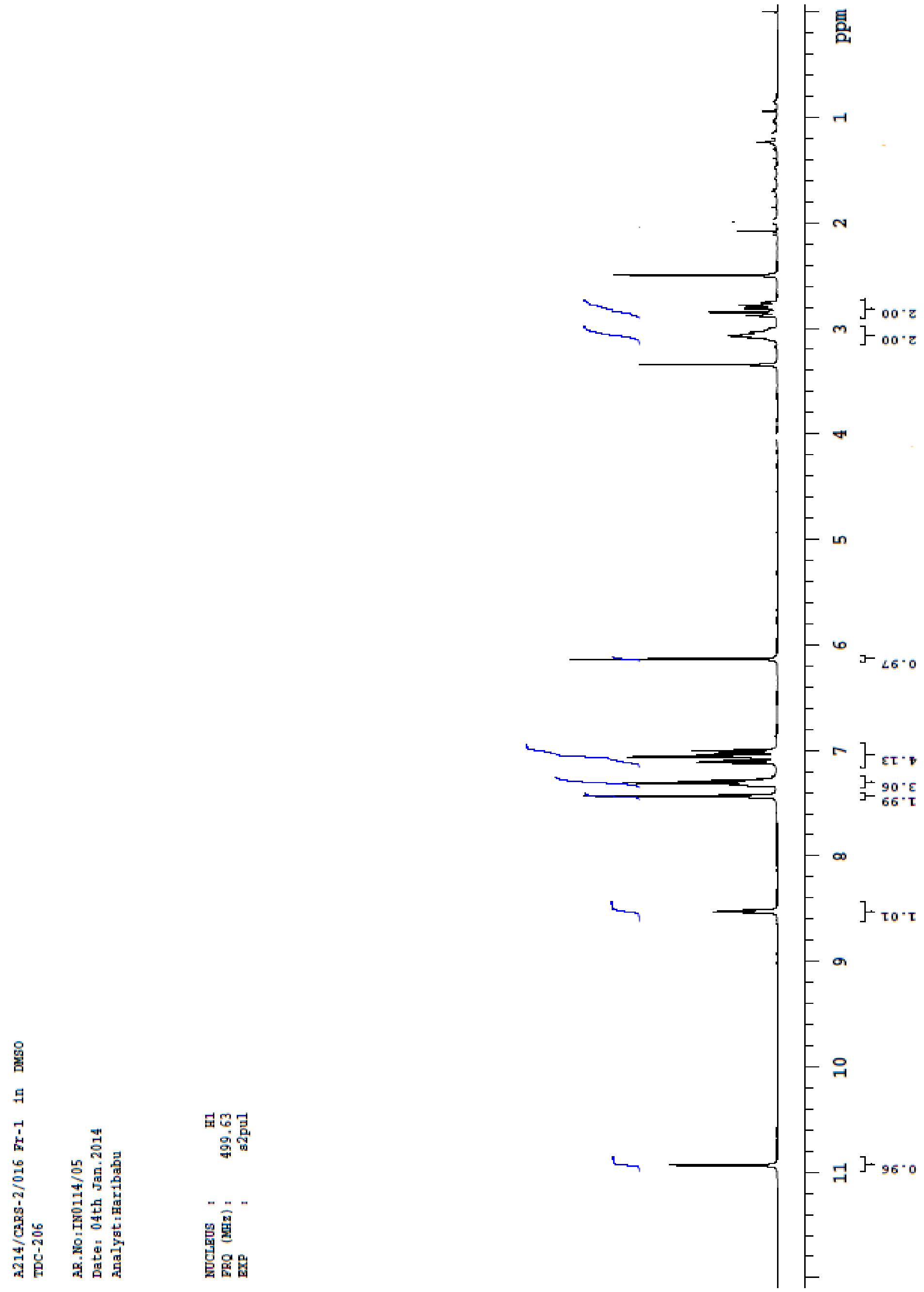

^{1}H NMR D_2O exchange of (1-hydroxy-2,3,4,9-tetrahydro-1*H*-pyrido[3,4-b]indol-1-yl)(phenyl)methanone (**8a**):

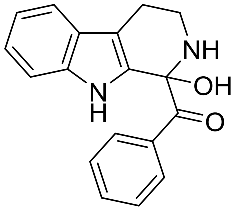

8a

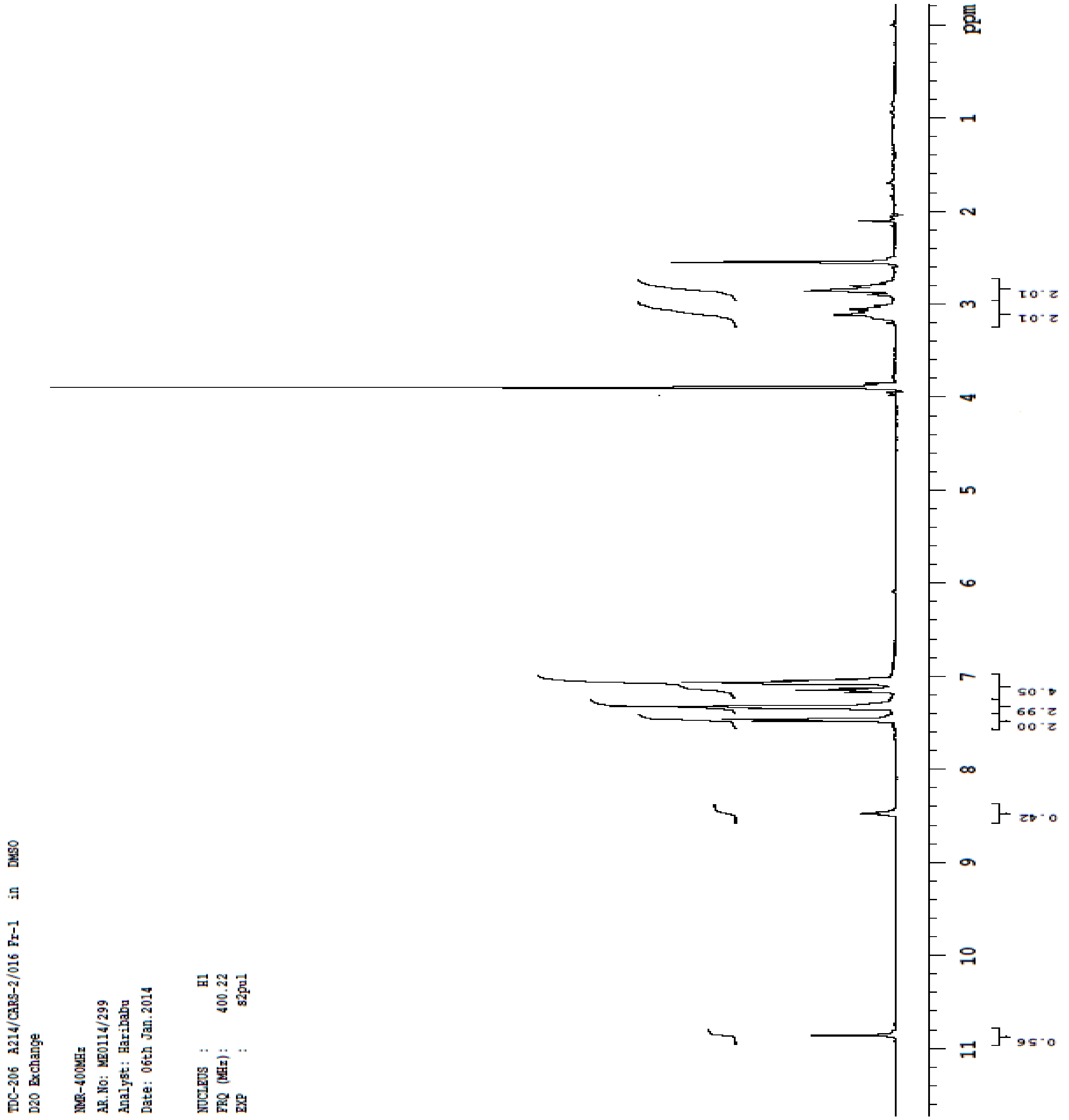

COSY spectrum of(1-hydroxy-2,3,4,9-tetrahydro-1*H*-pyrido[3,4-b]indol-1-yl)(phenyl)methanone (**8a**):

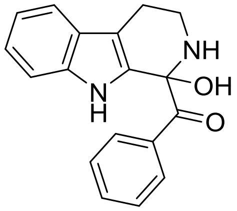

8a

A214/CARS-2/016 Fr-1 in DMSO
TDC-206

AR.No:IN0114/06
Date: 04th Jan.2014
Analyst:Haribabu

exp2 gDQCOSY

SAMPLE		FLAGS	
date	Jan 4 2014	hs	nn
solvent	DMSO	sspul	y
sample		hsglvl	4237
ACQUISITION		SPECIAL	
sw	7107.3	temp	25.0
at	0.144	gain	16
np	2048	spin	not used
fb	not used	F2 PROCESSING	
ss	32	sb	-0.108
d1	1.000	sbs	-0.072
nt	8	lsfid	-22
2D ACQUISITION		fn	2048
sw1	7107.3	F1 PROCESSING	
ni	400	sb1	-0.056
TRANSMITTER		sbs1	-0.025
tn	H1	gf1	0.024
sfrq	499.629	gfs1	not used
tof	530.1	proc1	lp
tpwr	58	fn1	2048
pw	7.200	DISPLAY	
GRADIENTS		sp	-90.5
gzlvl1	3177.5	wp	5989.9
gt1	0.002500	sp1	-21.1
gzlvl2	6355	wp1	5913.5
gt2	0.002500	rfl	1776.8
gstab	0.000500	rfp	1249.1
DECOUPLER		rfl1	1776.8
dn	C13	rfp1	1249.1
dm	nnn	PLOT	
		wc	138.9
		sc	10.0
		wc2	138.9
		sc2	0
		vs	66
		th	2
		ai cdc ph	

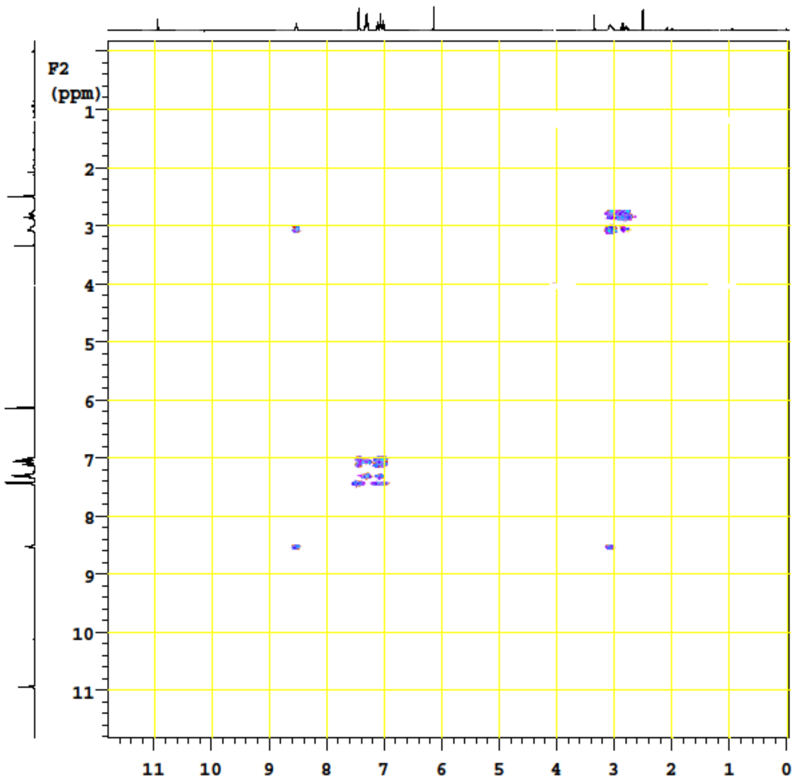

HSQC spectrum of(1-hydroxy-2,3,4,9-tetrahydro-1*H*-pyrido[3,4-b]indol-1-yl)(phenyl)methanone (**8a**):

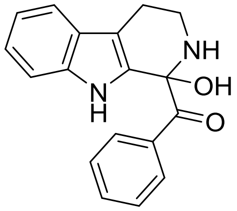

8a

A214/CARS-2/016 Fr-1 in DMSO
TDC-206

AR.No:IN0114/07
Date: 04th Jan.2014
Analyst:Haribabu

NUCLEUS : H1
FRQ (MHz): 499.63
EXP : gHSQC

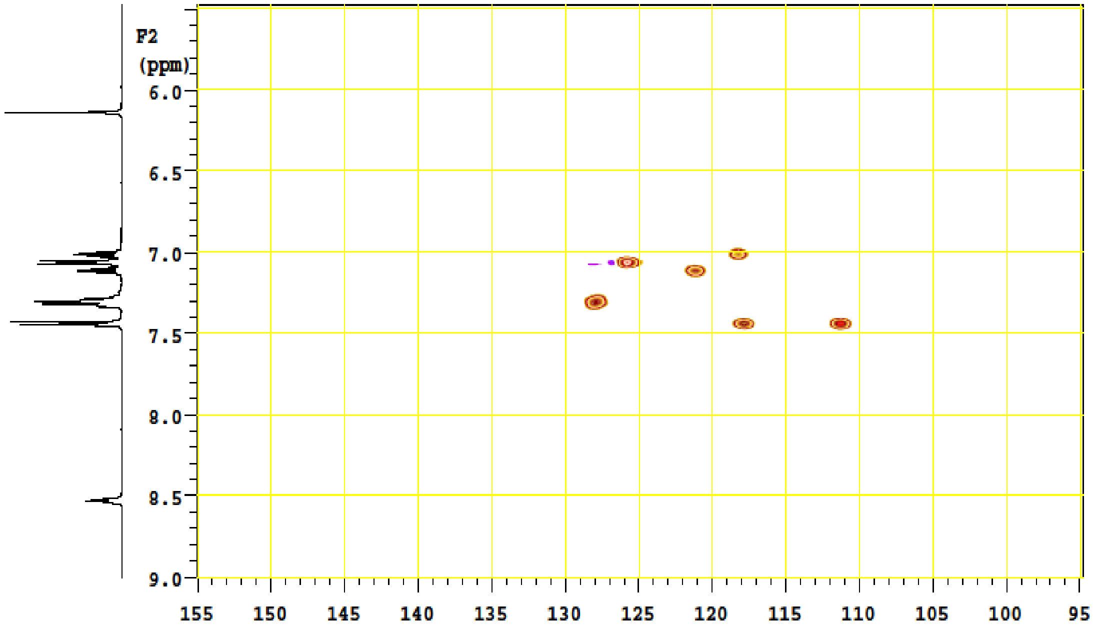

NOESY spectrum of(1-hydroxy-2,3,4,9-tetrahydro-1*H*-pyrido[3,4-b]indol-1-yl)(phenyl)methanone (**8a**):

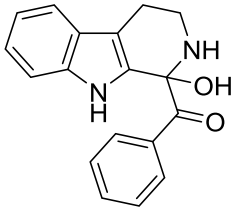

8a

```
A214/CARS-2/016 Fr-1  in  DMSO
TDC-206

AR.No:IN0114/09
Date: 04th Jan.2014
Analyst:Haribabu


exp2  NOESY

      SAMPLE                 FLAGS
date   Jan  4 2014  hs                  n
solvent       DMSO  sspul               y
sample              PFGflg              y
   ACQUISITION      hsglvl           4237
sw          7107.3        SPECIAL
at           0.144  temp             25.0
np            2048  gain               14
fb        not used  spin         not used
ss              32     F2 PROCESSING
d1           1.000  gf              0.067
nt              16  gfs          not used
  2D ACQUISITION    fn               2048
sw1         7107.3     F1 PROCESSING
ni             400  gf1             0.024
   TRANSMITTER      gfs1         not used
tn              H1  proc1              lp
sfrq       499.629  fn1              2048
tof          530.1        DISPLAY
tpwr            58  sp              -55.8
pw           7.200  wp             5934.3
      NOESY         sp1            -104.4
mix          0.600  wp1            5976.0
  PRESATURATION     rfl            1776.8
satmode       nnnn  rfp            1249.1
satpwr           0  rfl1           1776.8
satdly           0  rfp1           1249.1
satfrq           0          PLOT
    DECOUPLER       wc              139.3
dn             C13  sc               10.0
dm             nnn  wc2             139.3
                    sc2                 0
                    vs               4813
                    th                  2
                    ai    ph
```

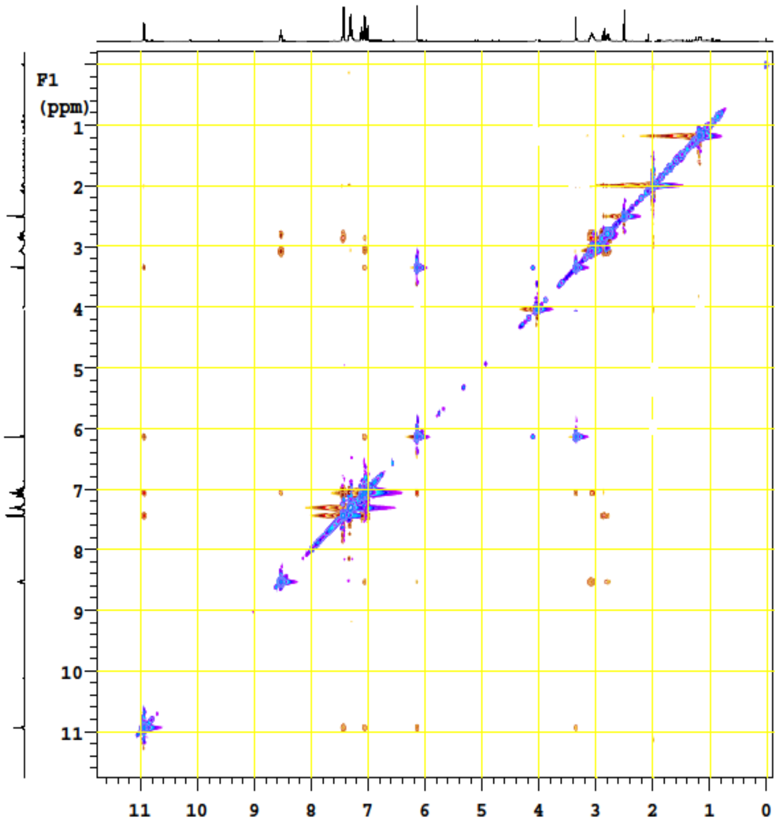

HRMS spectrum of of(1-hydroxy-2,3,4,9-tetrahydro-1*H*-pyrido[3,4-b]indol-1-yl)(phenyl)methanone (**8a**):

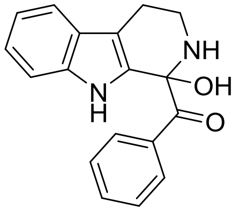

8a

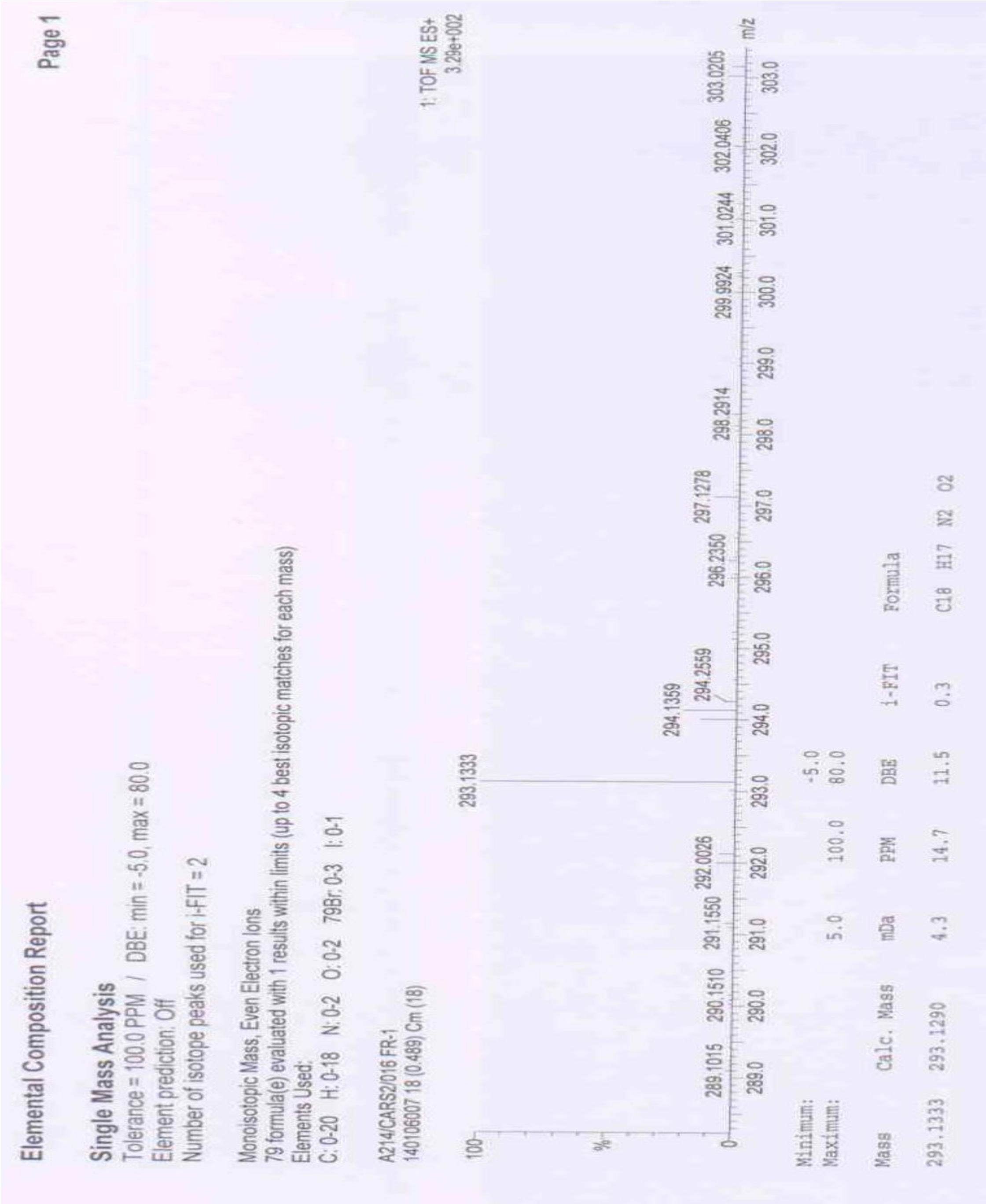

Mass spectrum of (1-hydroxy-2,3,4,9-tetrahydro-1*H*-pyrido[3,4-b]indol-1-yl)(phenyl)methanone (**8a**):

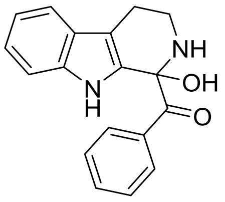

8a

CPS,MIYAPUR

Mass Analysis Report

**Data Filename**	090120035.d	**Sample Name**	A214/CARS-2/017 C
**Sample Type**	Sample	**Position**	Vial 88
**Instrument Name**	Instrument 1	**User Name**	
**Acq Method**	ESI.m	**IRM Calibration Status**	Success
**DA Method**	DA.m	**Comment**	

User Spectra

Fragmentor Voltage	Collision Energy	Ionization Mode
135	0	Esi

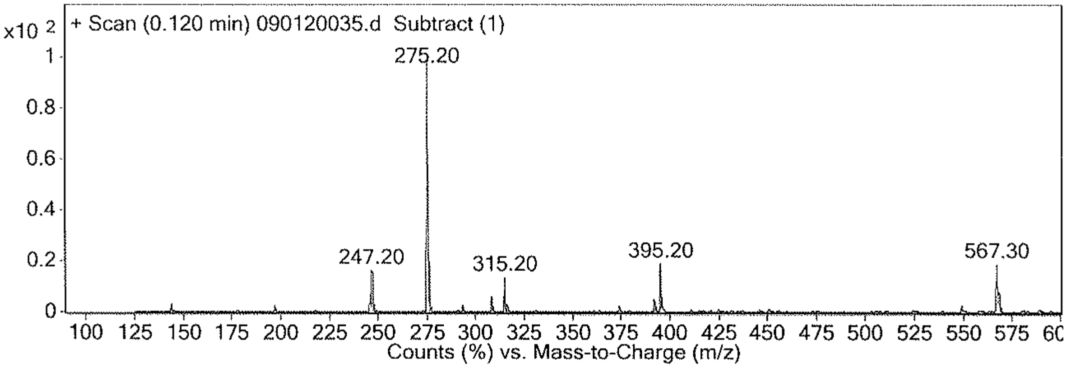

--- End Of Report ---

^{1}H NMR of (2,9-dihydro-1*H*-pyrido[3,4-b]indol-1-yl)(*p*-tolyl)methanone (**7b**):

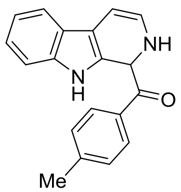

7b

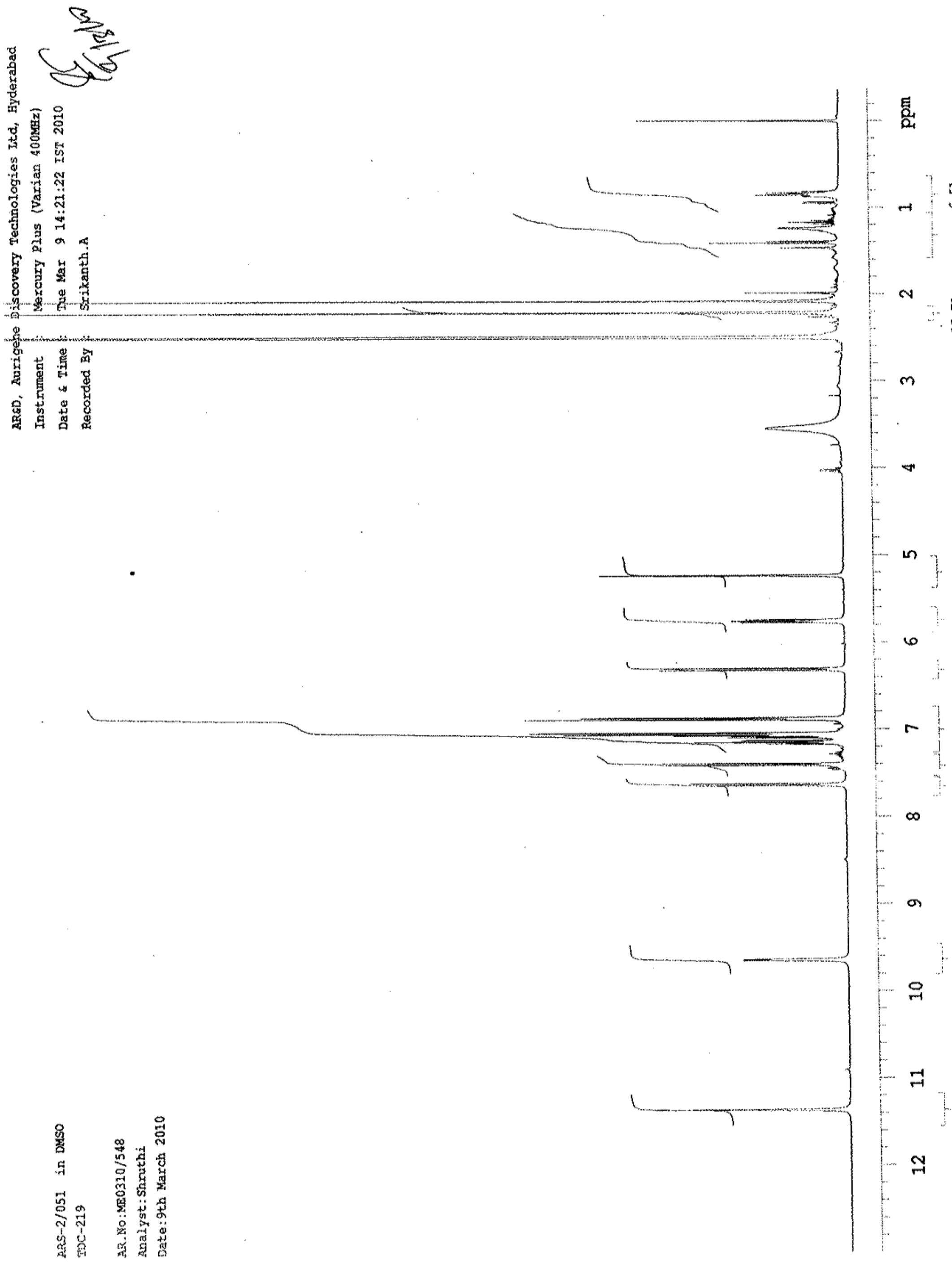

^{13}C NMR of (2,9-dihydro-1*H*-pyrido[3,4-b]indol-1-yl)(*p*-tolyl)methanone (**7b**):

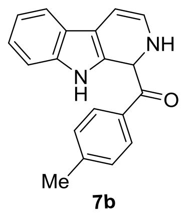

7b

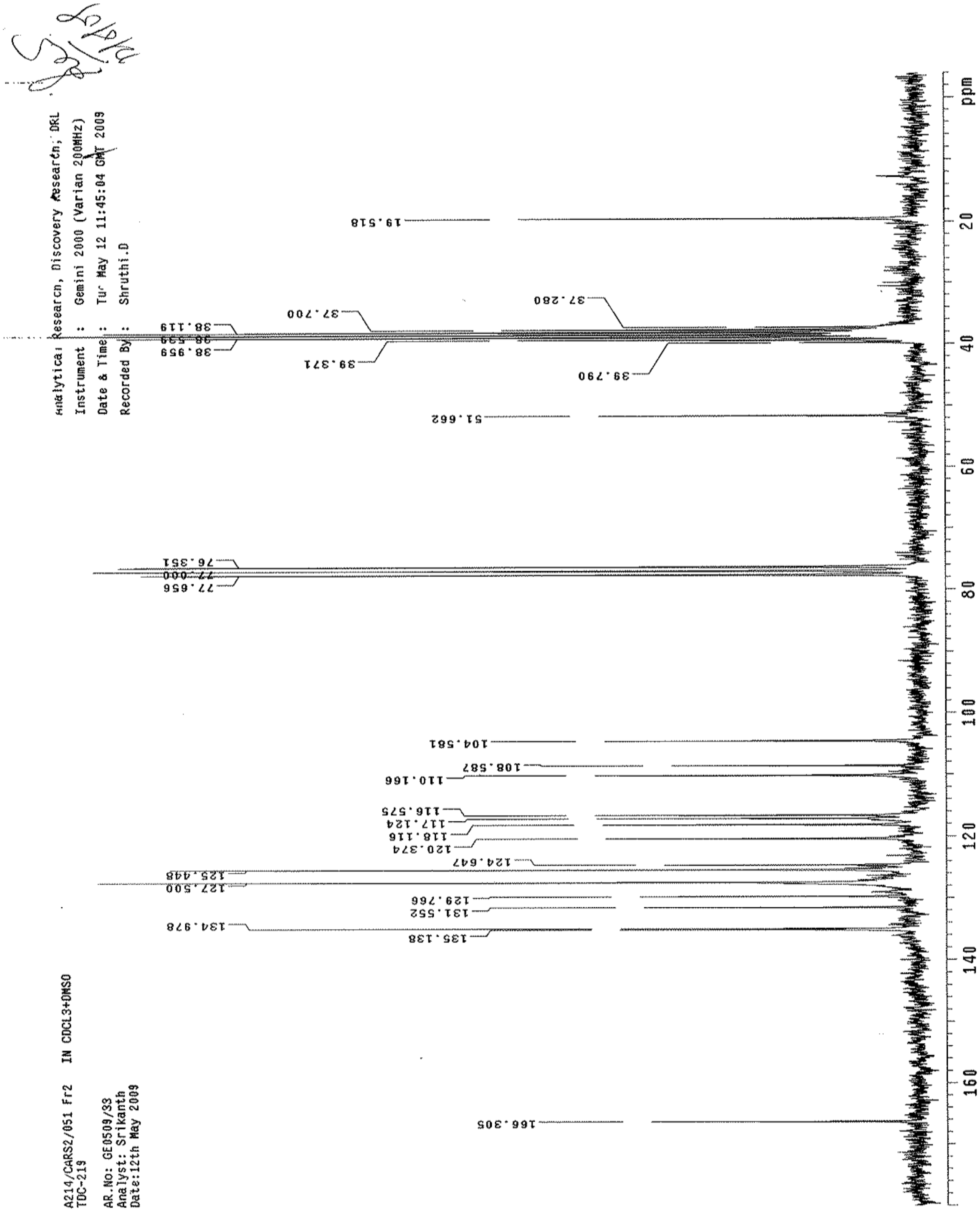

Mass spectrum of (2,9-dihydro-1*H*-pyrido[3,4-b]indol-1-yl)(*p*-tolyl)methanone (**7b**):

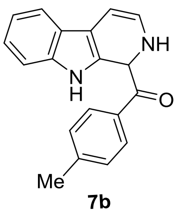

7b

CPS,MIYAPUR

Mass Analysis Report

**Data Filename**	090428029.d	**Sample Name**	A214/CARS-2/051 FR-2
**Sample Type**	Sample	**Position**	Vial 84
**Instrument Name**	Instrument 1	**User Name**	
**Acq Method**		**IRM Calibration Status**	Success
**DA Method**	Quant Process.m	**Comment**	

User Spectra

Fragmentor Voltage	Collision Energy	Ionization Mode
100	0	Esi

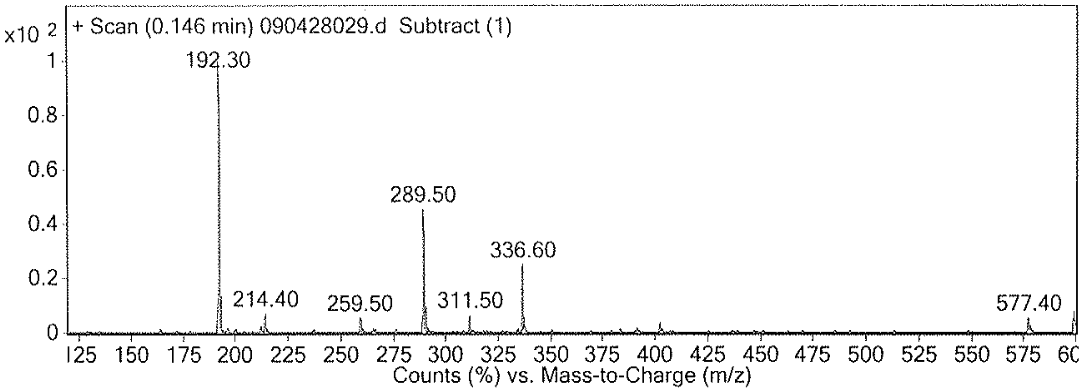

--- End Of Report ---

HRMS of (2,9-dihydro-1*H*-pyrido[3,4-b]indol-1-yl)(*p*-tolyl)methanone (**7b**):

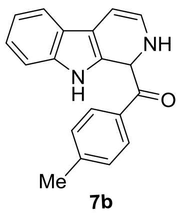

7b

Page 1

Elemental Composition Report

Single Mass Analysis

Tolerance = 5.0 PPM / DBE: min = -1.5, max = 80.0
Element prediction: Off
Number of isotope peaks used for i-FIT = 3

Monoisotopic Mass, Even Electron Ions
58 formula(e) evaluated with 1 results within limits (up to 4 best isotopic matches for each mass)
Elements Used:
C: 0-25 H: 0-25 N: 0-4 O: 0-4
9b
UT1212_150 8 (0.278) Cm (8:10)

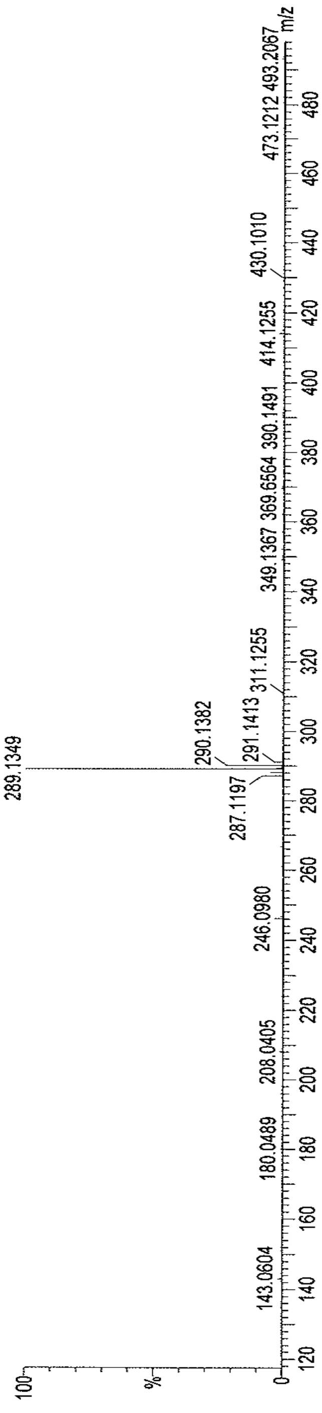

Minimum:				-1.5		
Maximum:		5.0	5.0	80.0		
Mass	Calc. Mass	mDa	PPM	DBE	i-FIT	Formula
289.1349	289.1341	0.8	2.8	12.5	5.4	C19 H17 N2 O

IR spectrum of (2,9-dihydro-1*H*-pyrido[3,4-b]indol-1-yl)(*p*-tolyl)methanone (**7b**):

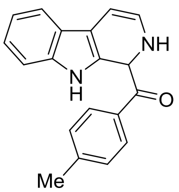

7b

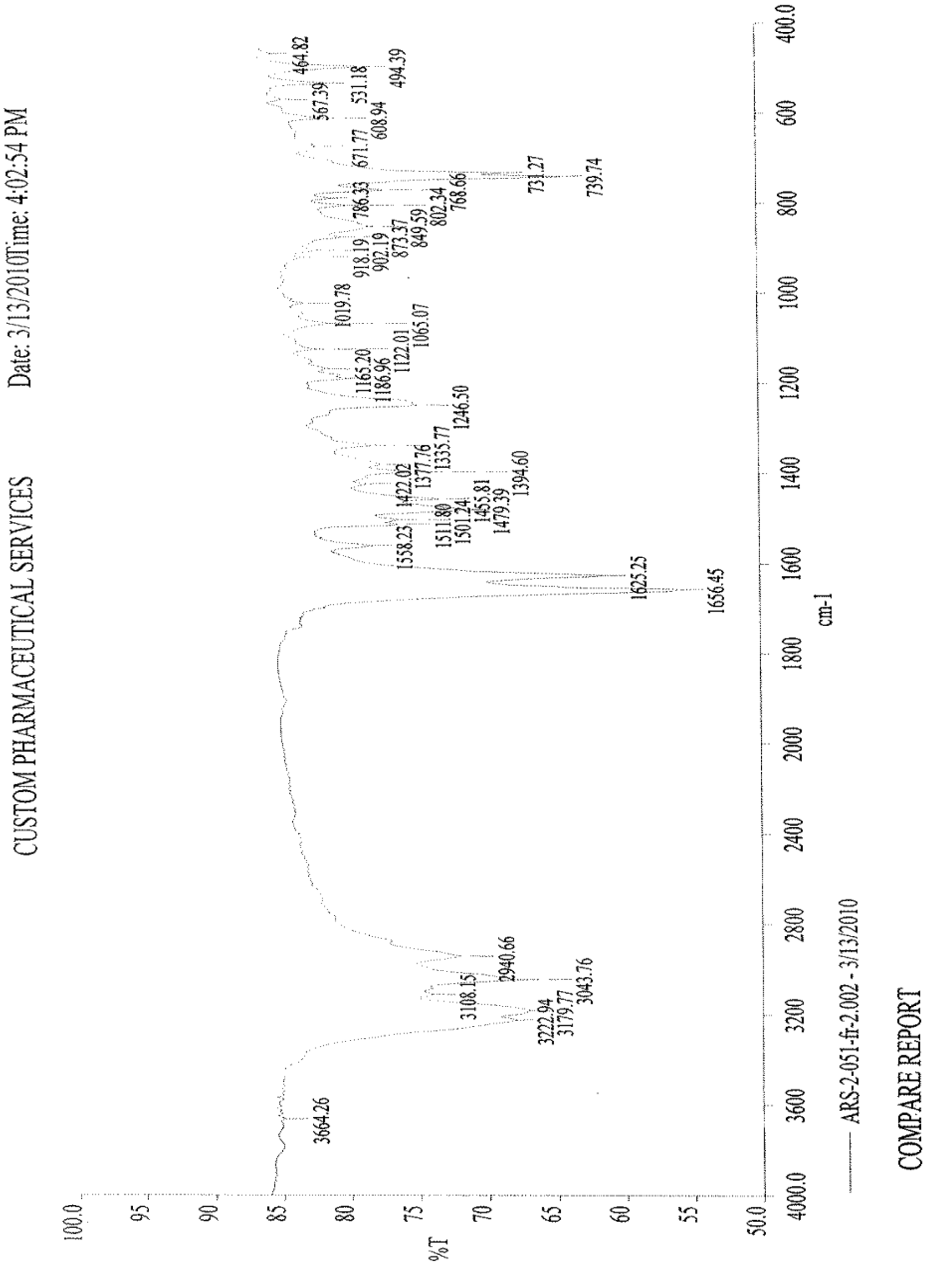

^{1}H NMR of (2,9-dihydro-1*H*-pyrido[3,4-b]indol-1-yl)(4-(trifluoromethyl)phenyl)methanone (**7c**):

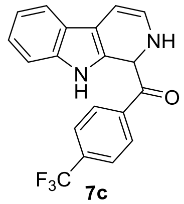

7c

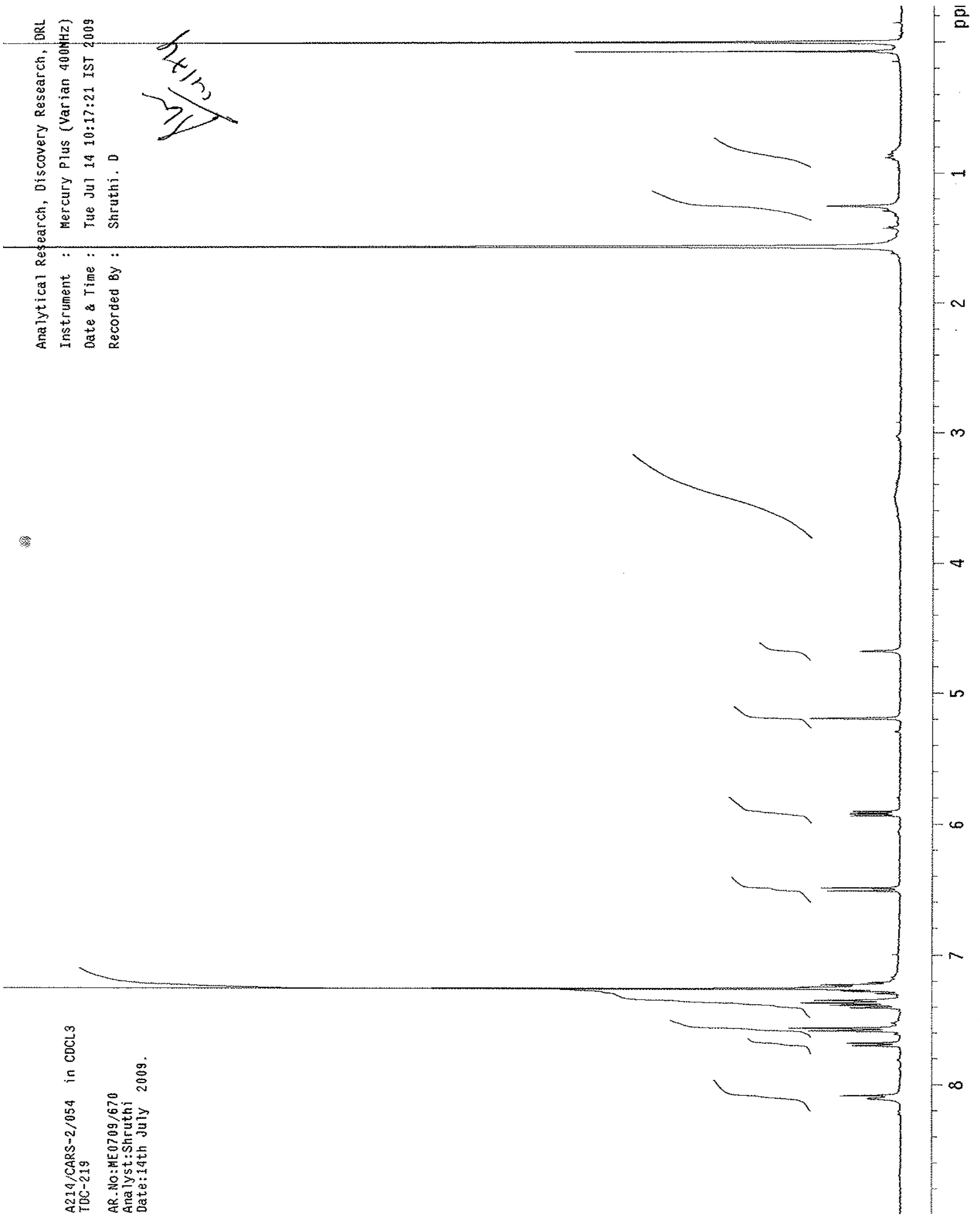

^{13}C NMR of (2,9-dihydro-1*H*-pyrido[3,4-b]indol-1-yl)(4-(trifluoromethyl)phenyl)methanone (**7c**):

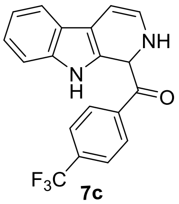

7c

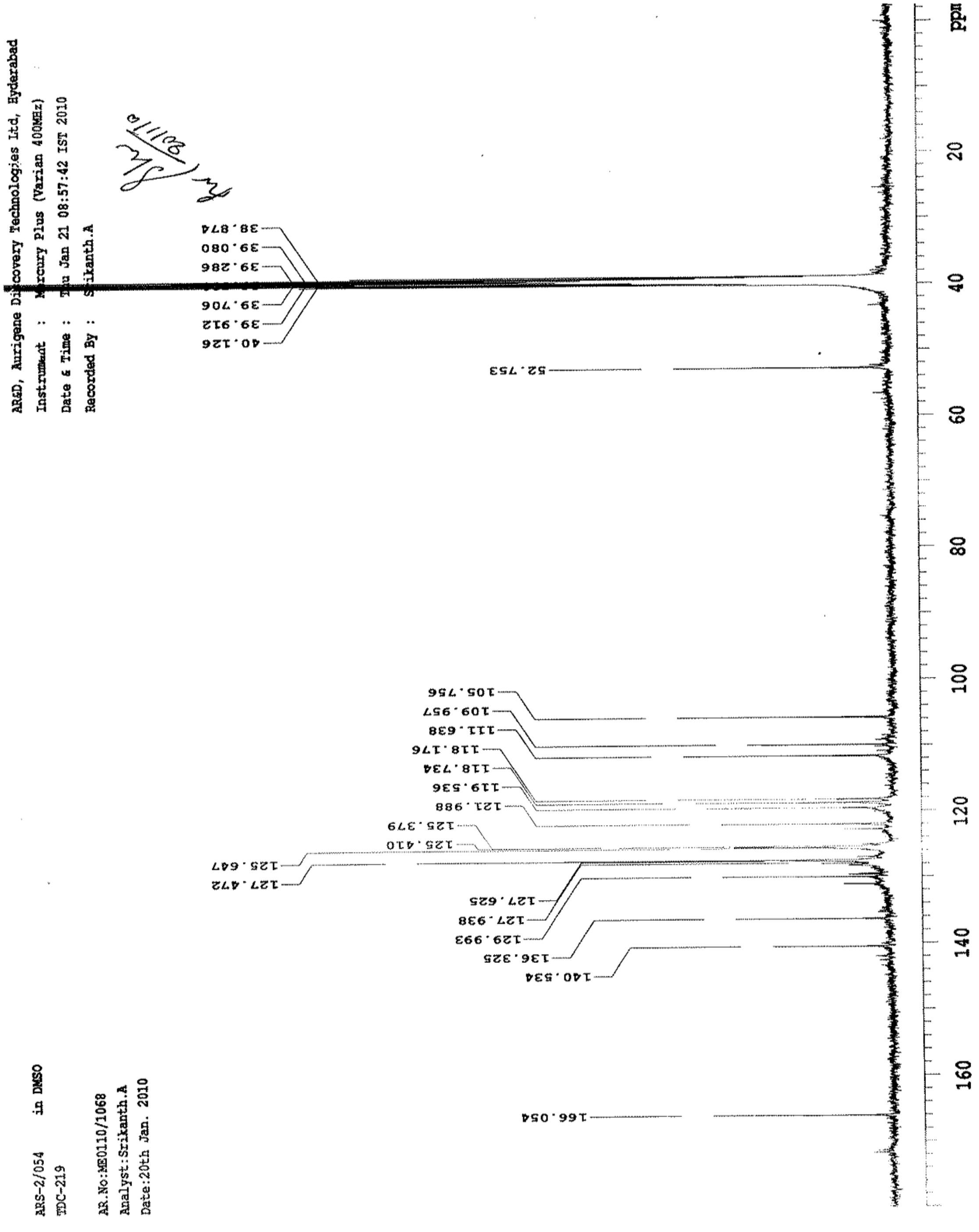

Mass spectrum of (2,9-dihydro-1*H*-pyrido[3,4-b]indol-1-yl)(4-(trifluoromethyl)phenyl)methanone (**7c**):

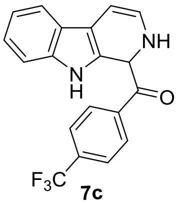

7c

CPS,MIYAPUR

Mass Analysis Report

**Data Filename**	090717015.d	**Sample Name**	CARS2/054
**Sample Type**	Sample	**Position**	Vial 14
**Instrument Name**	Instrument 1	**User Name**	
**Acq Method**		**IRM Calibration Status**	Success
**DA Method**	Quant Process.m	**Comment**	

User Spectra

Fragmentor Voltage	Collision Energy	Ionization Mode
100	0	Esi

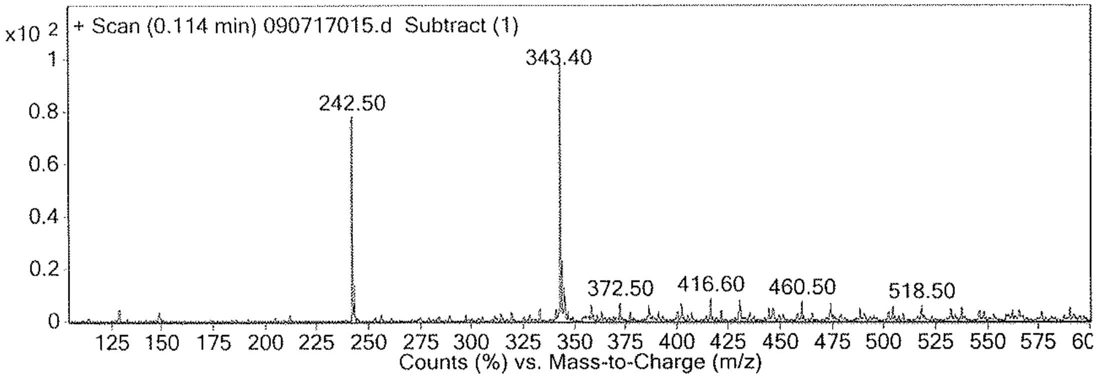

--- End Of Report ---

HRMS of (2,9-dihydro-1*H*-pyrido[3,4-b]indol-1-yl)(4-(trifluoromethyl)phenyl)methanone (**7c**):

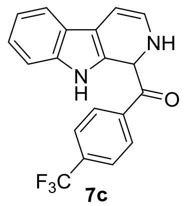

7c

Page 1

Elemental Composition Report

Single Mass Analysis

Tolerance = 6.0 PPM / DBE: min = -1.5, max = 80.0
Element prediction: Off
Number of isotope peaks used for i-FIT = 3

Monoisotopic Mass, Even Electron Ions
26 formula(e) evaluated with 1 results within limits (up to 4 best isotopic matches for each mass)
Elements Used:
C: 0-20 H: 0-20 N: 0-2 O: 0-2 F: 0-3
9c
UT1212_179 9 (0.337) Cm (9:10)

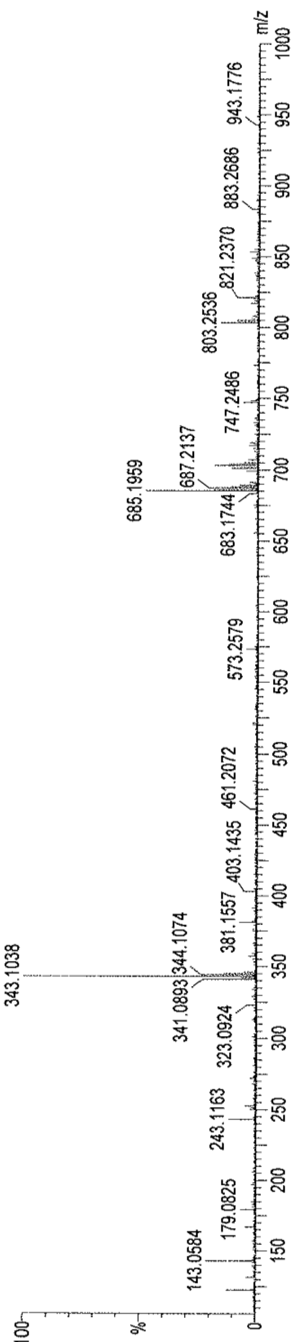

Minimum:				-1.5		
Maximum:		5.0	6.0	80.0		
Mass	Calc. Mass	mDa	PPM	DBE	i-FIT	Formula
343.1038	343.1058	-2.0	-5.8	12.5	11.6	C19 H14 N2 O F3

IR spectrum of (2,9-dihydro-1*H*-pyrido[3,4-b]indol-1-yl)(4-(trifluoromethyl)phenyl)methanone (**7c**):

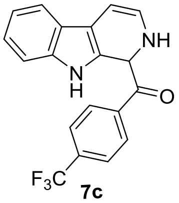

7c

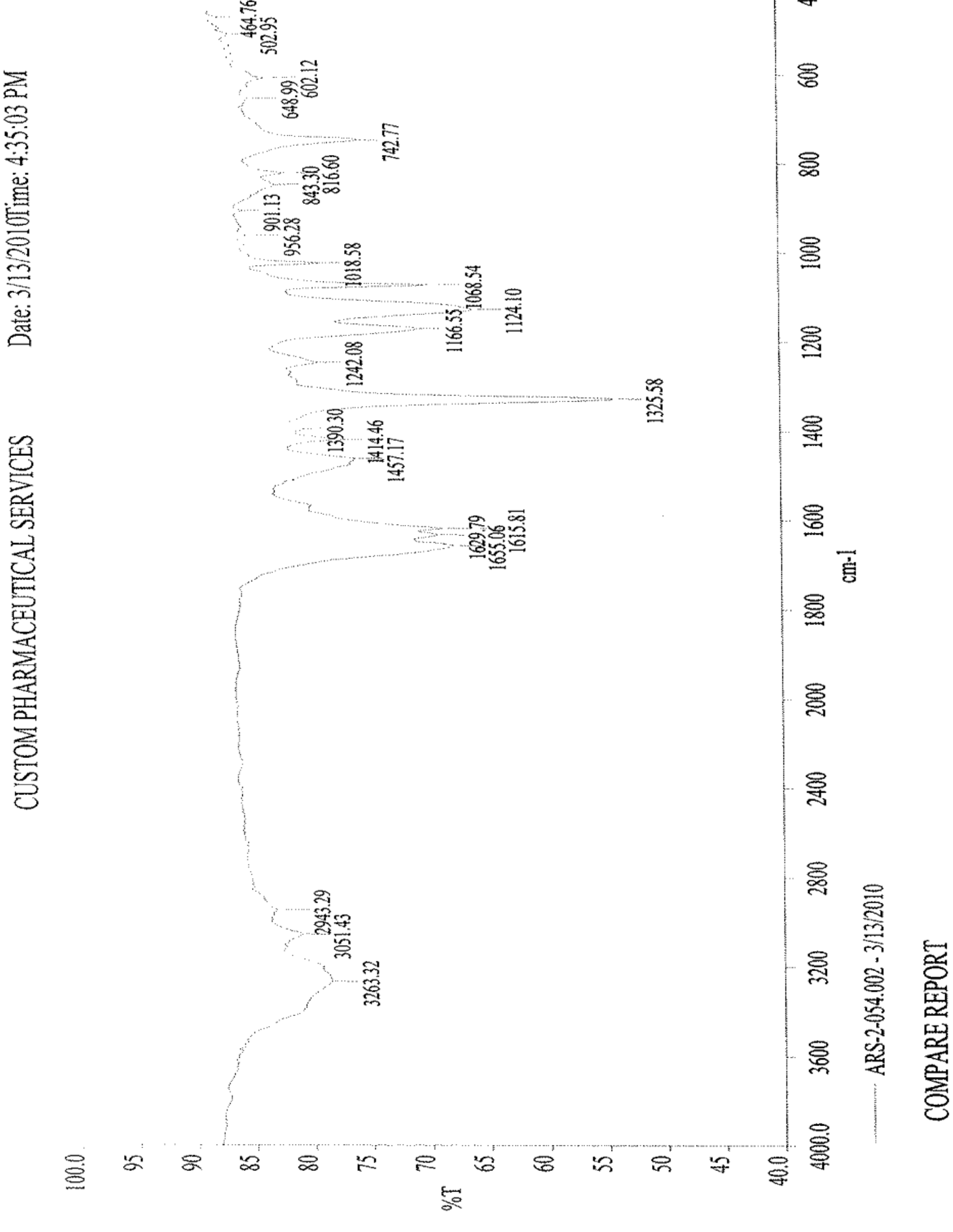

^{1}H NMR of (2,9-dihydro-1*H*-pyrido[3,4-b]indol-1-yl)(4-fluorophenyl)methanone (**7d**):

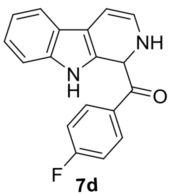

7d

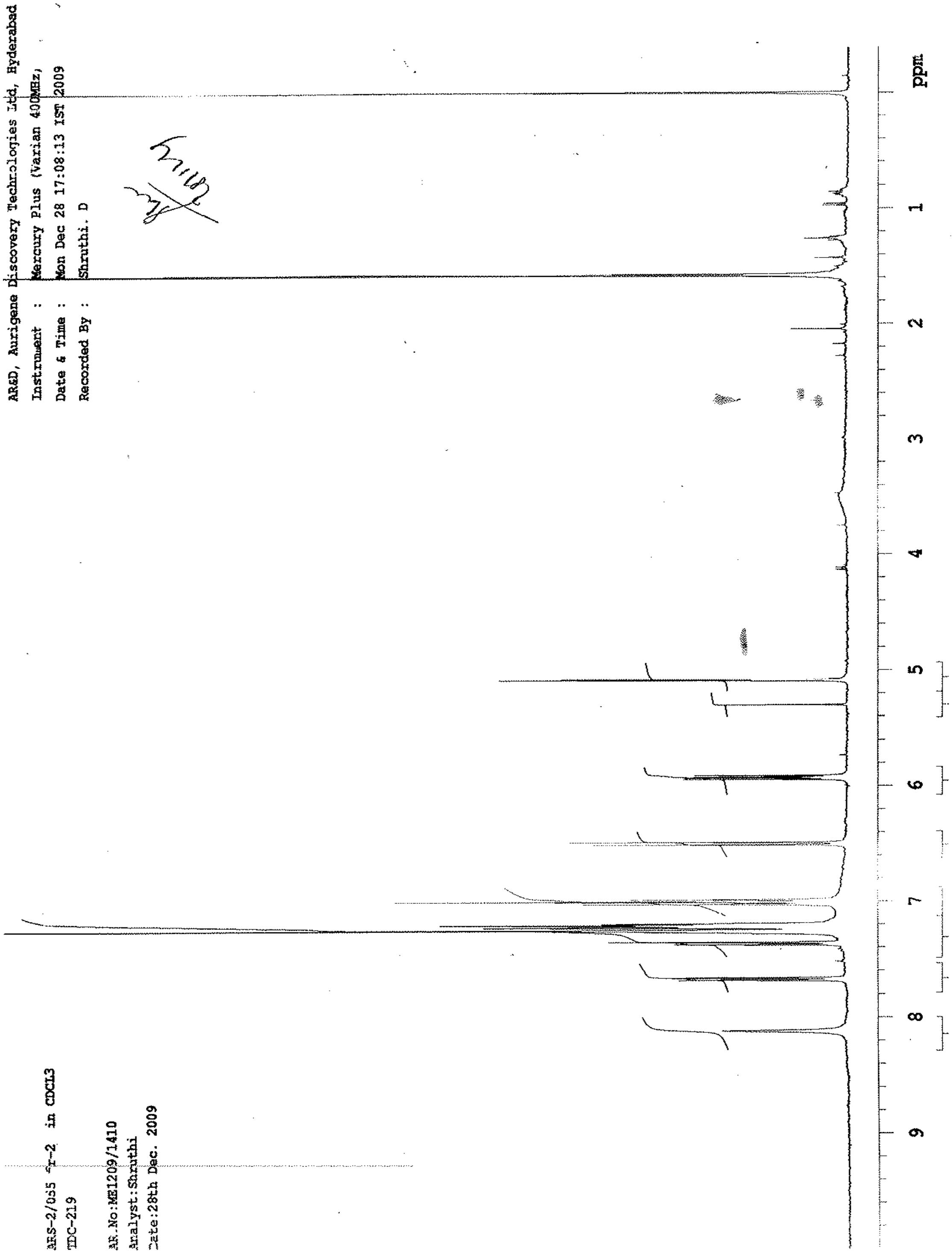

^{13}C NMR of (2,9-dihydro-1*H*-pyrido[3,4-b]indol-1-yl)(4-fluorophenyl)methanone (**7d**):

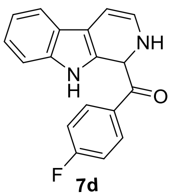

7d

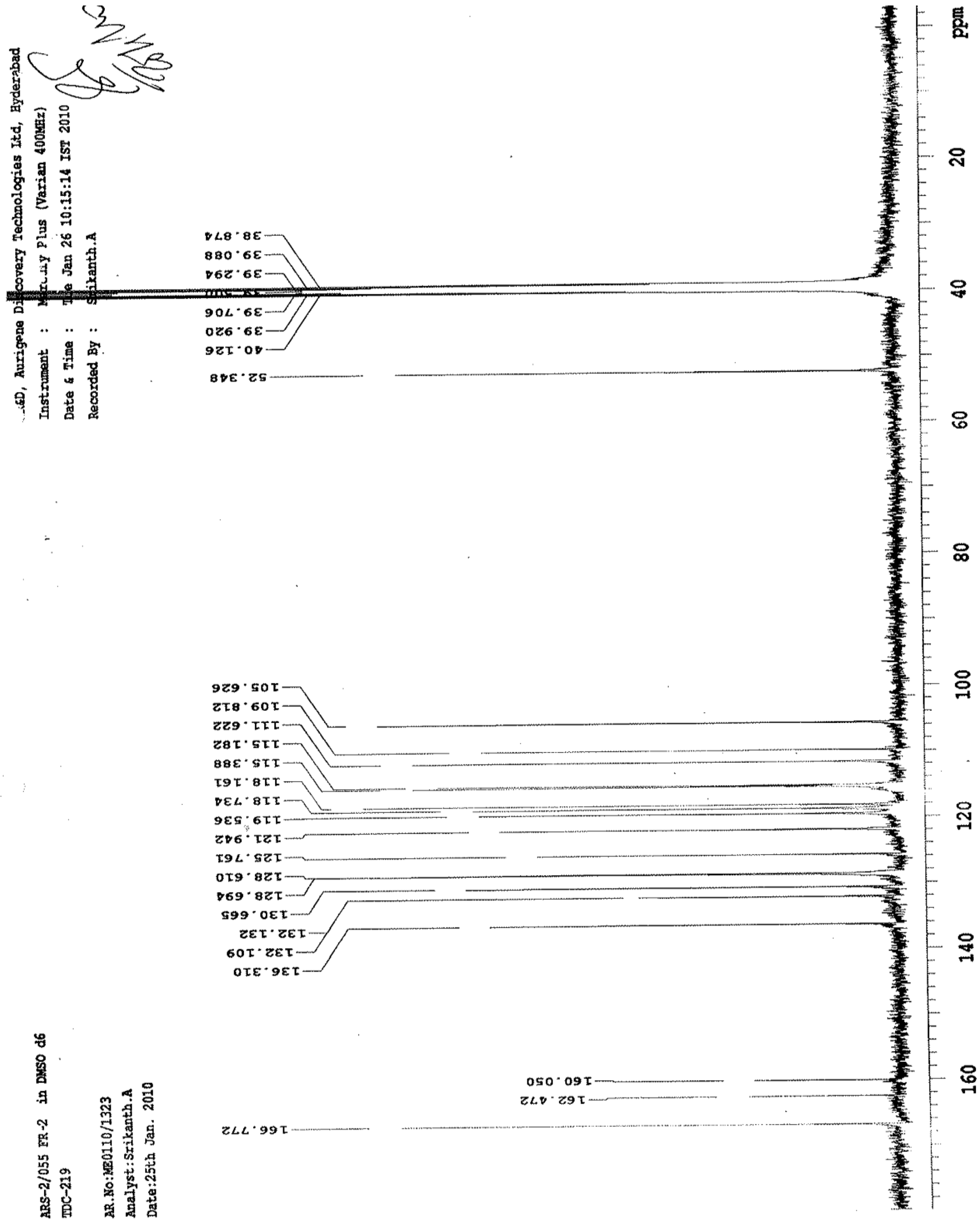

Mass spectrum of (2,9-dihydro-1*H*-pyrido[3,4-b]indol-1-yl)(4-fluorophenyl)methanone (**7d**):

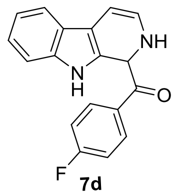

7d

CPS,MIYAPUR

Mass Analysis Report

**Data Filename**	091228020.d	**Sample Name**	ARS-2/055 FR-2
**Sample Type**	Sample	**Position**	Vial 5
**Instrument Name**	Instrument 1	**User Name**	
**Acq Method**	ESI.m	**IRM Calibration Status**	Success
**DA Method**	default.m	**Comment**	

User Spectra

Fragmentor Voltage	Collision Energy	Ionization Mode
125	0	Esi

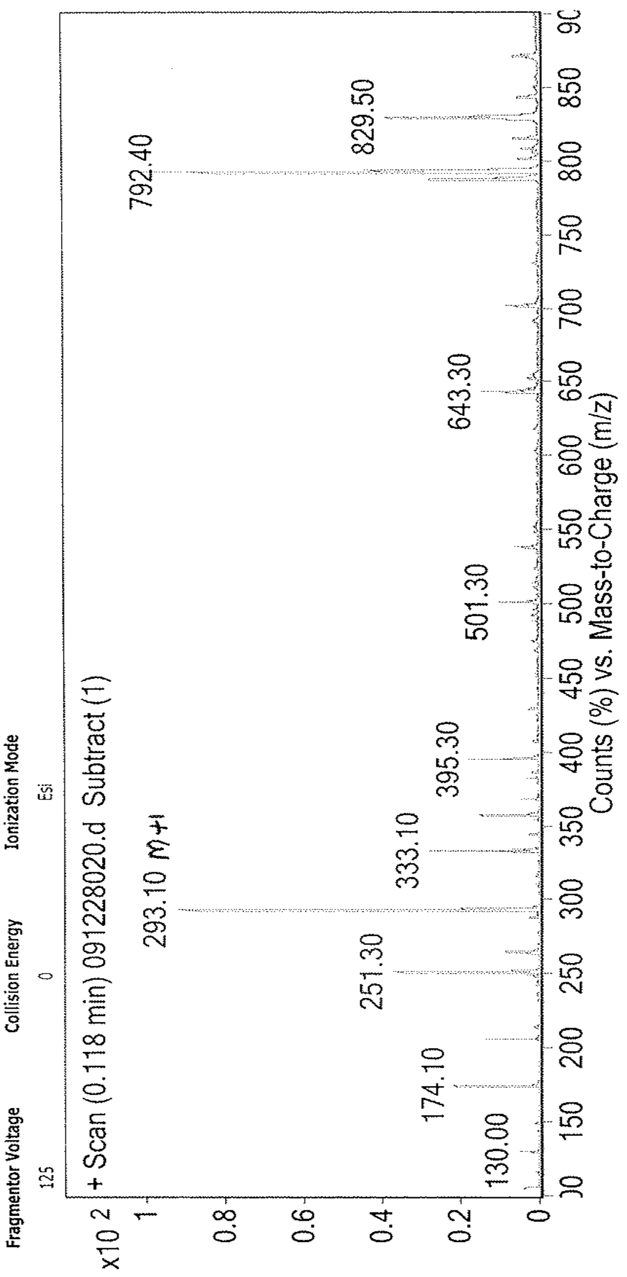

--- End Of Report ---

HRMS of (2,9-dihydro-1*H*-pyrido[3,4-b]indol-1-yl)(4-fluorophenyl)methanone (**7d**):

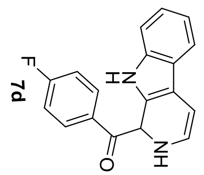

7d

Elemental Composition Report **Page 1**

Single Mass Analysis
Tolerance = 6.0 PPM / DBE: min = -1.5, max = 80.0
Element prediction: Off
Number of isotope peaks used for i-FIT = 3

Monoisotopic Mass, Even Electron Ions
62 formula(e) evaluated with 1 results within limits (up to 4 best isotopic matches for each mass)
Elements Used:
C: 0-20 H: 0-20 N: 0-6 O: 0-2 F: 0-1
9d
UT1212_181 7 (0.252) Cm (7:8)

Gajanan
1: TOF MS ES+
2.03e+003

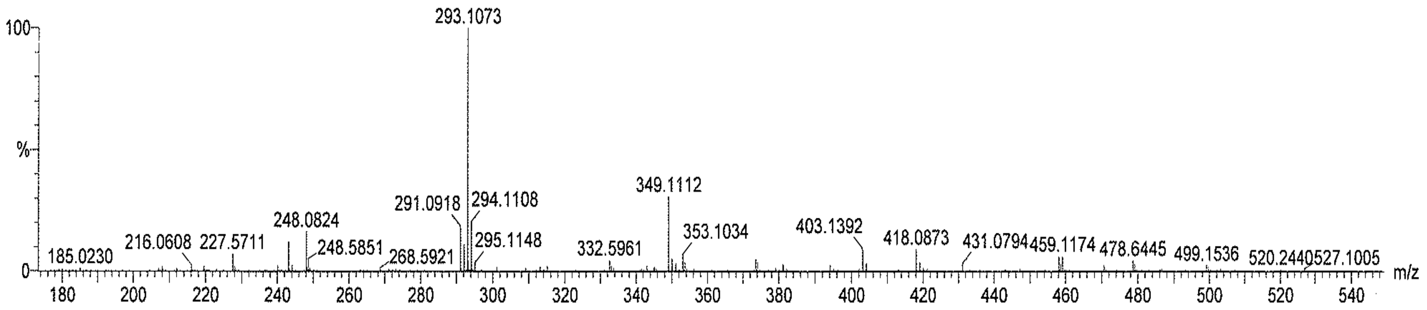

Minimum:				-1.5		
Maximum:		5.0	6.0	80.0		
Mass	Calc. Mass	mDa	PPM	DBE	i-FIT	Formula
293.1073	293.1090	-1.7	-5.8	12.5	3.9	C18 H14 N2 O F

IR spectrum of (2,9-dihydro-1*H*-pyrido[3,4-b]indol-1-yl)(4-fluorophenyl)methanone (**7d**):

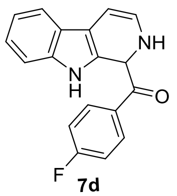

7d

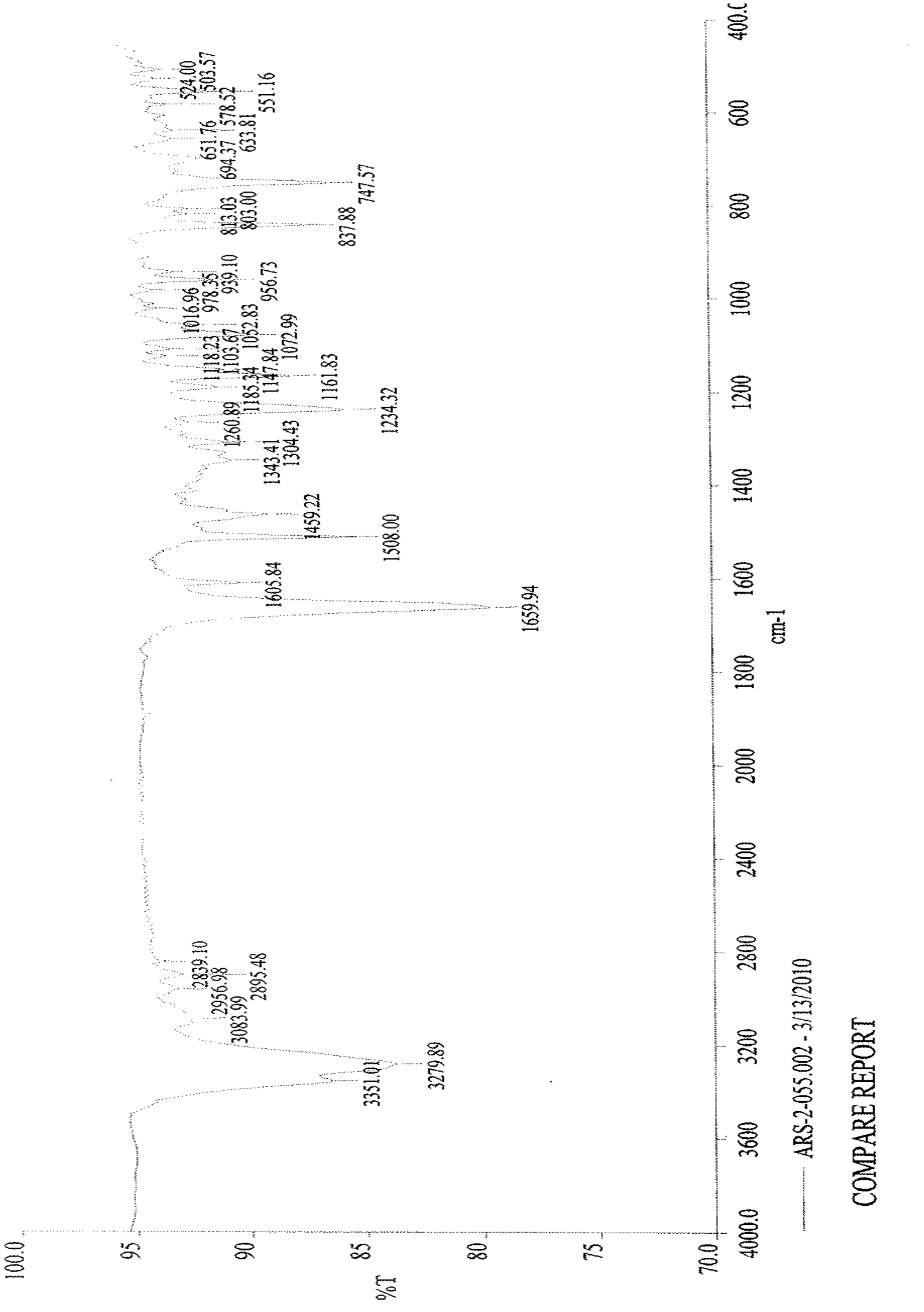

^{1}H NMR of (4-fluorophenyl)(1-hydroxy-2,3,4,9-tetrahydro-1*H*-pyrido[3,4-b]indol-1-yl)methanone (**8d**):

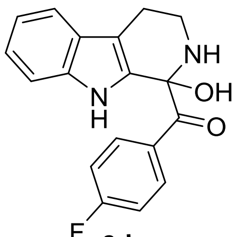

8d

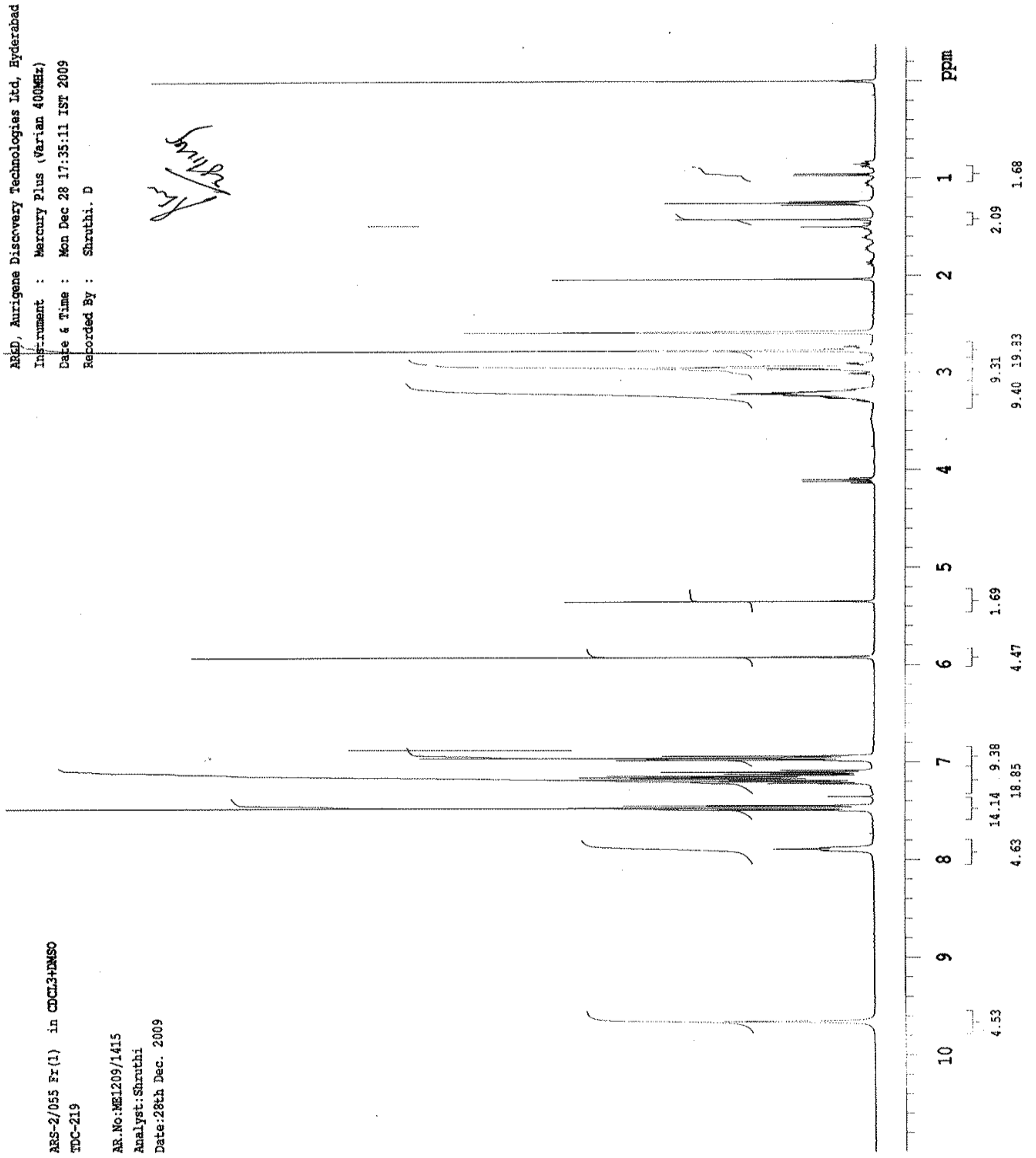

^{13}C NMR of (4-fluorophenyl)(1-hydroxy-2,3,4,9-tetrahydro-1*H*-pyrido[3,4-b]indol-1-yl)methanone (**8d**):

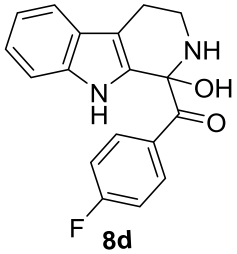

8d

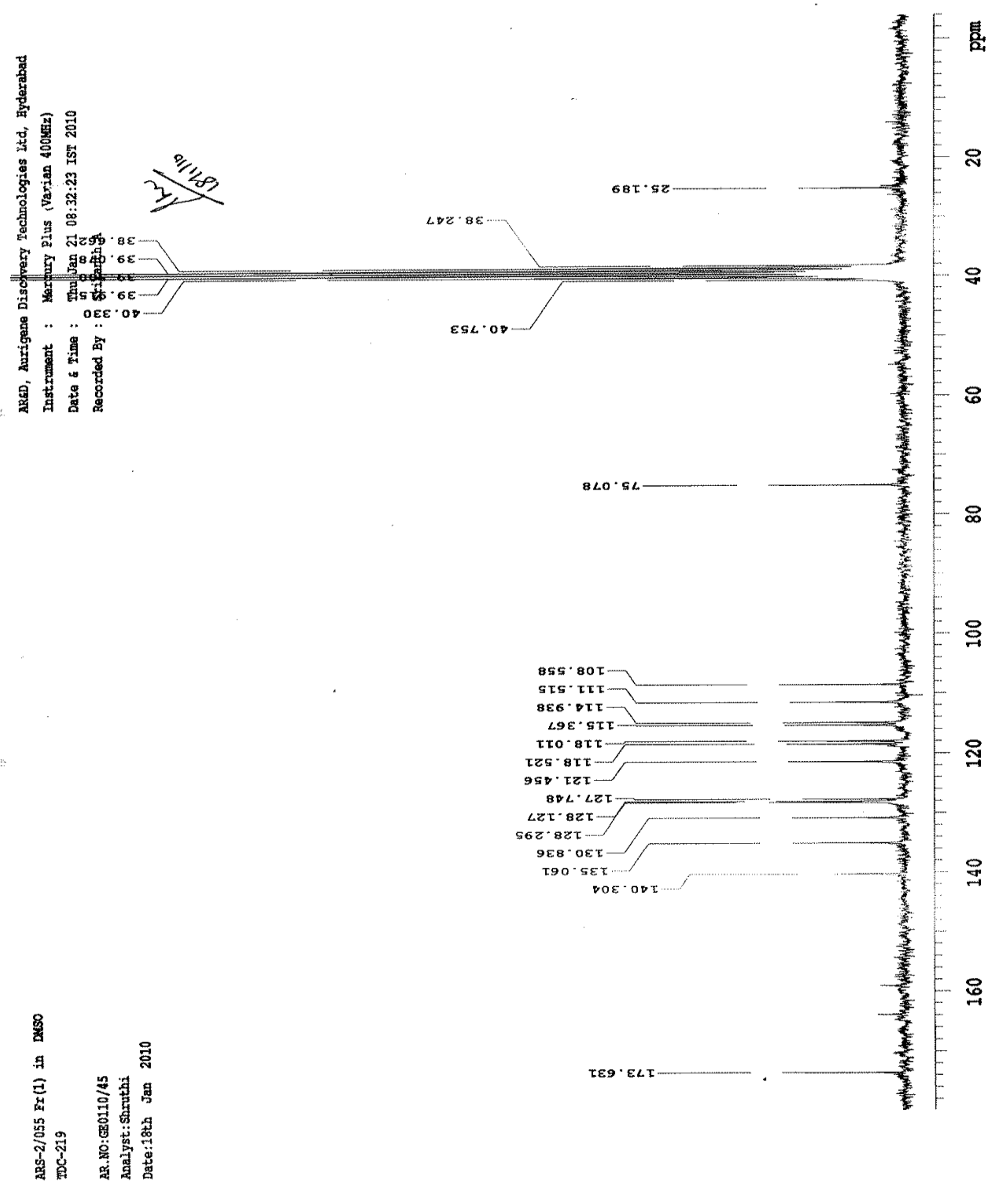

Mass spectrum of (4-fluorophenyl)(1-hydroxy-2,3,4,9-tetrahydro-1*H*-pyrido[3,4-b]indol-1-yl)methanone (**8d**):

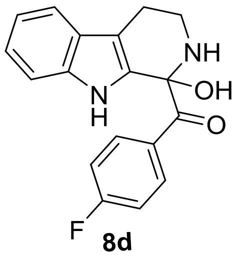

8d

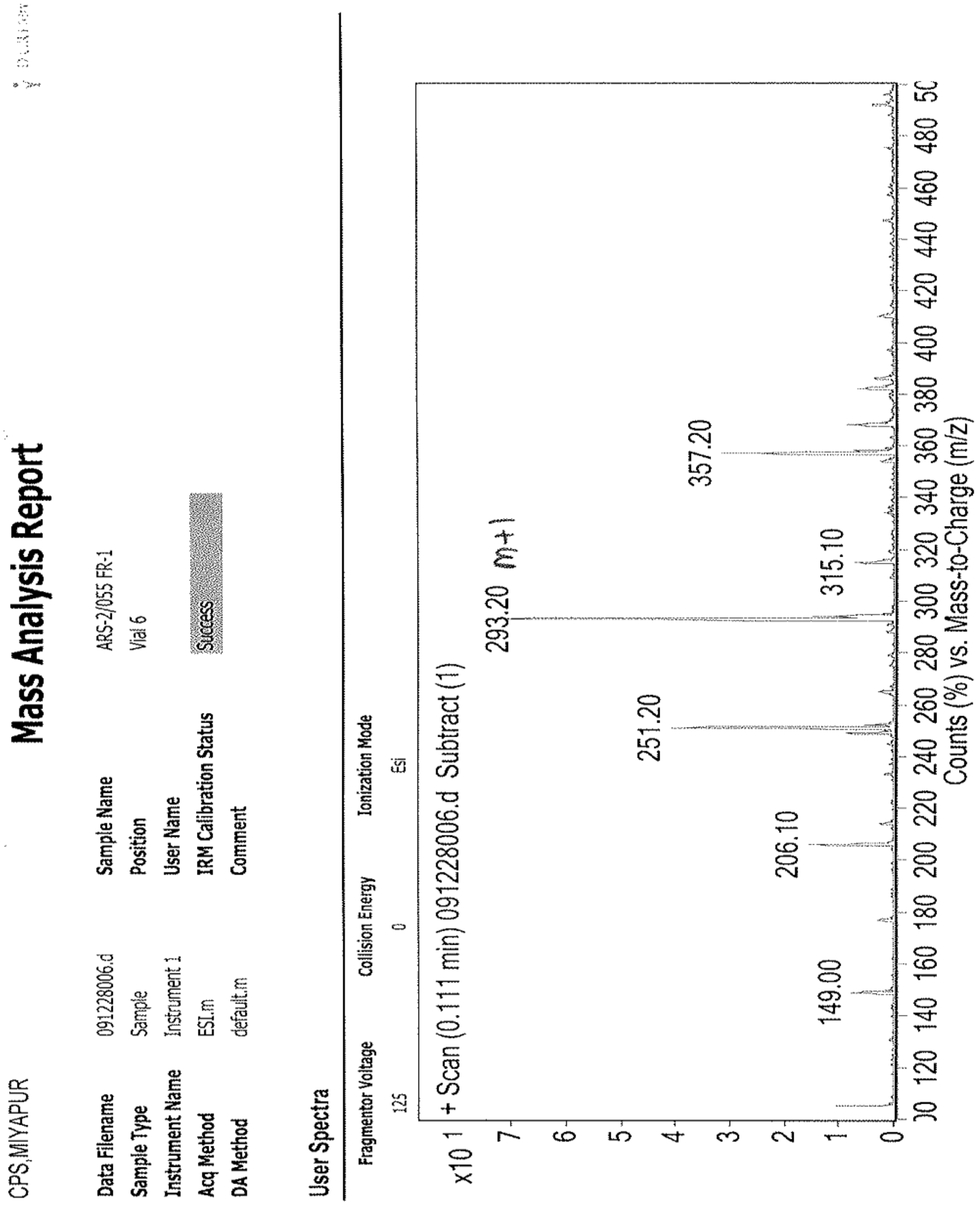

HRMS of (4-fluorophenyl)(1-hydroxy-2,3,4,9-tetrahydro-1*H*-pyrido[3,4-b]indol-1-yl)methanone (**8d**):

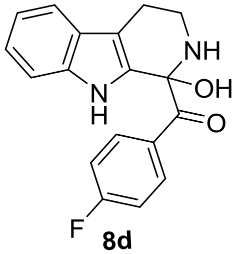

8d

Page 1

Elemental Composition Report

Single Mass Analysis

Tolerance = 7.0 PPM / DBE: min = -1.5, max = 80.0
Element prediction: Off
Number of isotope peaks used for i-FIT = 3

Monoisotopic Mass, Even Electron Ions
40 formula(e) evaluated with 2 results within limits (up to 4 best isotopic matches for each mass)
Elements Used:
C: 0-25 H: 0-25 N: 0-2 O: 0-2 F: 0-1
8d
UT1212_175 5 (0.198) Cm (5)

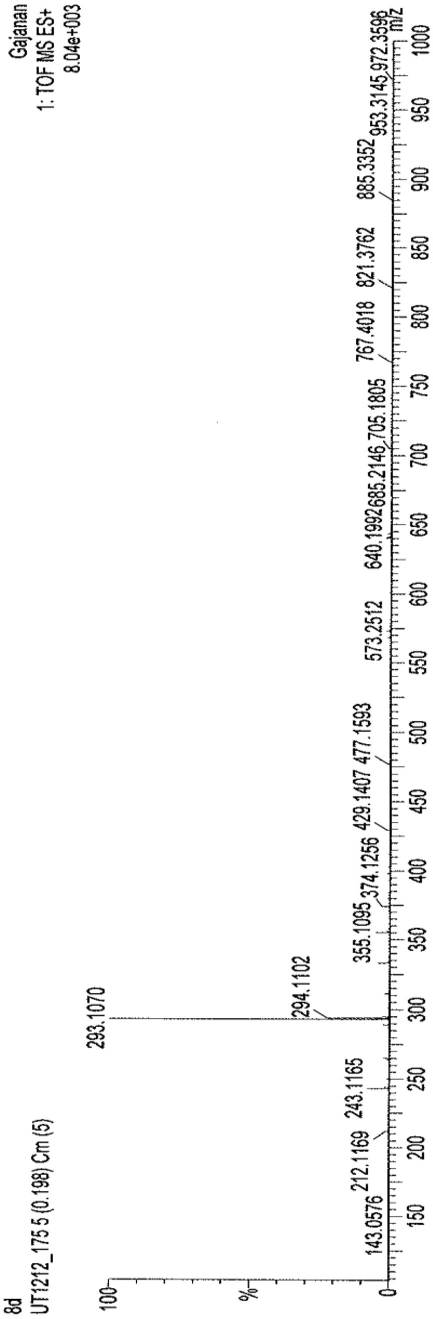

Minimum:				-1.5		
Maximum:		5.0	7.0	80.0		

Mass	Calc. Mass	mDa	PPM	DBE	i-FIT	Formula
293.1070	293.1079	-0.9	-3.1	16.5	6.4	C21 H13 N2
	293.1090	-2.0	-6.8	12.5	7.0	C18 H14 N2 O F

IR spectrum (4-fluorophenyl)(1-hydroxy-2,3,4,9-tetrahydro-1*H*-pyrido[3,4-b]indol-1-yl)methanone (**8d**):

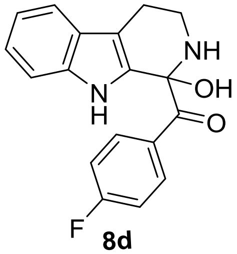

8d

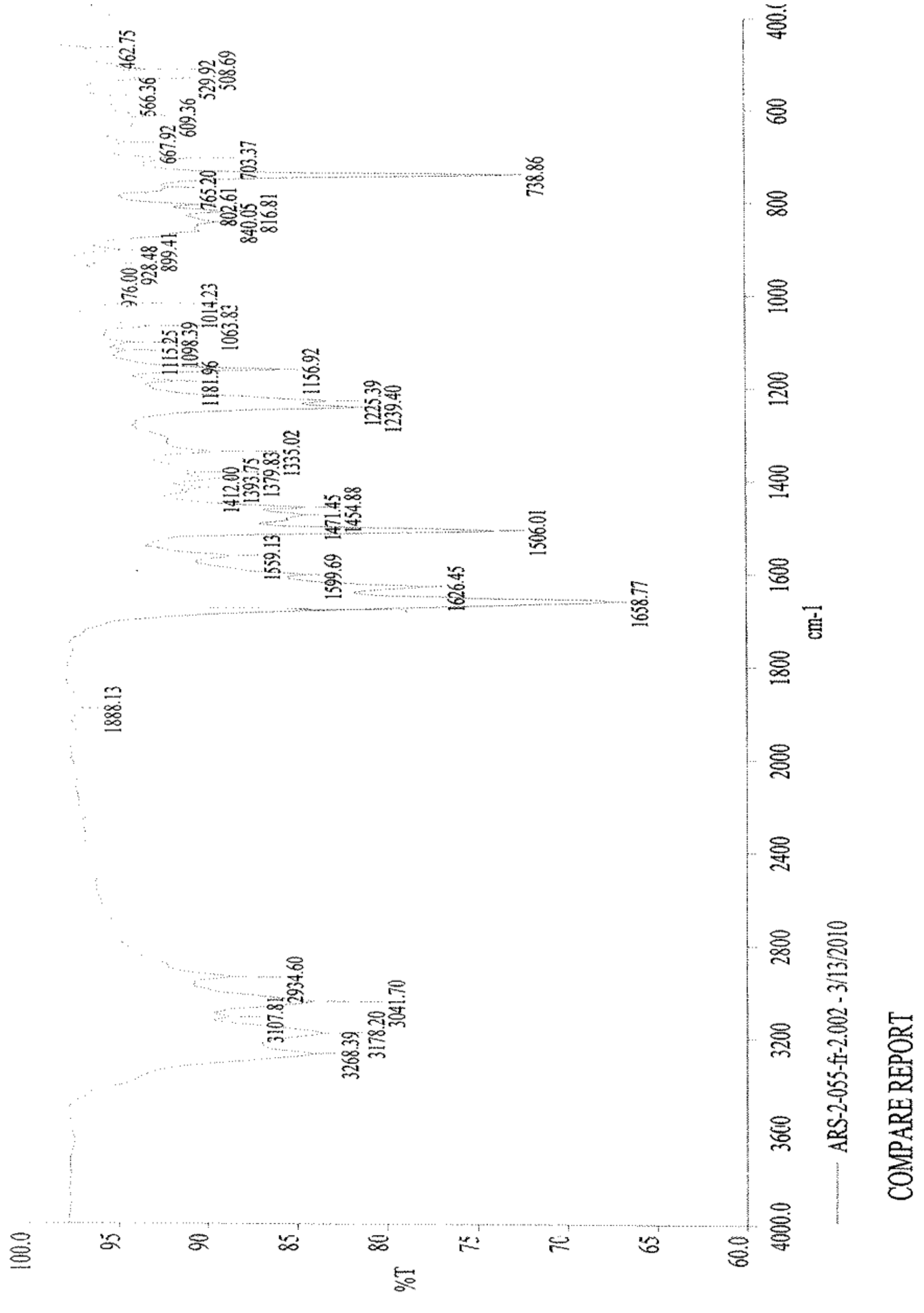

^{1}H NMR of (2,9-dihydro-1*H*-pyrido[3,4-b]indol-1-yl)(3-nitrophenyl)methanone (**7e**):

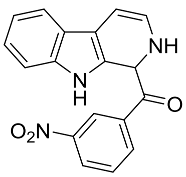

7e

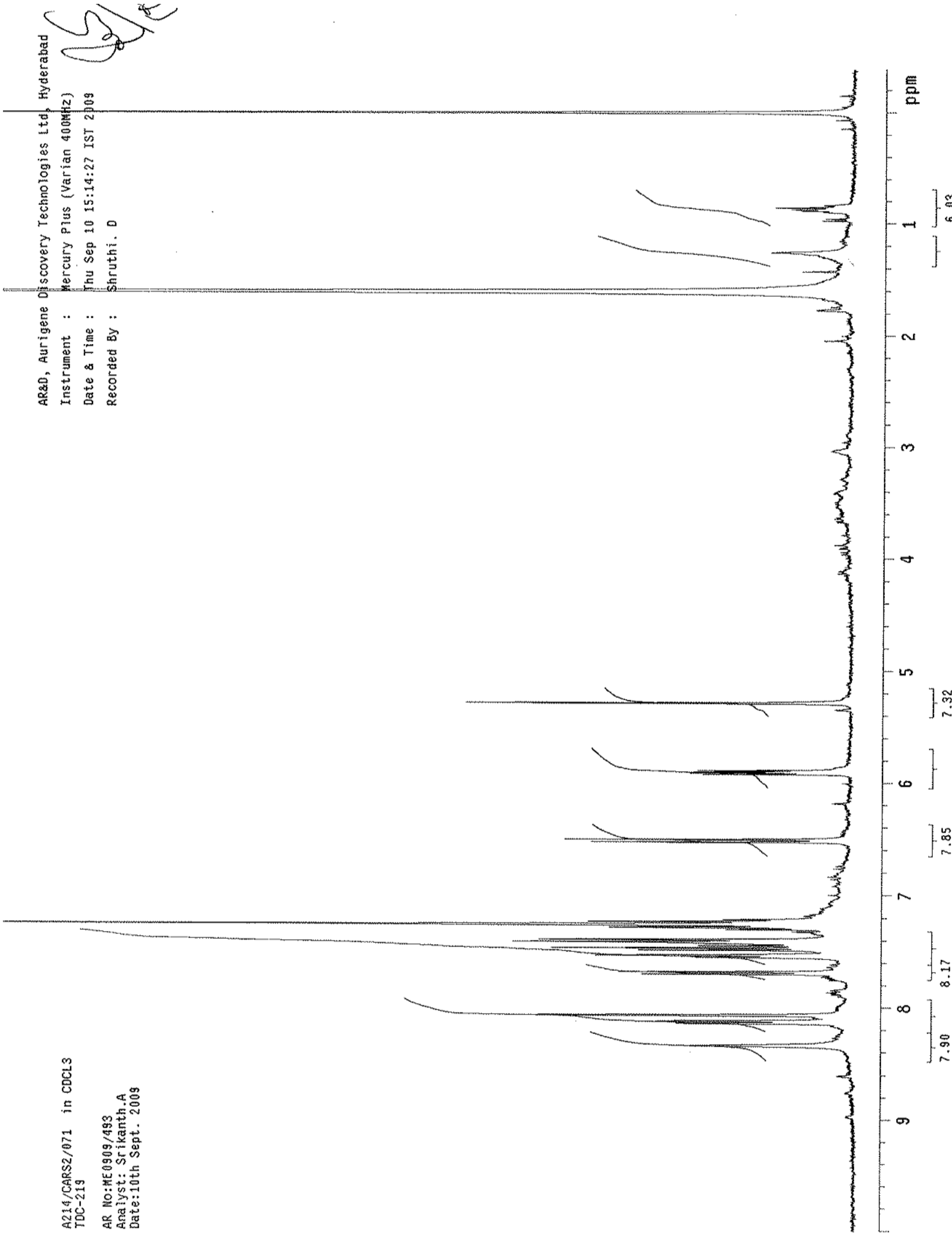

Mass spectrum of (2,9-dihydro-1*H*-pyrido[3,4-b]indol-1-yl)(3-nitrophenyl)methanone (**7e**):

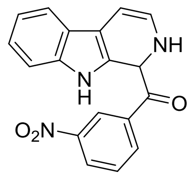

7e

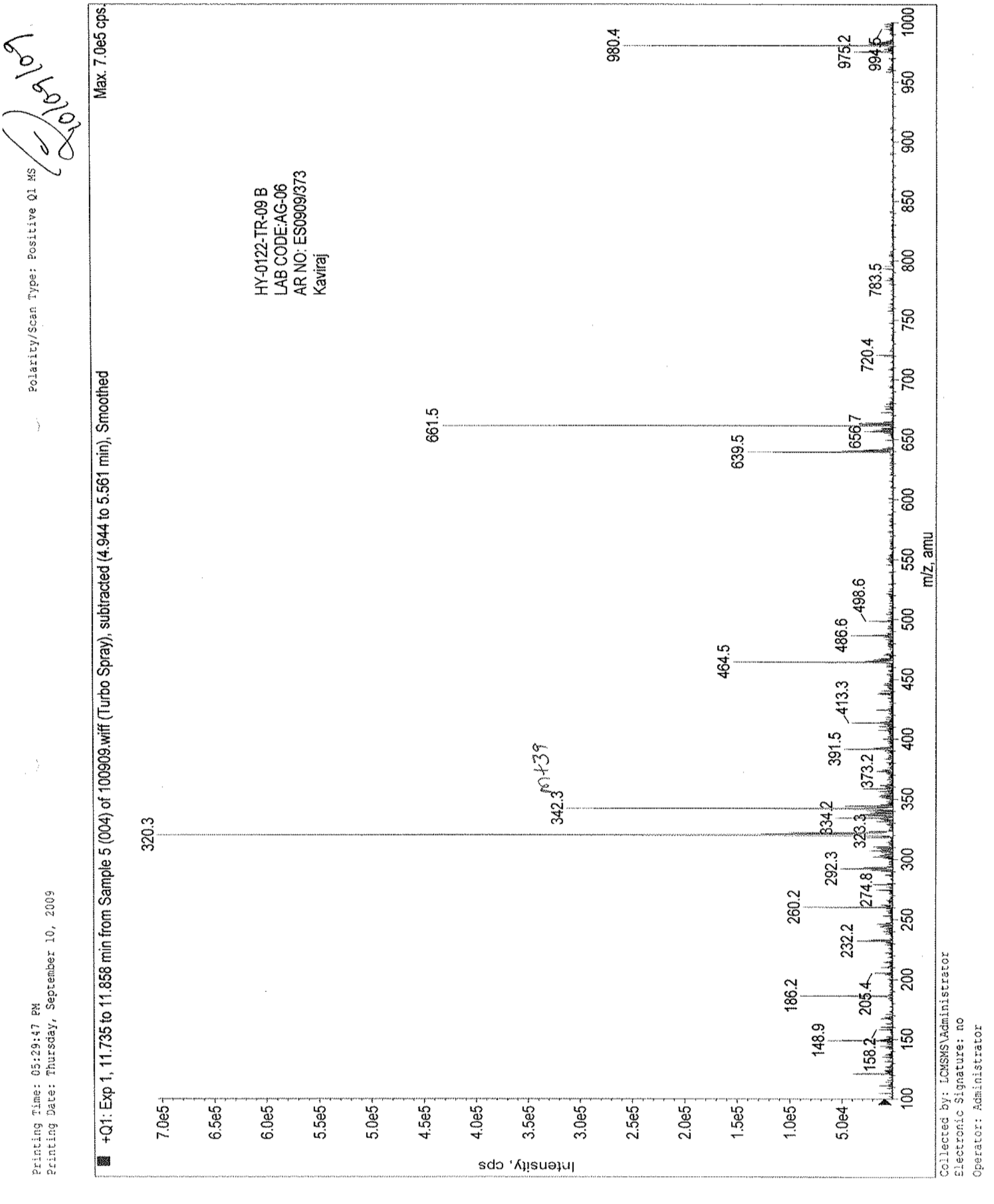

^{1}H NMR of (2,9-dihydro-1*H*-pyrido[3,4-b]indol-1-yl)(4-methoxyphenyl)methanone (**7f**):

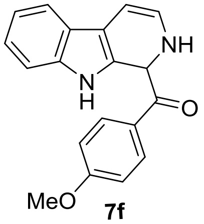

7f

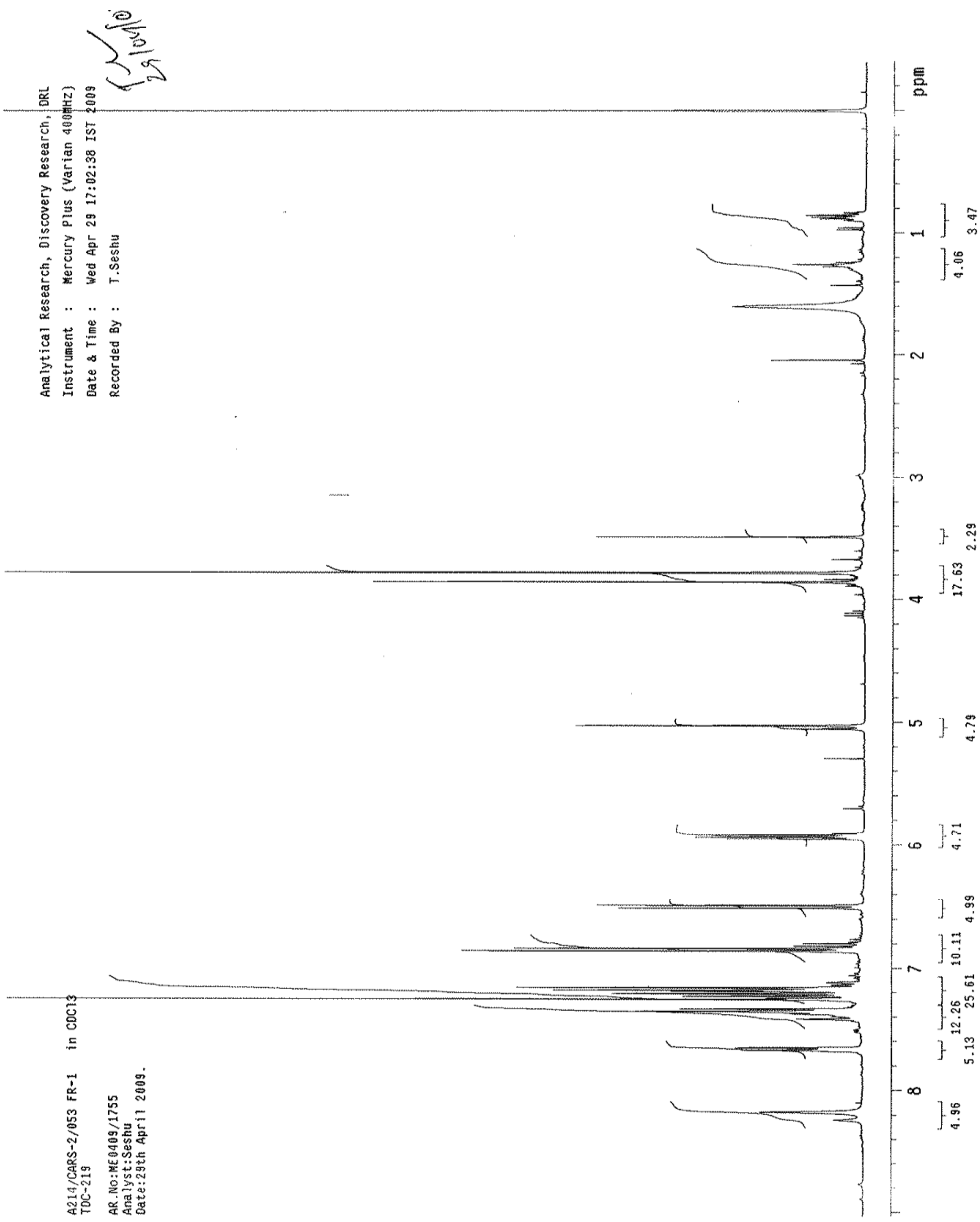

^{13}C NMR of (2,9-dihydro-1*H*-pyrido[3,4-b]indol-1-yl)(4-methoxyphenyl)methanone (**7f**):

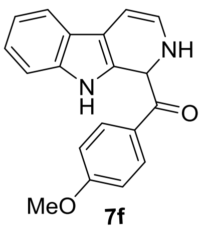

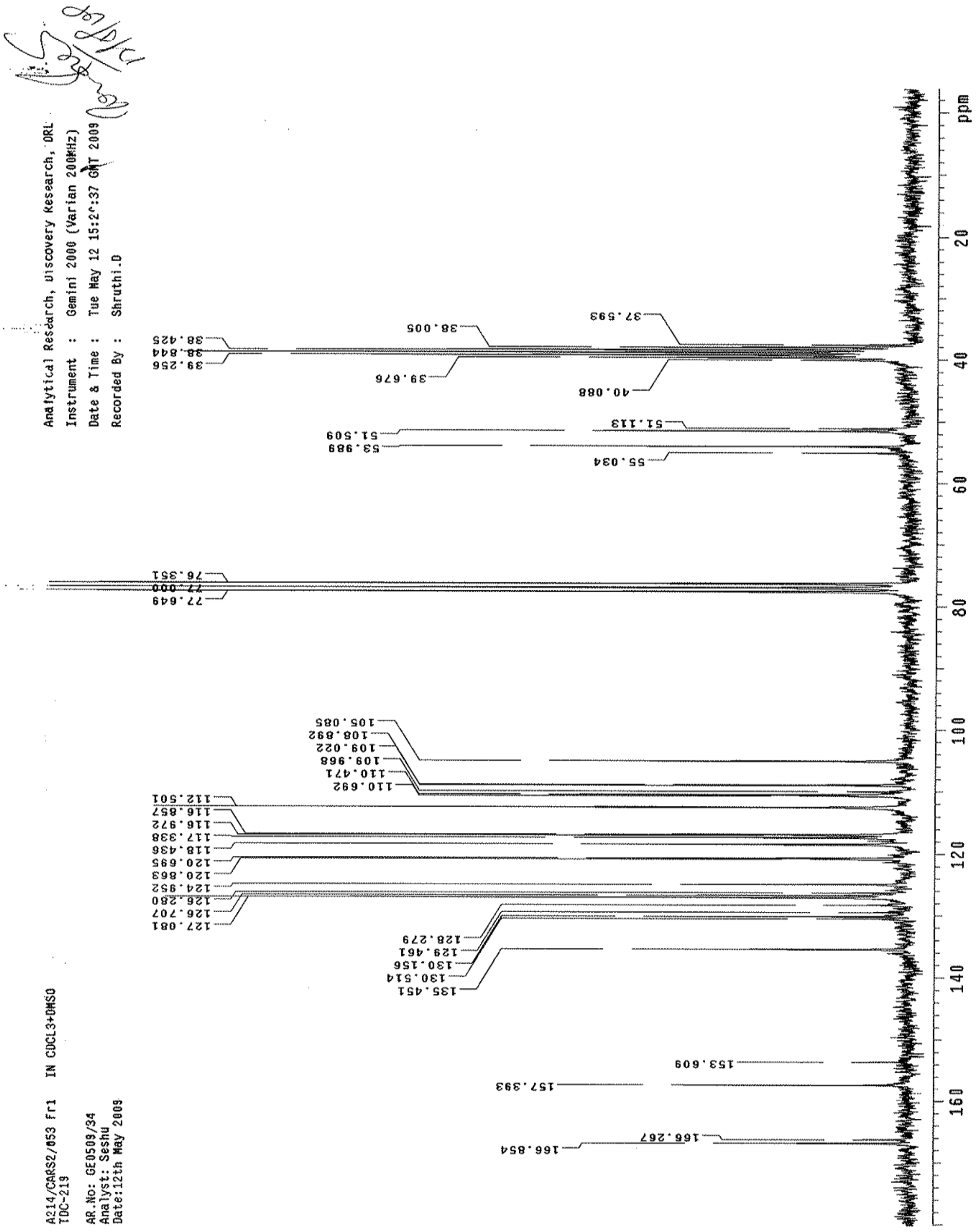

Mass spectrum of (2,9-dihydro-1*H*-pyrido[3,4-b]indol-1-yl)(4-methoxyphenyl)methanone (**7f**):

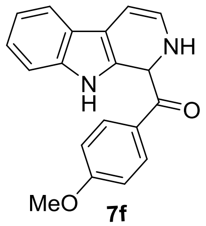

CPS,MIYAPUR

Mass Analysis Report

**Data Filename**	090429023.d	**Sample Name**	A214/CARS-2/053 FR-1
**Sample Type**	Sample	**Position**	Vial 42
**Instrument Name**	Instrument 1	**User Name**	
**Acq Method**		**IRM Calibration Status**	Success
**DA Method**	Quant Process.m	**Comment**	

User Spectra

Fragmentor Voltage	Collision Energy	Ionization Mode
135	0	Esi

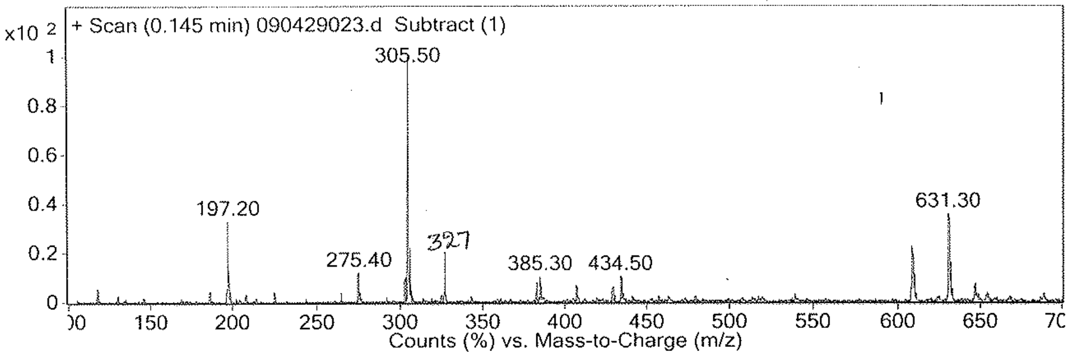

--- End Of Report ---

IR spectrum of (2,9-dihydro-1*H*-pyrido[3,4-b]indol-1-yl)(4-methoxyphenyl)methanone (**7f**):

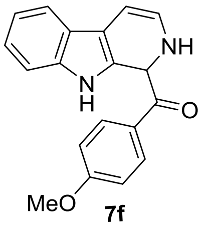

7f

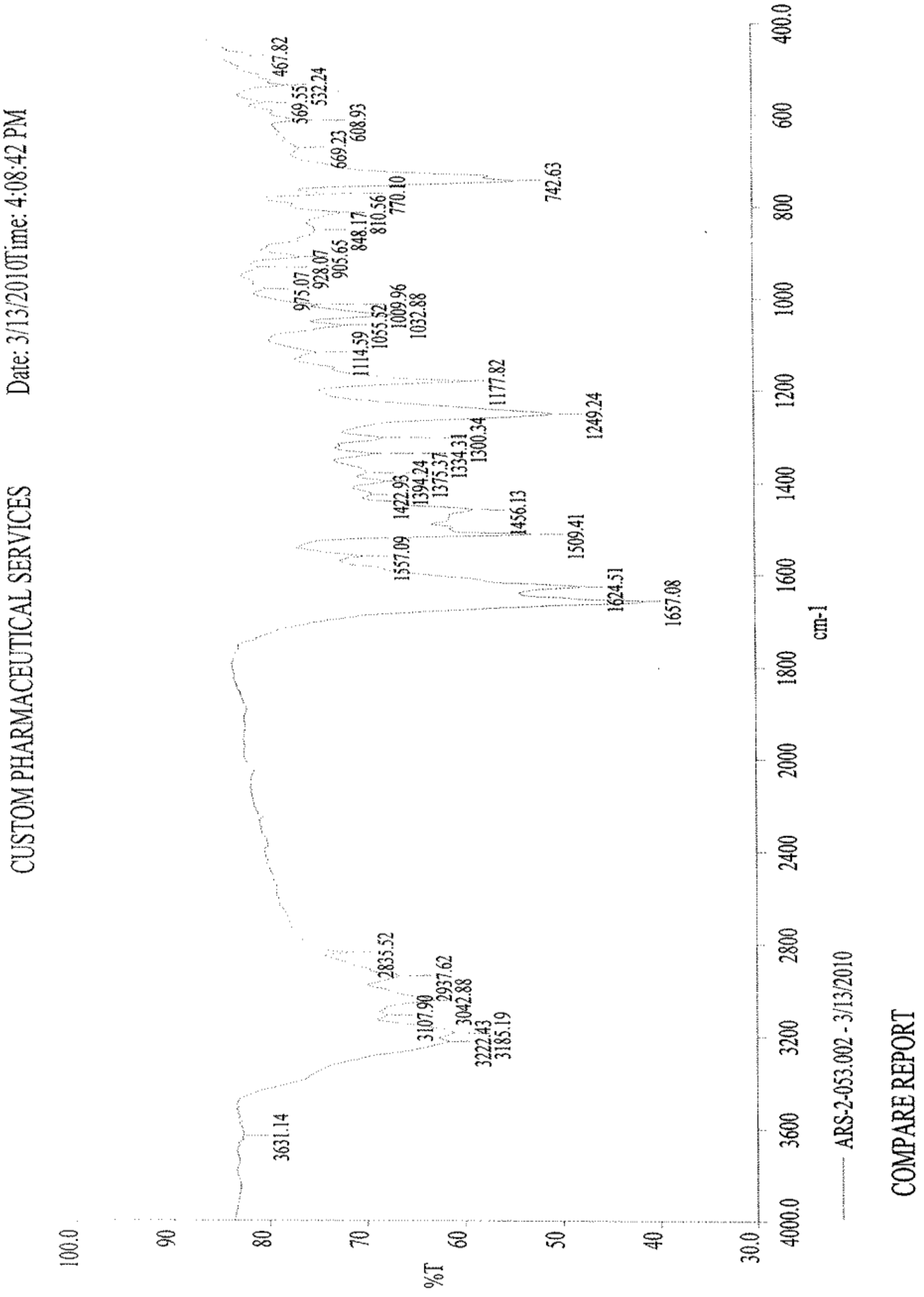

^{1}H NMR of (2,4-dichlorophenyl)(1-hydroxy-2,3,4,9-tetrahydro-1*H*-pyrido[3,4-b]indol-1-yl)methanone (**8g**):

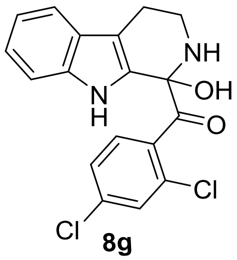

8g

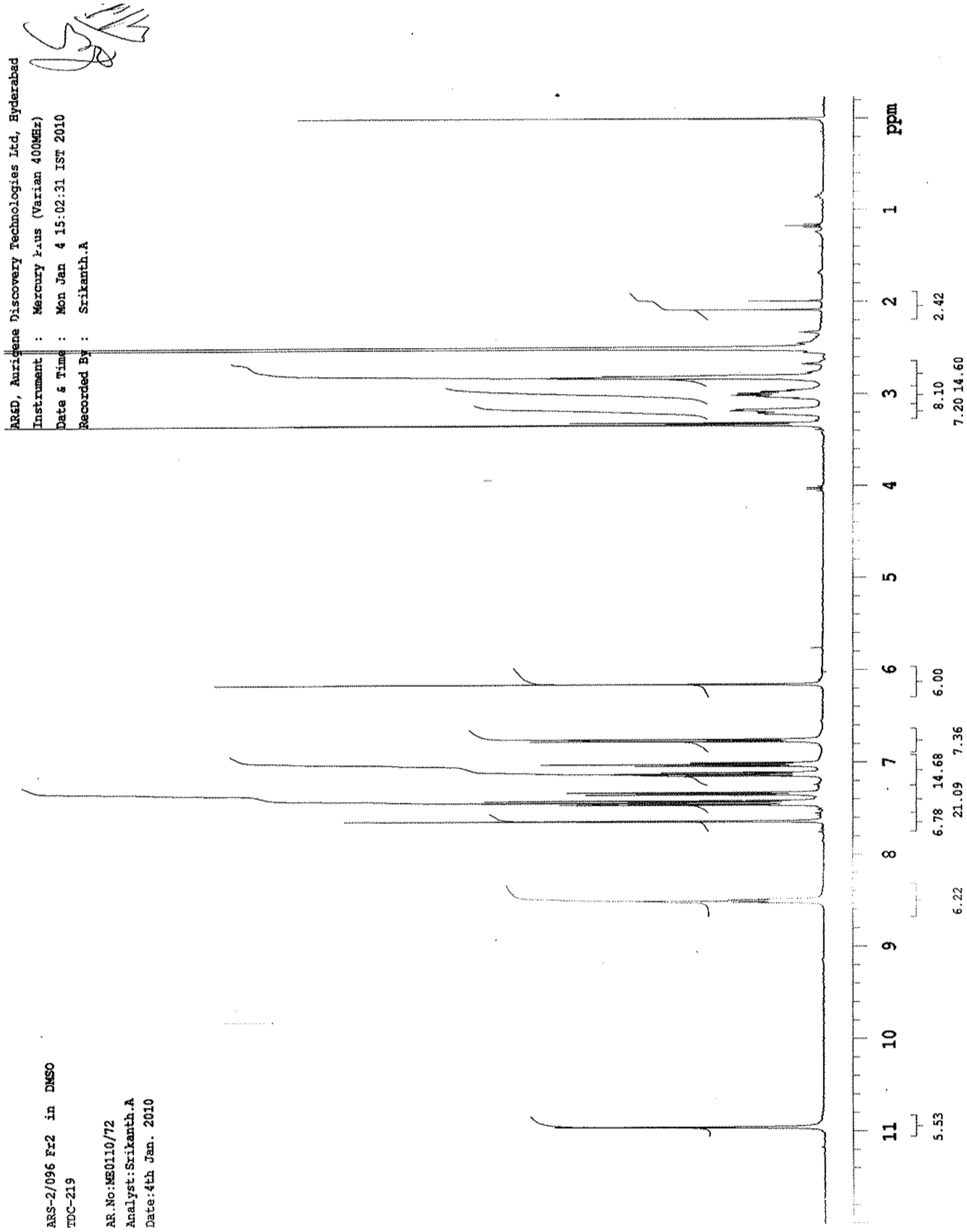

^{13}C NMR of (2,4-dichlorophenyl)(1-hydroxy-2,3,4,9-tetrahydro-1*H*-pyrido[3,4-b]indol-1-yl)methanone (**8g**):

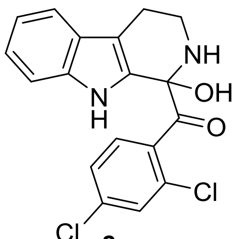

8g

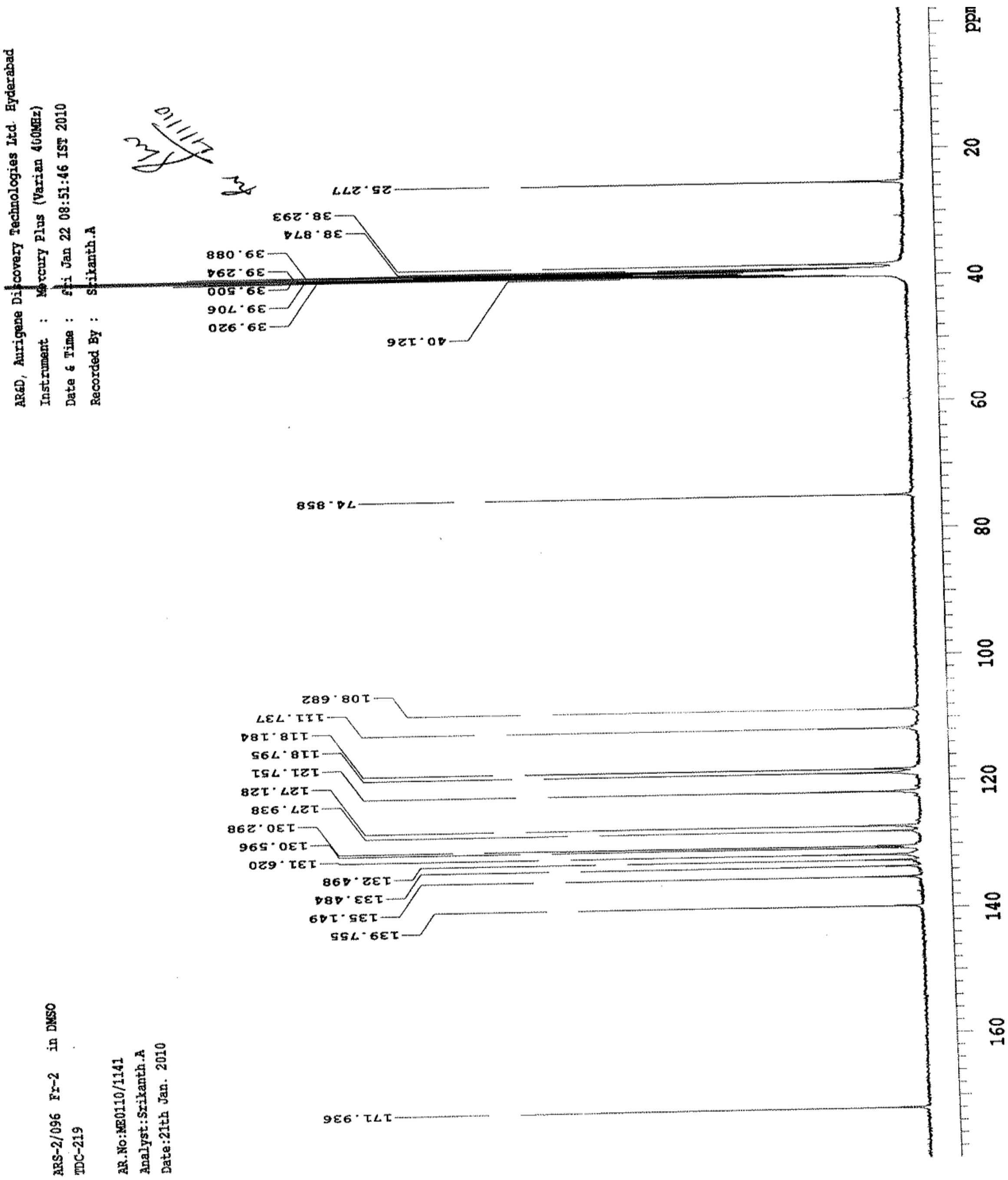

Mass spectrum (2,4-dichlorophenyl)(1-hydroxy-2,3,4,9-tetrahydro-1*H*-pyrido[3,4-b]indol-1-yl)methanone (**8g**):

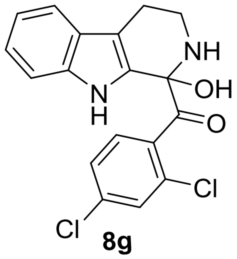

8g

CPS,MIYAPUR

Mass Analysis Report

**Data Filename**	100104014.d	**Sample Name**	ARS-2/096 FR-2
**Sample Type**	Sample	**Position**	Vial 14
**Instrument Name**	Instrument 1	**User Name**	
**Acq Method**	ESI.m	**IRM Calibration Status**	Success
**DA Method**	default.m	**Comment**	

User Spectra

Fragmentor Voltage	Collision Energy	Ionization Mode
110	0	Esi

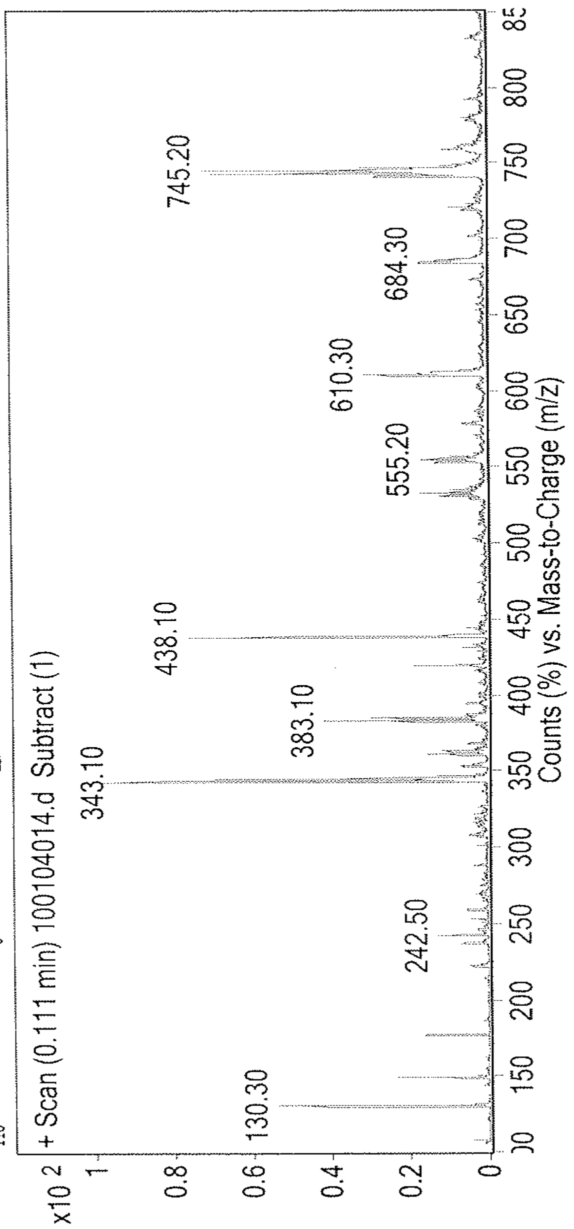

--- End Of Report ---

HRMS of (2,4-dichlorophenyl)(1-hydroxy-2,3,4,9-tetrahydro-1*H*-pyrido[3,4-b]indol-1-yl)methanone (**8g**):

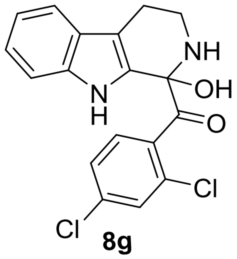

8g

Page 1

Elemental Composition Report

Single Mass Analysis
Tolerance = 5.0 PPM / DBE: min = -1.5, max = 80.0
Element prediction: Off
Number of isotope peaks used for i-FIT = 3

Monoisotopic Mass, Even Electron Ions
94 formula(e) evaluated with 2 results within limits (up to 4 best isotopic matches for each mass)
Elements Used:
C: 0-25 H: 0-25 N: 0-4 O: 0-2 Cl: 0-2
9g
UT1212_166 5 (0.197) Cm (5)

Gajanan
1: TOF MS ES+
1.02e+003

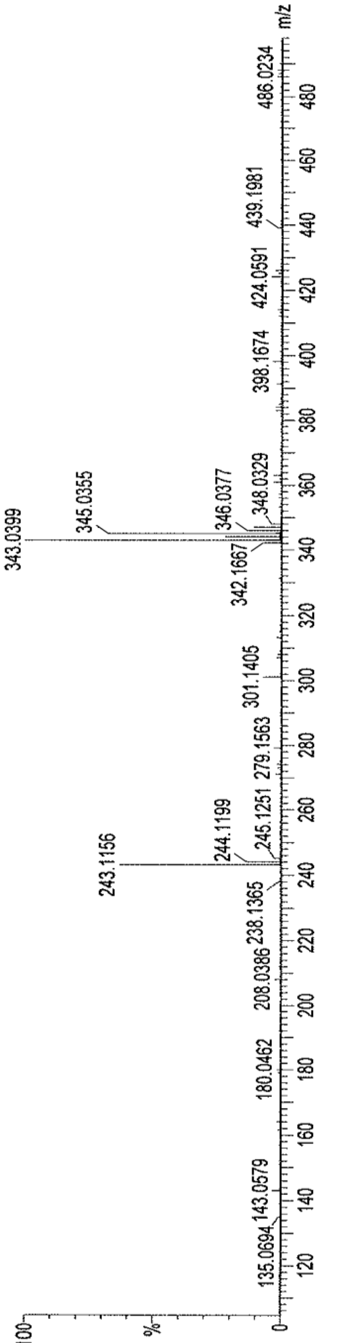

Minimum:				-1.5		
Maximum:		5.0	5.0	80.0		
Mass	Calc. Mass	mDa	PPM	DBE	i-FIT	Formula
343.0399	343.0405	-0.6	-1.7	12.5	0.5	C18 H13 N2 O Cl2
	343.0387	1.2	3.5	17.5	72.7	C19 H8 N4 O Cl

IR spectrum of (2,4-dichlorophenyl)(1-hydroxy-2,3,4,9-tetrahydro-1*H*-pyrido[3,4-b]indol-1-yl)methanone (**8g**):

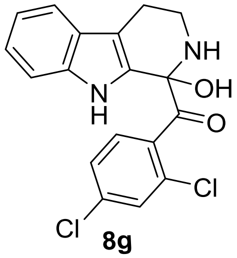

8g

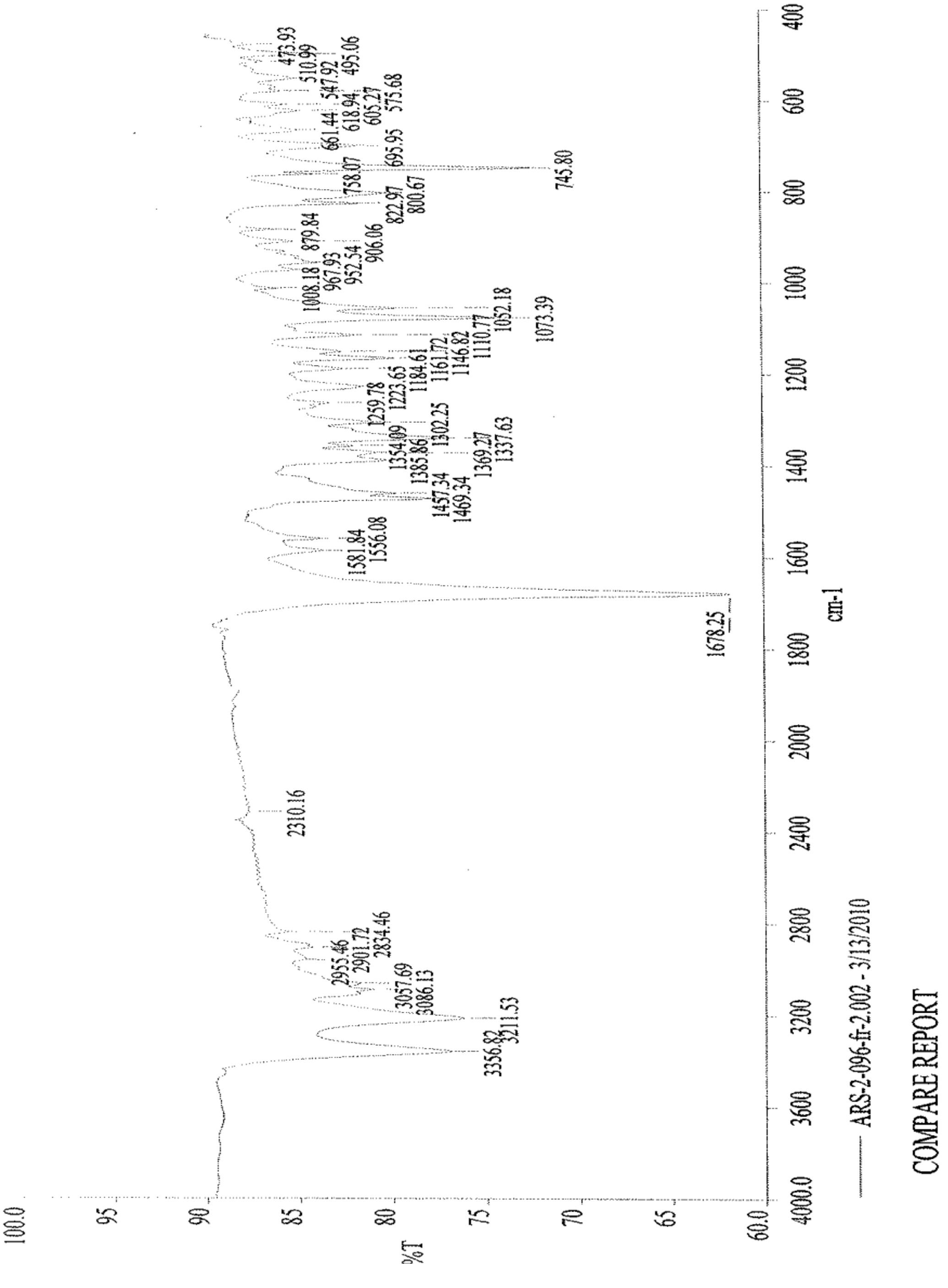

^{1}H NMR of (4-(benzyloxy)phenyl)(2,9-dihydro-1*H*-pyrido[3,4-b]indol-1-yl)methanone (**7h**):

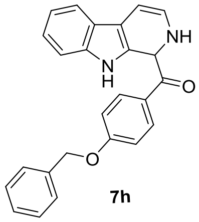

7h

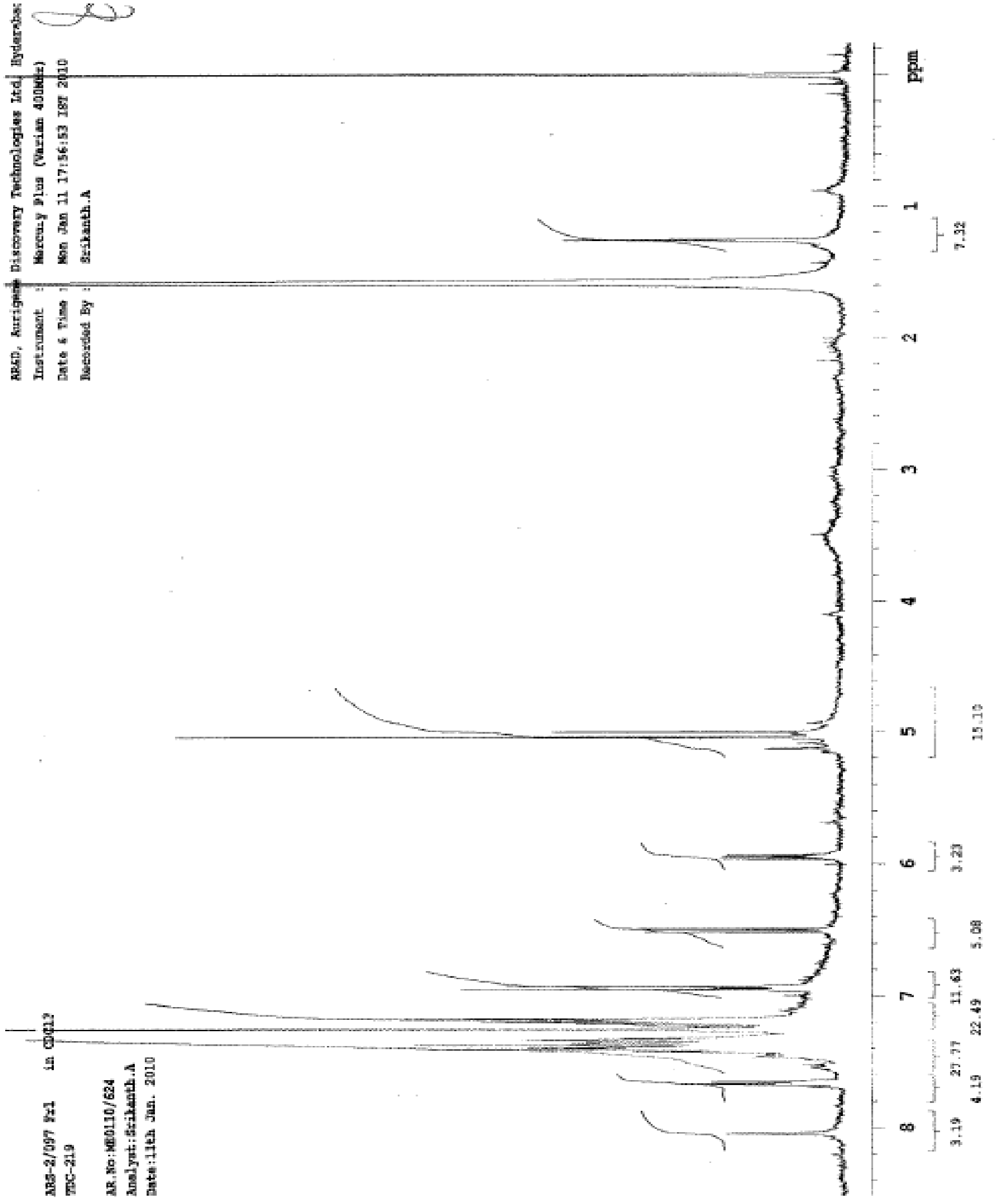

^{13}C NMR of (4-(benzyloxy)phenyl)(2,9-dihydro-1*H*-pyrido[3,4-b]indol-1-yl)methanone (**7h**):

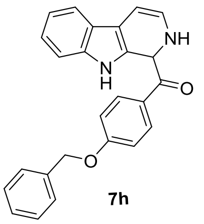

7h

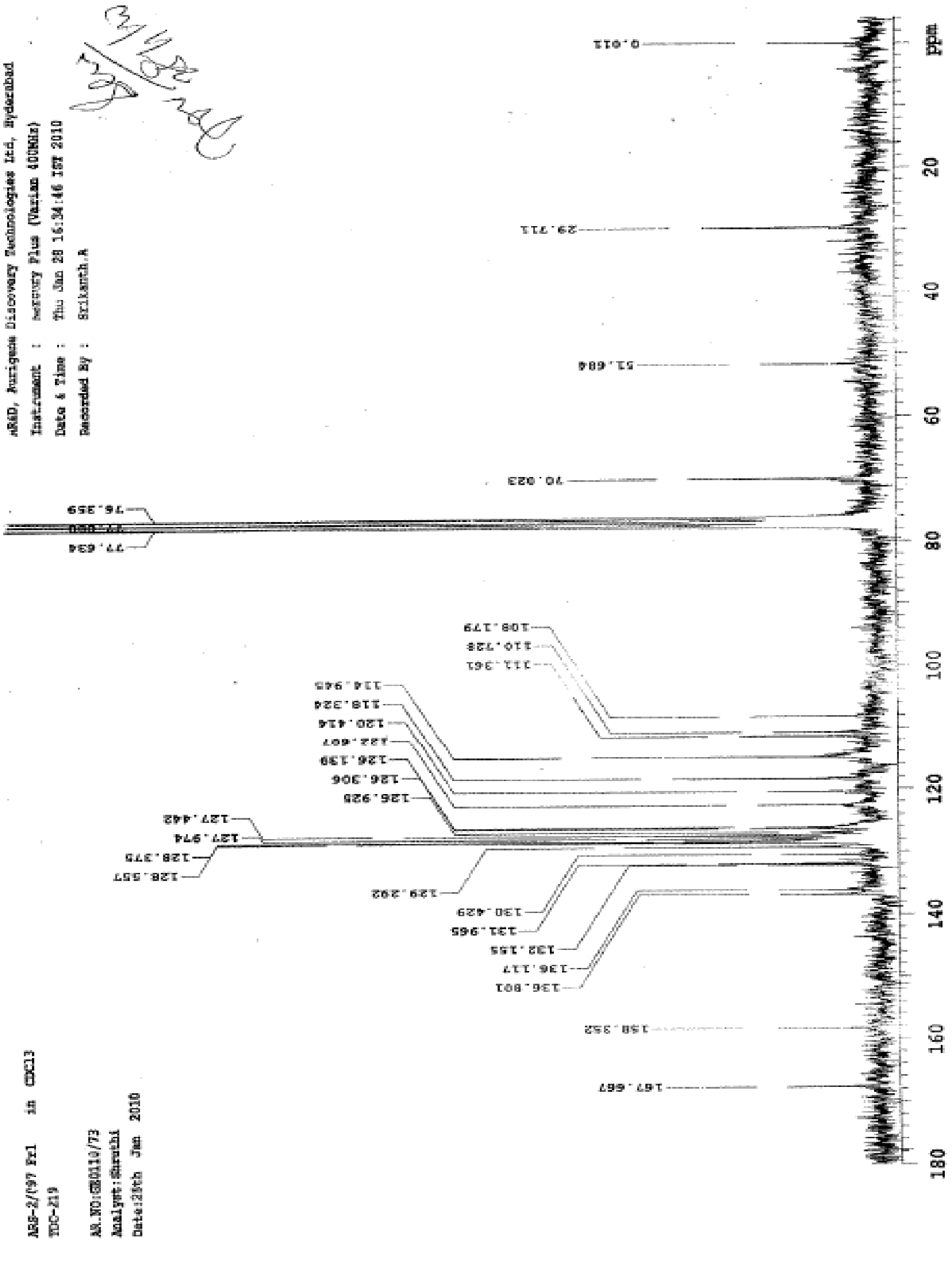

Mass spectrum of (4-(benzyloxy)phenyl)(2,9-dihydro-1*H*-pyrido[3,4-b]indol-1-yl)methanone (**7h**):

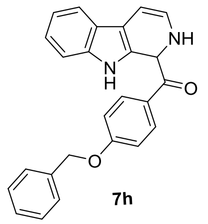

7h

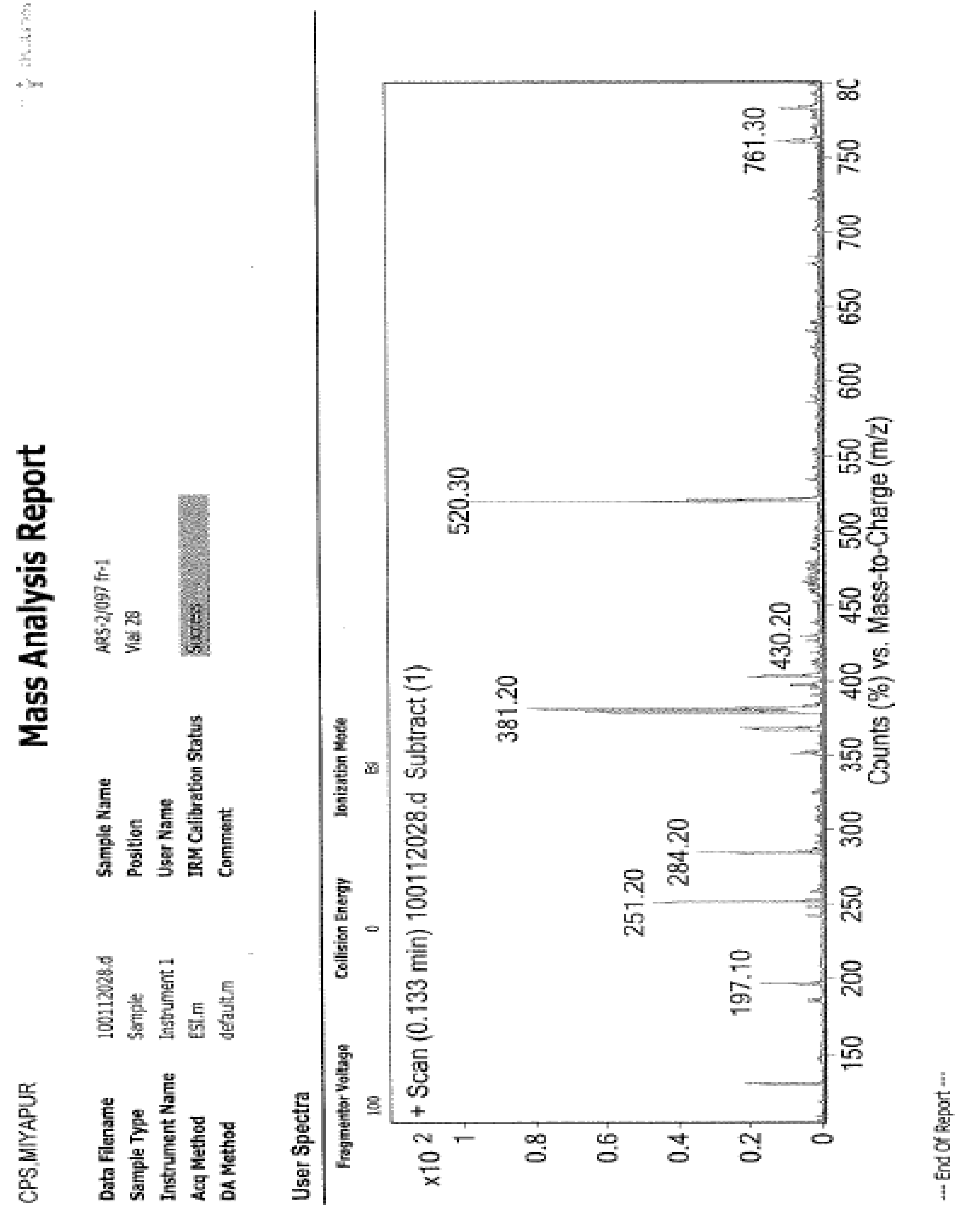

HRMS of (4-(benzyloxy)phenyl)(2,9-dihydro-1*H*-pyrido[3,4-b]indol-1-yl)methanone (**7h**):

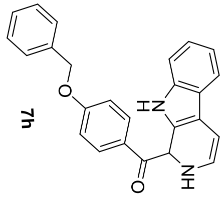

7h

Elemental Composition Report

Page 1

Single Mass Analysis

Tolerance = 6.0 PPM / DBE: min = -1.5, max = 80.0
Element prediction: Off
Number of isotope peaks used for i-FIT = 3

Monoisotopic Mass, Even Electron Ions
20 formula(e) evaluated with 1 results within limits (up to 4 best isotopic matches for each mass)
Elements Used:
C: 0-30 H: 0-25 N: 0-2 O: 0-2
9h
UT1212_178 6 (0.224) Cm (6:8)

Gajanan
1: TOF MS ES+
2.61e+003

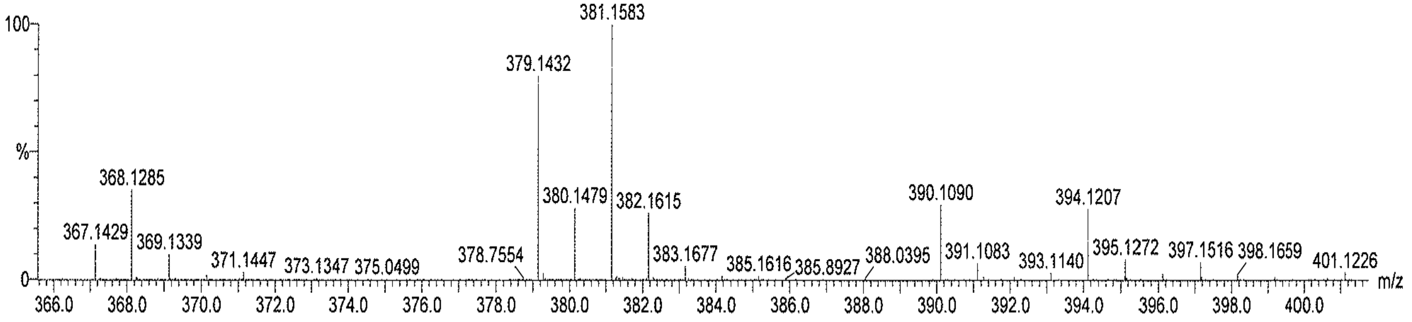

Minimum:				-1.5		
Maximum:		5.0	6.0	80.0		
Mass	Calc. Mass	mDa	PPM	DBE	i-FIT	Formula
381.1583	381.1603	-2.0	-5.2	16.5	6.5	C25 H21 N2 O2

^{1}H NMR of [1,1'-biphenyl]-4-yl(1-hydroxy-2,3,4,9-tetrahydro-1*H*-pyrido[3,4-b]indol-1-yl)methanone (**8i**):

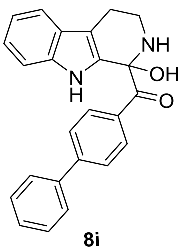

8i

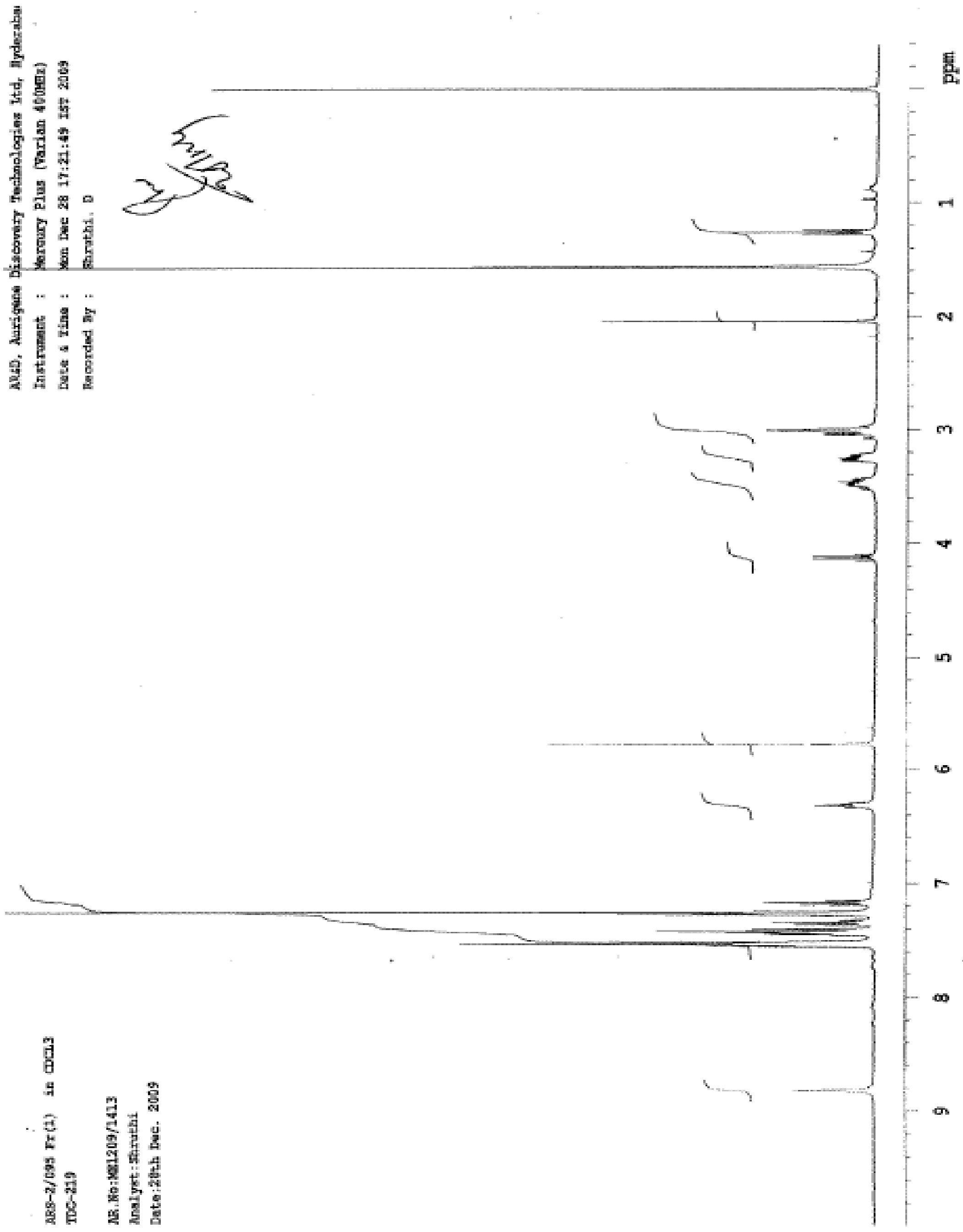

Mass spectrum of [1,1'-biphenyl]-4-yl(1-hydroxy-2,3,4,9-tetrahydro-1*H*-pyrido[3,4-b]indol-1-yl)methanone (**8i**):

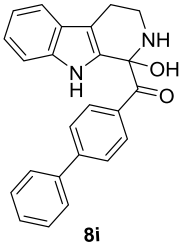

8i

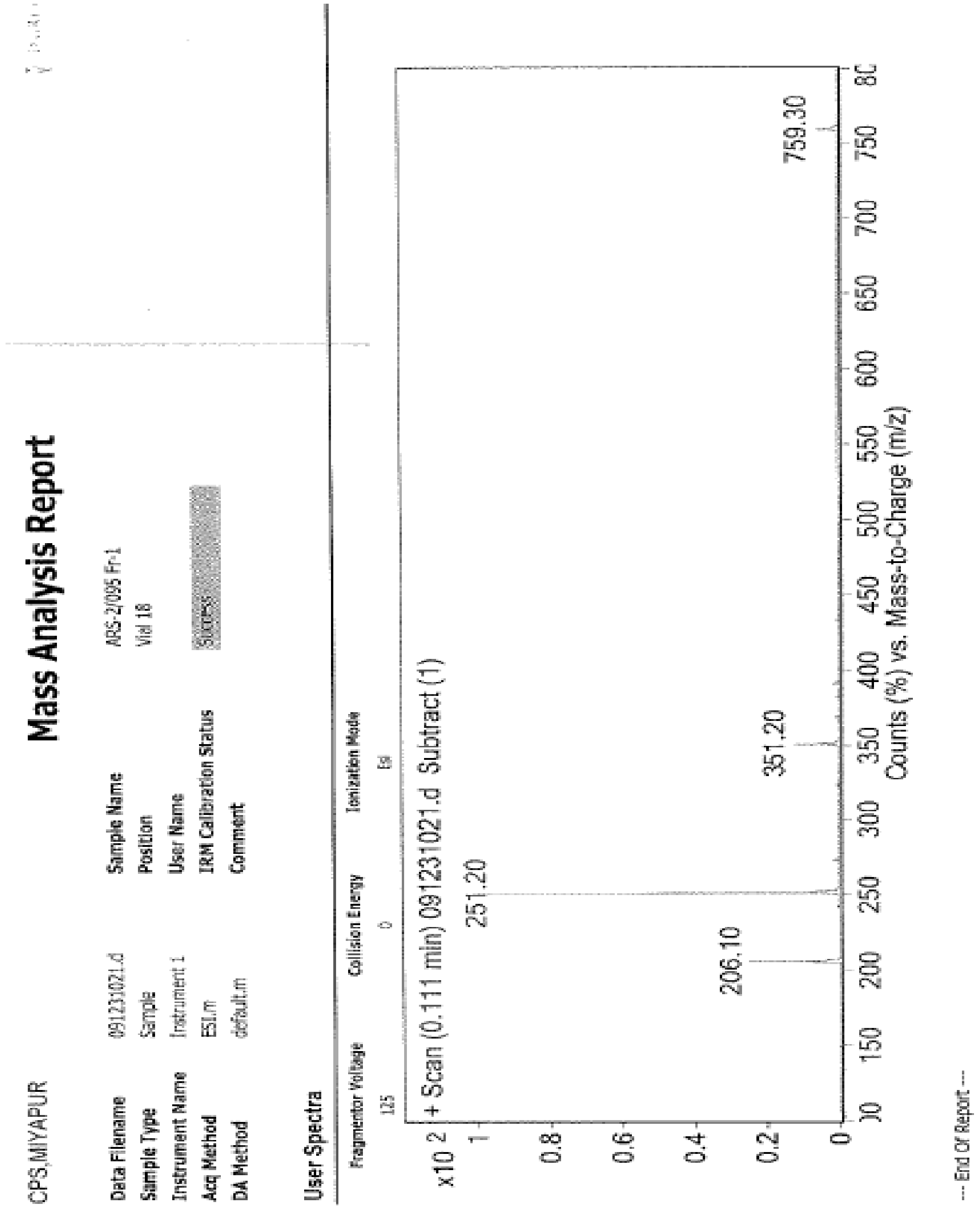

IR spectrum of [1,1'-biphenyl]-4-yl(1-hydroxy-2,3,4,9-tetrahydro-1*H*-pyrido[3,4-b]indol-1-yl)methanone (**8i**):

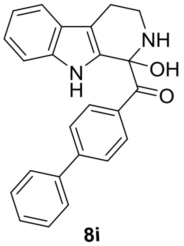

8i

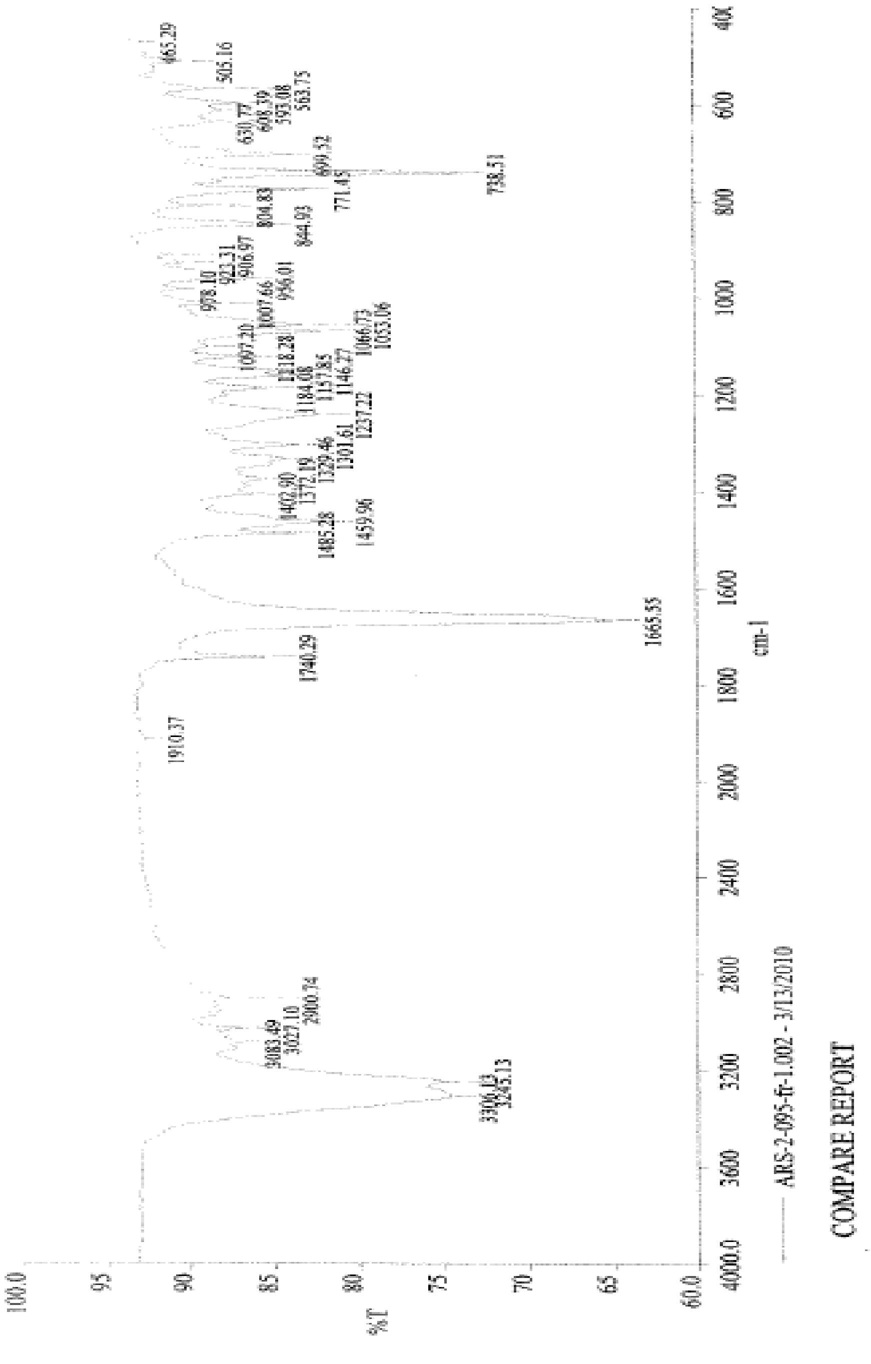

^{1}H NMR of (1-hydroxy-2,3,4,9-tetrahydro-1*H*-pyrido[3,4-b]indol-1-yl)(thiophen-2-yl)methanone (**8j**):

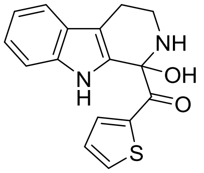

8j

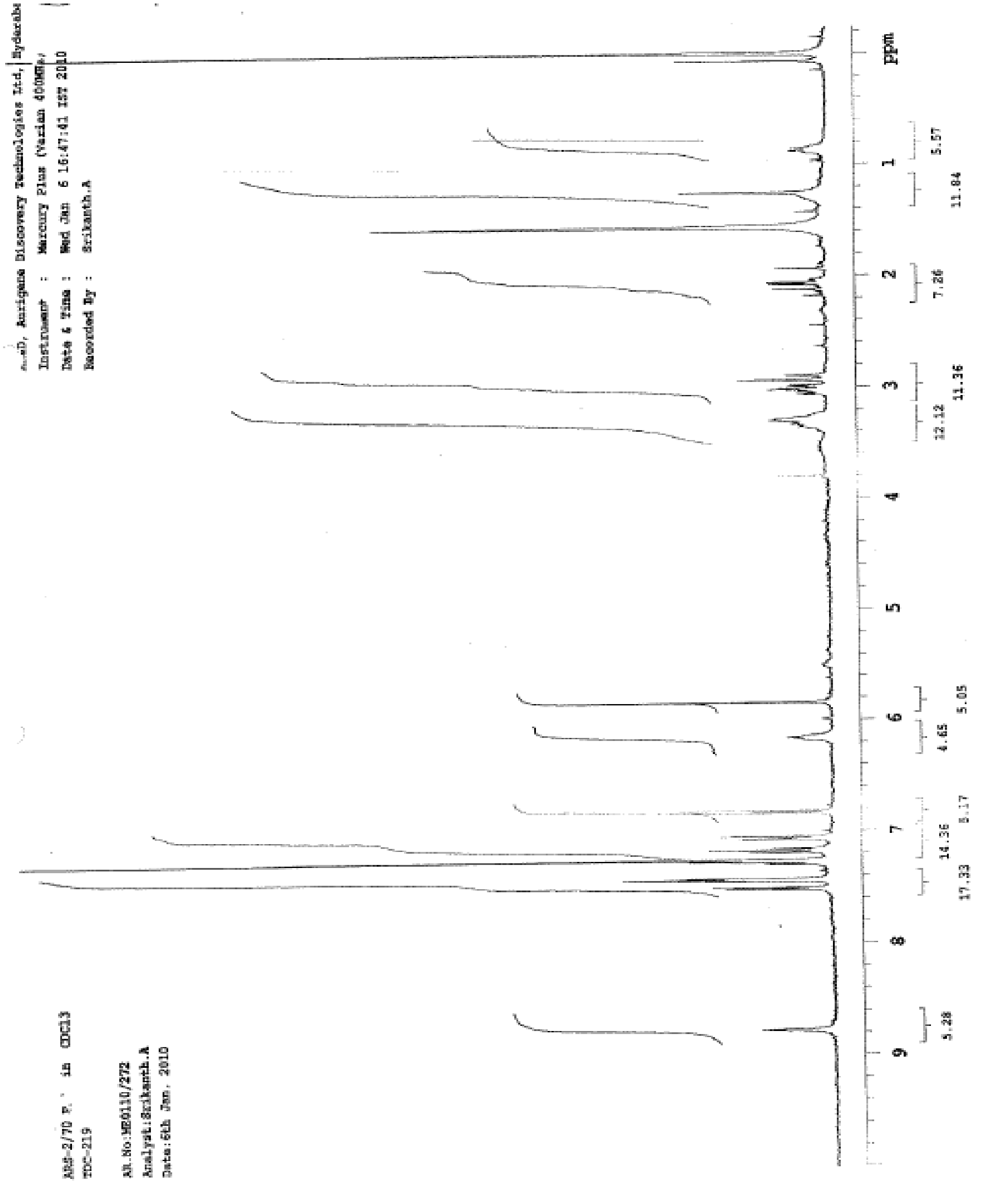

^{13}C NMR of (1-hydroxy-2,3,4,9-tetrahydro-1*H*-pyrido[3,4-b]indol-1-yl)(thiophen-2-yl)methanone (**8j**):

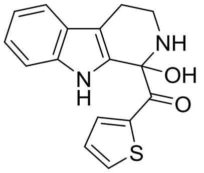

8j

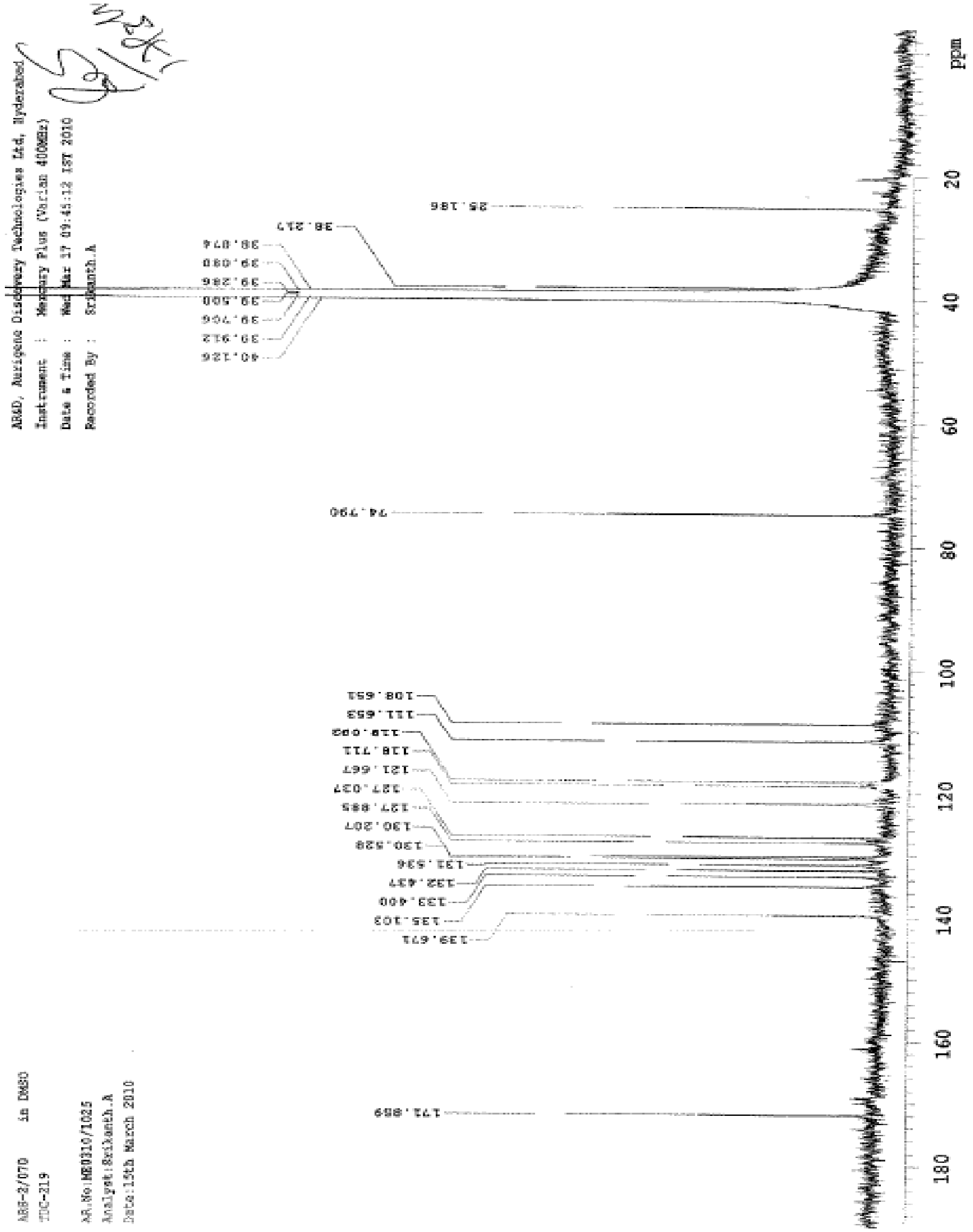

Mass spectrum of (1-hydroxy-2,3,4,9-tetrahydro-1*H*-pyrido[3,4-b]indol-1-yl)(thiophen-2-yl)methanone (**8j**):

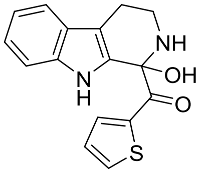

8j

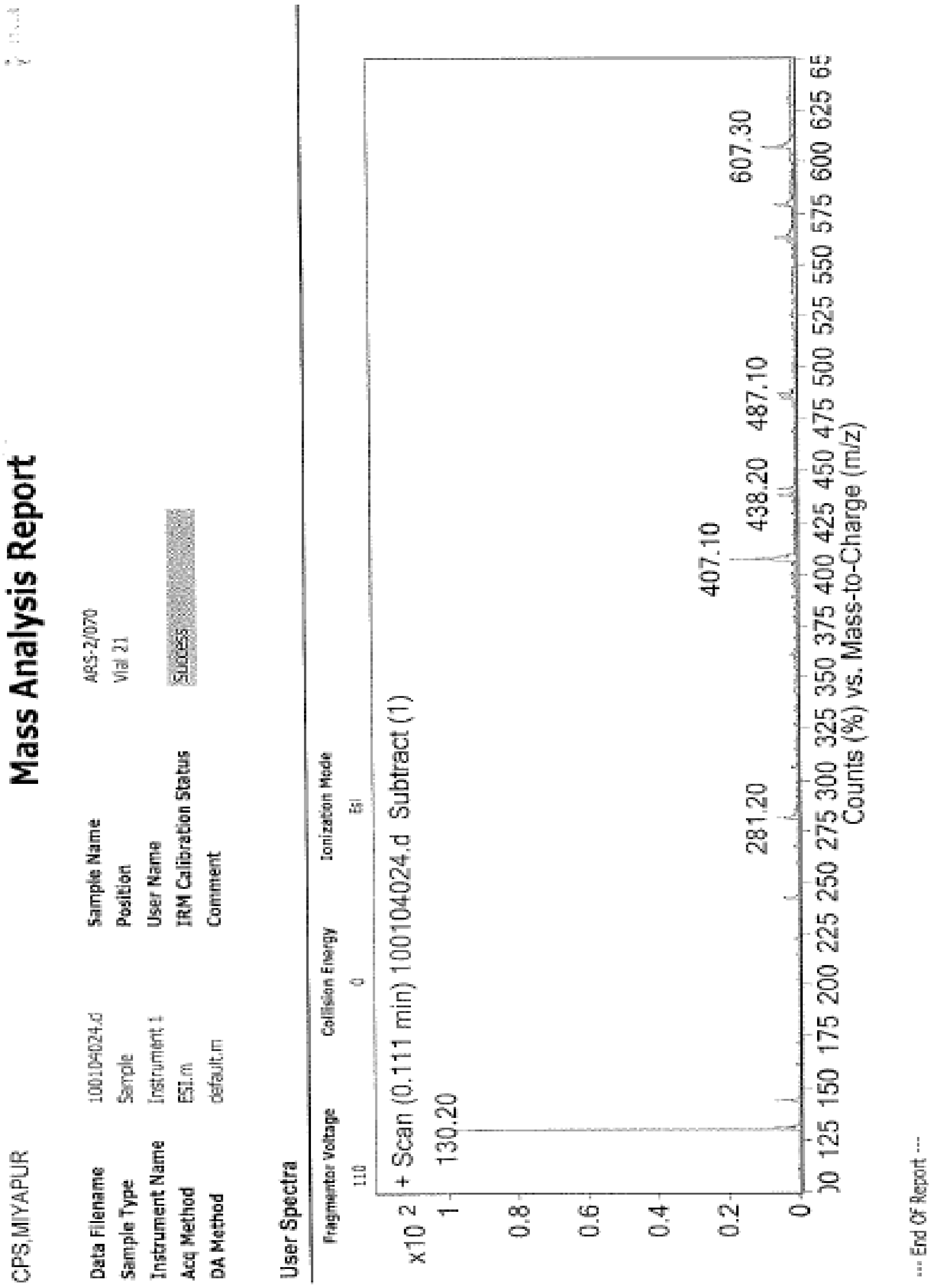

Supplement: File 1 — Experimental and analytical data. [file Beilstein_J_Org_Chem-10-471-s001.pdf]
